# Effects of pulmonary air leak on patients with coronavirus disease 2019 (COVID-19): a systematic review and meta-analysis

**DOI:** 10.1186/s12890-023-02710-2

**Published:** 2023-10-19

**Authors:** Zhuan Zhong, Jia Guo, Xingzhao Li, Yingying Han

**Affiliations:** 1 The Second Hospital of Jilin University, Changchun, Jilin Province China; 2 China-Japan Union Hospital of Jilin University, Changchun, Jilin Province China

**Keywords:** COVID-19, Meta-analysis, Pneumomediastinum, Pneumothorax, Subcutaneous Emphysema.

## Abstract

**Background:**

Coronavirus disease 2019 (COVID-19) has posed increasing challenges to global health systems. We aimed to understand the effects of pulmonary air leak (PAL), including pneumothorax, pneumomediastinum and subcutaneous emphysema, on patients with COVID-19.

**Methods:**

We searched PubMed, Embase and Web of Science for data and performed a meta-analysis with a random-effects model using Stata 14.0. This meta-analysis was conducted in accordance with the Preferred Reporting Items for Systematic Reviews and Meta-Analyses (PRISMA) guidelines.

**Results:**

Thirty-five articles were included in the meta-analysis. The data came from 14 countries and included 3,047 COVID-19 patients with PAL, 11,3679 COVID-19 patients without PAL and 361 non-COVID-19 patients with PAL. We found that the incidence of PAL was much higher in COVID-19 patients than in non-COVID-19 patients (odds ratio (OR) = 6.13, 95% CI: 2.09–18.00). We found that the group of COVID-19 patients with PAL had a longer hospital stay (standardized mean difference (SMD) = 0.79, 95% CI: 0.27–1.30) and intensive care unit (ICU) stay (SMD = 0.51, 95% CI: 0.19–0.83) and comprised more ICU (OR = 15.16, 95% CI: 6.51–35.29) and mechanical ventilation patients (OR = 5.52, 95% CI: 1.69–17.99); furthermore, the mortality rate was also higher (OR = 2.62, 95% CI: 1.80–3.82).

**Conclusions:**

Patients with lung injuries caused by COVID-19 may develop PAL. COVID-19 patients with PAL require more medical resources, have more serious conditions and have worse clinical outcomes.

**PROSPERO registration number:**

CRD42022365047.

**Supplementary Information:**

The online version contains supplementary material available at 10.1186/s12890-023-02710-2.

## Introduction

In December 2019, the first case of coronavirus disease 2019 (COVID-19) was diagnosed in Wuhan, China, and quickly spread worldwide [1]. As of December 19, 2022, there were a total of 653 million confirmed COVID-19 cases worldwide, of which 6.7 million were fatal cases [2]. COVID-19 is caused by SARS-CoV-2. Coronaviruses caused severe acute respiratory syndrome (SARS) in 2003 and Middle East respiratory syndrome (MERS) in 2012 [3, 4]. The source of SARS was civet cats, that of MERS was camels, and coronaviruses are transferred from bats to these animals [4, 5]; however, the source of COVID-19 is not yet clear. COVID-19 is a multisystem disease, and the lung is the most affected organ [6]. Pulmonary air leak (PAL), including pneumothorax, pneumomediastinum and subcutaneous emphysema, is a pulmonary complication of COVID-19 [7], and similar conditions occur in patients with SARS and MERS [8–10]. According to the study conducted by Shrestha et al., the incidence of PAL in patients with COVID-19 increases with the severity of the disease, ranging from 4.2 to 18.4% [11]. Patients with COVID-19 usually present with symptoms similar to those of patients with PAL, including tachycardia, tachypnoea, hypoxia and decreased breath sounds by auscultation, which are likely to result in a delayed diagnosis or misdiagnosis of PAL [12]. As COVID-19 is still a relatively new disease, the effects of PAL on COVID-19 patients are not completely clear. Therefore, we conducted a meta-analysis on data from COVID-19 patients with PAL to study the clinical features of these patients. This meta-analysis was conducted in accordance with the Preferred Reporting Items for Systematic Review and Meta-Analysis (PRISMA) guidelines. Our study was registered with the International Prospective Register of Systematic Reviews.

## Materials and methods

### Eligibility criteria

Studies that met the following criteria were included in the meta-analysis: (1) studies mainly including COVID-19 patients with PAL; (2) studies of patients with at least one type of PAL, including pneumothorax, pneumomediastinum and subcutaneous emphysema; (3) case‒control studies; and (4) studies describing the clinical characteristics or outcomes of patients.

The exclusion criteria were as follows: (1) nonhuman studies, (2) case reports, reviews and commentaries, (3) studies without control group data, (4) studies with duplicated data, and (5) studies with a sample size less than five.

### Information sources

We searched PubMed, Embase and Web of Science for studies published before December 19, 2022, that included COVID-19 patients with PAL. Language was not limited to identify more useful articles worldwide.

### Search strategy

For the term COVID-19, the search was limited to the title, and for the term PAL, it was limited to the title and abstract. The detailed search strategy is provided in Additional file 1.

### Study selection process

We imported all the literature retrieved from the databases into NoteExpress software. After removing duplicate articles, we conducted an initial screening by reading titles and abstracts to remove articles that were not relevant to our study. Then, by reading the full texts, articles for which data could not be extracted were further removed.

### Data selection process and items

Data extraction was completed by two authors. They determined the data items for meta-analysis through discussion, extracted the data independently by reading the full texts, and then compared the results. If the data were consistent, they were included in the meta-analysis. If discrepancies occurred, they were resolved through discussion and a third author made the final decision.

The following information was extracted from the literature and ultimately included in the meta-analysis: study design, diagnosis method, PAL type, author, country, publication year, study period, sample size, age, sex, the number of patients with diabetes, hypertension, chronic obstructive pulmonary disease (COPD), asthma, cancer and a history of smoking, the number of patients in the intensive care unit (ICU), length of ICU stay, length of hospital stay, the number of deaths, the number of patients with mechanical ventilation (MV), positive end-expiratory pressure (PEEP), peak inspiratory pressure, PaO_2_/FiO_2_ ratio, tidal volume, and various laboratory findings, including leucocyte, neutrophil, and lymphocyte counts and C-reactive protein, ferritin, platelet, D-dimer, aspartate aminotransferase and lactate dehydrogenase levels.

### Study risk of bias assessment

The Newcastle–Ottawa Quality Assessment Scale was used to independently assess the quality and risk of bias of the included studies. If the total score was equal to or higher than seven points, the included article was considered to have a low risk of bias and be of high quality. This assessment was performed by one author and checked by another.

### Reporting bias assessment

We used funnel plots (number of events ≥ 10) and Egger’s test for reporting bias assessment, and a p value < 0.05 indicated the presence of bias.

### Statistical analysis

Odds ratios (ORs) and standardized mean differences (SMDs) were used for data analysis and evaluation, in which ORs were used for dichotomous variables, SMDs were used for continuous variables, and the confidence intervals (CIs) were set at 95%. For data including only the sample size and quartile, we used the transformation formula to determine the mean and standard deviation [13]. The I^2^ statistic was used to quantify heterogeneity among studies, with I^2^ ≤ 50% indicating low heterogeneity, 50%<I^2^ ≤ 75% indicating moderate heterogeneity, and I^2^ > 75% indicating high heterogeneity [14]. A random-effects model was used to estimate the effect value. The statistical software was Stata 14.0, and a z test p value < 0.05 was considered to indicate statistical significance.

## Results

### Study selection

**Fig. 1:**
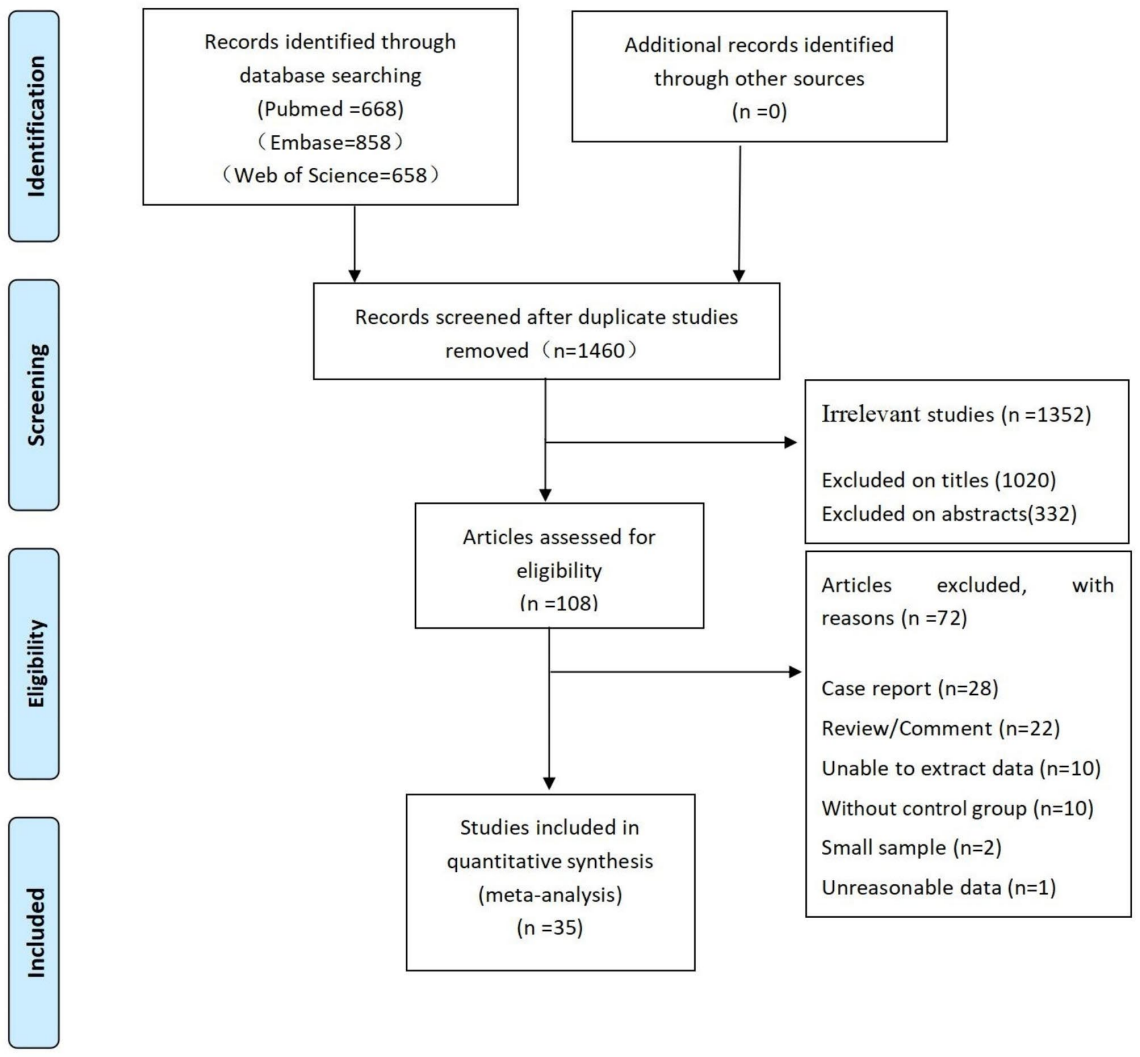
Flow diagram of study selection

A total of 2,184 articles were retrieved from the databases, including 668 from PubMed, 858 from Embase, and 658 from Web of Science. After importing the data into NoteExpress software, 724 duplicate articles were deleted. By reading titles and abstracts, 1,352 irrelevant articles were further eliminated, and of the remaining 108 articles, 73 articles were removed after reading the full text. The flow diagram of the study selection is shown in Fig. 1.

### Risk of bias in the studies

According to the Newcastle–Ottawa Quality Assessment Scale, we found that most of the literature included in the meta-analysis was of high quality and had a low risk of bias (Additional file 3: Table S1).

### Characteristics and results of individual studies

Thirty-five articles were included in the meta-analysis. The data came from 14 countries and included 3,047 COVID-19 patients with PAL, 11,3679 COVID-19 patients without PAL and 361 non-COVID-19 patients with PAL. Information on the characteristics and results of the individual studies is detailed in Table 1.

**Table 1:** Characteristics of individual studies

Author	Country	Year	Study design	Adult (≥ 18)	All need MV	All need ICU	Diagnosis method of COVID-19	Diagnosis method of PAL	Types of PAL
Geraci et al. [12]	USA	2021	retrospective	yes	no	no	RT-PCR	XR/CT	pneumothorax
Malik et al. [15]	USA	2022	retrospective	yes	no	no	RT-PCR	NA	pneumothorax
Pierre et al. [16]	USA	2022	retrospective	NA	no	no	NA	XR/CT	pneumothorax
Akram et al. [17]	Qatar	2022	retrospective	≥ 14	no	yes	RT-PCR	XR/CT/lung ultrasound	pneumothorax
Miro et al. [18]	Spain	2020	retrospective	NA	NA	no	RT-PCR	XR/CT	pneumothorax
Berg et al. [19]	USA	2021	retrospective	yes	no	yes	NA	XR/CT	Pneumothorax
Chopra et al. [20]	USA	2021	retrospective	yes	yes	yes	RT-PCR	NA	pneumothorax
Ozdemir et al. [21]	Turkey	2020	retrospective	yes	yes	yes	RT-PCR	XR/CT/lung ultrasound	pneumothorax
Capaccione et al. [22]	USA	2021	retrospective	NA	yes	NA	NA	XR	pneumothorax
Taha et al. [23]	USA	2022	retrospective	yes	yes	yes	RT-PCR	NA	pneumothorax
Reis et al. [24]	USA	2021	retrospective	NA	no	no	RT-PCR	XR	pneumomediastinum
Arciniega et al. [25]	Mexico	2021	retrospective	yes	no	no	RT-PCR	CT	pneumomediastinum
Ozdemir et al. [26]	Turkey	2021	retrospective	yes	yes	yes	RT-PCR	XR/CT	pneumomediastinum
Ozsoy et al. [27]	Turkey	2021	retrospective	NA	NA	NA	NA	CT	pneumomediastinum
Baslas et al. [28]	UK	2022	retrospective	yes	no	NA	RT-PCR/CT	CT	pneumomediastinum
Loffi et al. [29]	Italy	2020	retrospective	NA	no	no	RT-PCR	CT	pneumomediastinum
Righetti et al. [30]	Italy	2022	NA	NA	yes	yes	NA	NA	pneumomediastinum
Muhammad et al. [31]	UK	2022	retrospective	yes	no	no	RT-PCR	XR/CT	pneumothorax/pneumomediastinum
Tetaj et al. [32]	Italy	2021	retrospective	NA	yes	NA	RT-PCR	CT	pneumothorax/pneumomediastinum
Udwadia et al. [33]	India	2021	retrospective	NA	NA	yes	NA	XR/CT	pneumothorax/pneumomediastinum
Gazivoda et al. [34]	USA	2021	retrospective	NA	yes	yes	RT-PCR	XR	pneumothorax/pneumomediastinum
Belletti et al. [35]	Italy	2021	retrospective	yes	yes	yes	RT-PCR	XR/CT	pneumothorax/pneumomediastinum
Ernst et al. [36]	USA	2021	retrospective	NA	no	no	RT-PCR	NA	pneumothorax/pneumomediastinum
Bonato et al. [37]	Italy	2021	retrospective	NA	no	NA	RT-PCR	XR/CT	pneumothorax/pneumomediastinum
Tonelli et al. [38]	Italy	2022	retrospective	NA	yes	yes	RT-PCR	CT	pneumothorax/pneumomediastinum
Marza et al. [39]	Romania	2022	retrospective	yes	no	no	RT-PCR	XR/CT	pneumothorax/pneumomediastinum
Shaikh et al. [40]	Qatar	2021	retrospective	yes	NA	yes	NA	XR/CT	pneumothorax/pneumomediastinum
Lemmers et al. [41]	Italy	2020	retrospective	yes	yes	yes	RT-PCR	XR/CT	pneumomediastinum/subcutaneous emphysema
Steinberger et al. [42]	USA	2022	retrospective	yes	yes	NA	RT-PCR	XR/CT	pneumomediastinum/subcutaneous emphysema
Nespoli et al. [43]	Italy	2020	retrospective	NA	NA	yes	NA	XR/CT/lung ultrasound	pneumothorax/pneumomediastinum/subcutaneous emphysema
Hamouri et al. [44]	Jordan	2021	retrospective	yes	yes	no	RT-PCR	XR	pneumothorax/pneumomediastinum/subcutaneous emphysema
Guven et al. [45]	Turkey	2021	retrospective	yes	yes	no	RT-PCR	XR/CT	pneumothorax/pneumomediastinum/subcutaneous emphysema
Jones et al. [46]	UK	2020	retrospective	NA	yes	yes	RT-PCR	XR	pneumothorax/pneumomediastinum/subcutaneous emphysema
Venkateswaran et al. [47]	India	2022	retrospective	yes	yes	yes	NA	XR/CT	pneumothorax/pneumomediastinum/subcutaneous emphysema
Sami et al. [48]	Iran	2022	retrospective	NA	yes	yes	RT-PCR	XR/CT	pneumothorax/pneumomediastinum/subcutaneous emphysema

COVID-19: coronavirus disease 2019, MV: mechanical ventilation, ICU: intensive care unit, PAL: pulmonary air leak, RT-PCR: reverse transcriptase-polymerase chain reaction, CT: computed tomography, XR: X-ray, NA: not applicable, USA: United States of America, UK: United Kingdom

## Results of syntheses

### The incidence of PAL

**Fig. 2:**
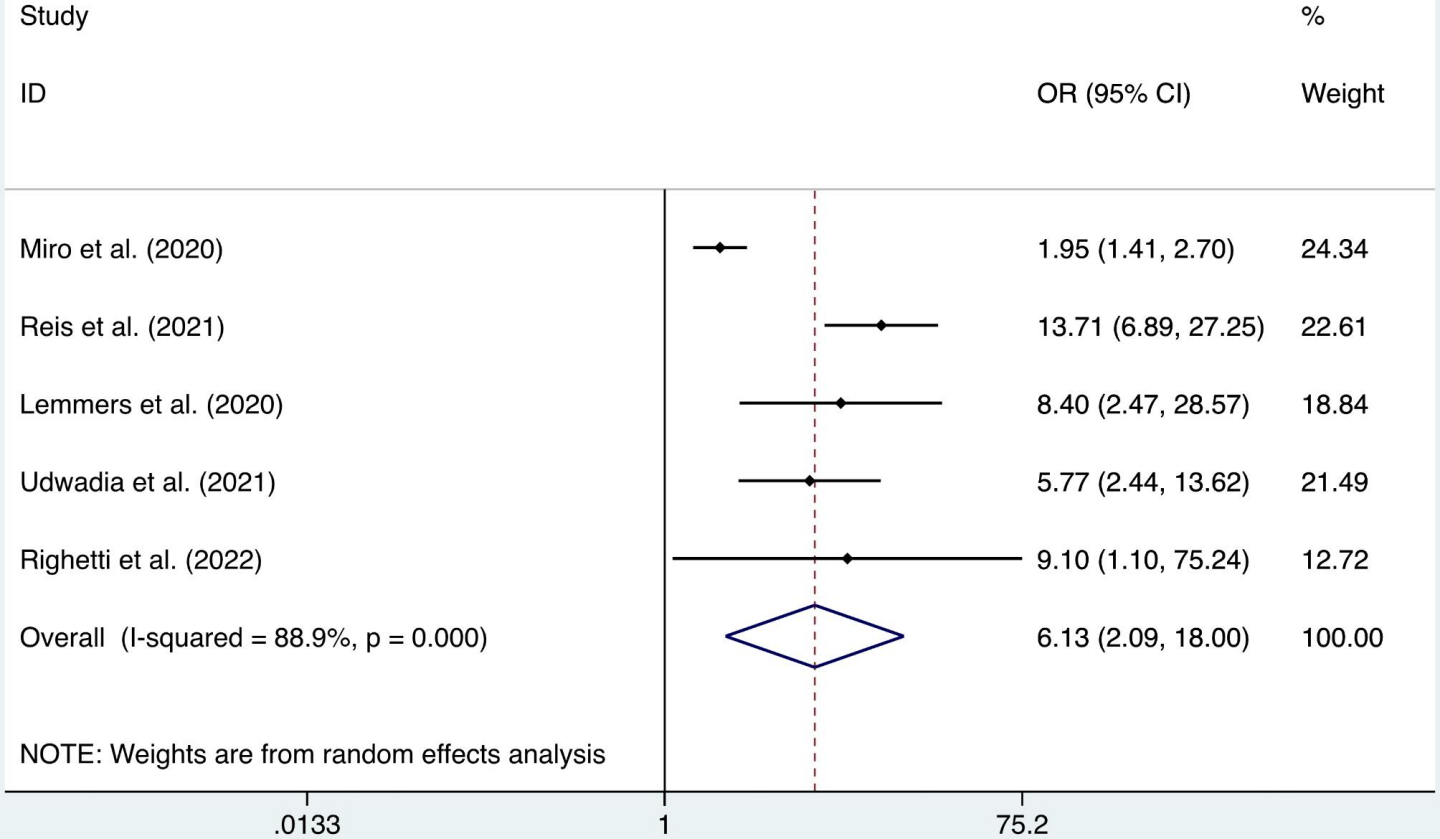
Forest plot of the incidence of pulmonary air leak (number of events: 5)

A total of five articles were used to study the effect of COVID-19 infection on the incidence of PAL (Table 2). The participants included in the experimental group were all COVID-19 patients, while none of the patients in the control group were infected with SARS-CoV-2. We found that the incidence of PAL was much higher in COVID-19 patients than in non-COVID-19 patients (OR = 6.13, 95% CI: 2.09–18.00, I^2^ = 88.9%, p = 0.001, Fig. 2).

**Table 2:** Characteristics of pulmonary air leak patients with COVID-19 and non-COVID-19.

Author	COVID-19		non-COVID-19	
	Number of COVID-19	Number of PAL	Number of non-COVID-19	Number of PAL
Miro et al. [18]	71,904	40	1,358,134	387
Reis et al. [24]	5687	87	7949	9
Righetti et al. [30]	59	8	59	1
Udwadia et al. [33]	1324	42	1062	6
Lemmers et al. [41]	169	23	163	3

COVID-19: coronavirus disease 2019, PAL: pulmonary air leak

### Clinical characteristics

A total of 33 articles were included to study the clinical characteristics of patients with PAL. All patients in the experimental group had COVID-19 with PAL, while the control group had COVID-19 but did not suffer from the corresponding type of PAL (Additional file 3: Tables S2–S5).

#### Age, sex and hospital admission information

The data of control patients in four articles were matched according to age [36–38, 47], those of control patients in three articles were matched according to sex [36, 37, 47], and another article included data on the age and sex characteristics of patients with haemothorax [45]. Therefore, we excluded information from the above literature when studying the effect of age and sex on the development of PAL in COVID-19 patients. We found that males (OR = 1.38, 95% CI: 1.10–1.74, I^2^ = 54.1%, p = 0.005, Additional file 3: Figure S1) and younger individuals (SMD=-0.20, 95% CI: -0.32–0.07, I^2^ = 73.1%, p = 0.002, Additional file 3: Figure S2) were more likely to develop PAL. If all patients in a study were from ICU wards, their data were not included in the study of the ICU admission rate. After screening, six articles were included, which showed that the ICU admission rate of the experimental group was significantly higher than that of the control group (OR = 15.16, 95% CI: 6.51–35.29, I^2^ = 75.5%, p < 0.001, Additional file 3: Figure S3). At the same time, we also found that COVID-19 patients with PAL had a longer length of ICU stay (SMD = 0.51, 95% CI: 0.19–0.83, I^2^ = 85.5%, p = 0.002, Additional file 3: Figure S4) and hospital stay (SMD = 0.79, 95% CI: 0.27–1.30, I^2^ = 97.7%, p = 0.003, Additional file 3: Figure S5).

#### Comorbidities

In terms of comorbidities, our study showed that the prevalence of diabetes (OR = 0.67, 95% CI: 0.80–0.67, I^2^ = 22.2%, p = 0.014, Additional file 3: Figure S6) and hypertension (OR = 0.69, 95% CI: 0.56–0.84, I^2^ = 38.7%, p < 0.001, Additional file 3: Figure S7) was lower in COVID-19 patients with PAL; however, COPD (OR = 0.85, 95% CI: 0.53–1.35, I^2^ = 33.3%, p = 0.486, Additional file 3: Figure S8), asthma (OR = 1.76, 95% CI: 0.95–3.27, I^2^ = 75.0%, p = 0.072, Additional file 3: Figure S9) and cancer (OR = 1.16, 95% CI: 0.90–1.15, I^2^ = 1.8%, p = 0.254, Additional file 3: Figure S10) did not affect the development of PAL in COVID-19 patients. We also compared the differences in smoking between the experimental group and the control group and found that smoking (OR = 1.00, 95% CI: 0.88–1.14, I^2^ = 2.9%, p = 0.974, Additional file 3: Figure S11) did not increase the risk of PAL in COVID-19 patients.

#### Laboratory findings

Through the analysis of a large number of patients with laboratory results, we found that the presence of PAL was associated with increased D-dimer levels (SMD = 0.74, 95% CI: 0.34–1.14, I^2^ = 83.2%, p < 0.001, Additional file 3: Figure S12), leucocyte counts (SMD = 0.57, 95% CI: 0.26–0.87, I^2^ = 67.2%, p < 0.001, Additional file 3: Figure S13), aspartate aminotransferase levels (SMD = 0.57, 95% CI: 0.31–0.84, I^2^ = 0, p < 0.001, Additional file 3: Figure S14) and lactate dehydrogenase levels (SMD = 0.35, 95% CI: 0.03–0.67, I^2^ = 73.6%, p = 0.031, Additional file 3: Figure S15) in patients with COVID-19. However, for neutrophil counts (SMD = 0.19, 95% CI: 0.00–0.39, I^2^ = 0, p = 0.05, Additional file 3: Figure S16), lymphocyte counts (SMD = 0.07, 95% CI: -0.33–0.47, I^2^ = 84.6%, p = 0.727, Additional file 3: Figure S17), C-reactive protein levels (SMD = 0.21, 95% CI: 0.05–0.47, I^2^ = 74%, p = 0.109, Additional file 3: Figure S18), ferritin levels (SMD = 0.59, 95% CI: 0.11–1.28, I^2^ = 89.7%, p = 0.099, Additional file 3: Figure S19), platelet counts (SMD = 0.13, 95% CI: 0.40–0.15, I^2^ = 74.0%, p = 0.376, Additional file 3: Figure S20) and creatinine levels (SMD = 0.59, 95% CI: 1.47–0.29, I^2^ = 83.2%, p = 0.187, Additional file 3: Figure S21), there was no significant impact.

#### MV

When studying whether PAL affected the number of COVID-19 patients who needed MV, we excluded two types of studies: (1) studies in which all patients had MV [20–23, 26, 30, 32, 34, 35, 38, 41, 42, 44–49], and (2) studies in which the number of patients with MV in the experimental and control groups was matched [24]. A total of five studies were included, and it was found that a higher number of COVID-19 patients with PAL required MV (OR = 5.52, 95% CI: 1.69–17.99, I^2^ = 89.5%, p = 0.005, Additional file 3: Figure S22). After excluding studies in which all patients needed invasive MV [21, 22, 41] or noninvasive MV [38], six articles were included, and the analysis showed that among COVID-19 patients with MV, those with PAL did not require more invasive MV (OR = 0.93, 95% CI: 0.43–1.99, I^2^ = 62.6%, p = 0.850, Additional file 3: Figure S23). With regard to ventilator parameters, we found that COVID-19 patients with PAL had higher PEEP values (SMD = 0.25, 95% CI: 0.03–0.47, I^2^ = 61.6%, p = 0.026, Additional file 3: Figure S24), but there were no significant differences in peak inspiratory pressure (SMD = 0.53, 95% CI: -0.08–1.14, I^2^ = 85.7%, p = 0.086, Additional file 3: Figure S25), PaO_2_/FiO_2_ ratio (SMD=-0.30, 95% CI: -0.64–0.04, I^2^ = 77.9%, p = 0.084, Additional file 3: Figure S26) and tidal volume (SMD = 0.14, 95% CI: -0.11–0.39, I^2^ = 66.4%, p = 0.264, Additional file 3: Figure S27) values.

#### Mortality

**Fig. 3:**
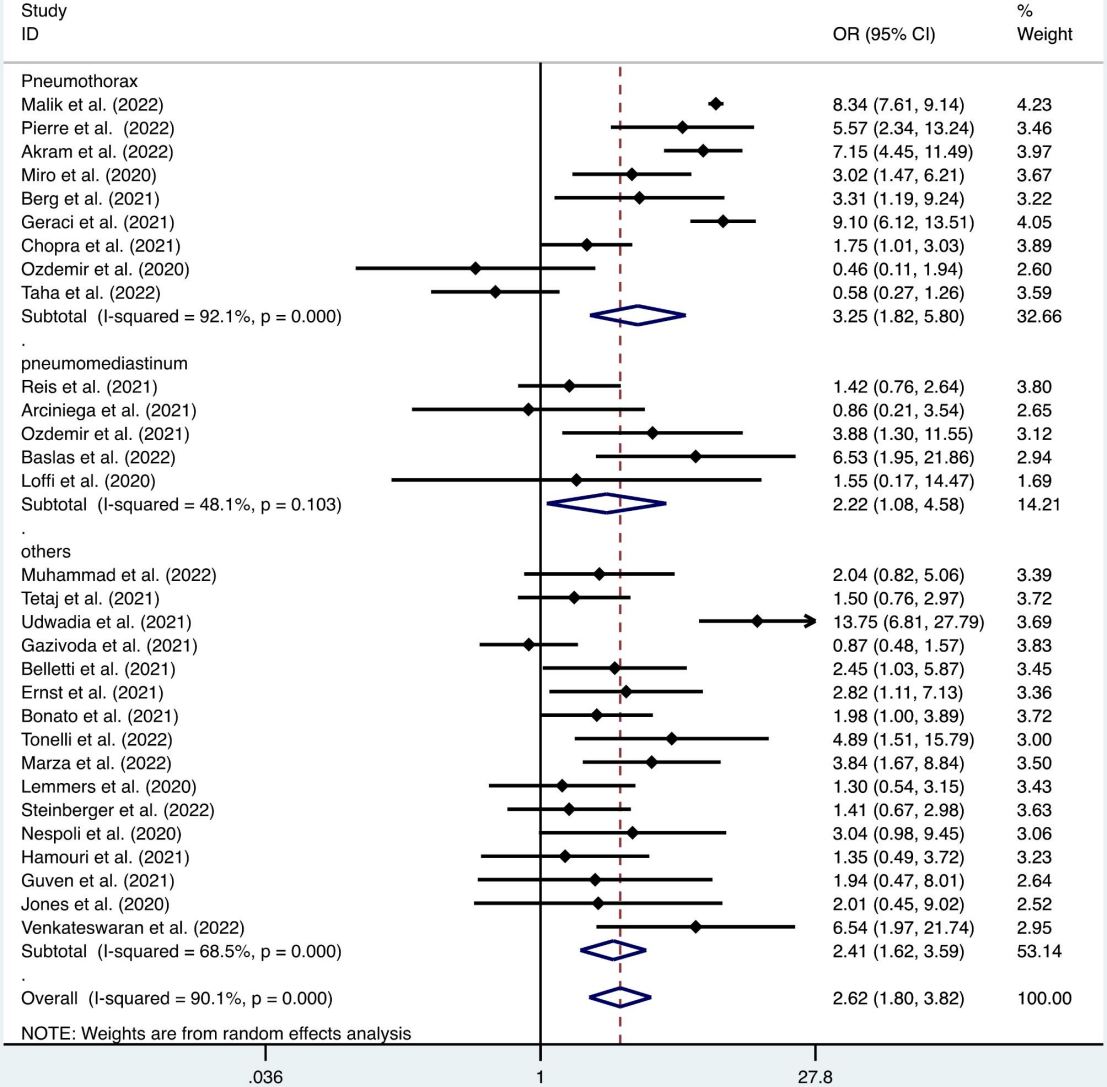
Forest plot of differences in the mortality rate between COVID-19 patients with and without pulmonary air leak (number of events: 30)

Our study showed that PAL contributed to an increased mortality rate in patients with COVID-19 (OR = 2.62, 95% CI: 1.80–3.82, I^2^ = 90.1%, p < 0.001, Fig. 3). Since mortality is a very important clinical index, we performed a subgroup analysis of the mortality rate in COVID-19 patients with different types of PAL and found that COVID-19 patients with pneumothorax had a higher mortality rate than those without pneumothorax (OR = 3.25, 95% CI: 1.82–5.80, I^2^ = 92.1%, p < 0.001, Fig. 3), and the same conclusion was obtained for COVID-19 patients with pneumomediastinum (OR = 2.22, 95% CI: 1.08–4.58, I^2^ = 48.1%, p = 0.031, Fig. 3). A subgroup analysis of the mortality rate in COVID-19 patients with subcutaneous emphysema was not performed because no data were available.

We also compared the mortality rate in patients with COVID-19 who had multiple types of PAL at the same time and those who had only one type (Additional file 3: Table S6). The results showed that there was no significant difference between the two groups (OR = 2.07, 95% CI: 0.69–6.21, I^2^ = 41.7%, p = 0.194, Additional file 3: Figure S28).

### Reporting biases

We used funnel plots and Egger’s test for the analysis of reporting bias. The results showed that there was no reporting bias in most of the conclusions (Additional file 2).

## Discussion

The incidence of PAL in patients with COVID-19 is much higher than that in patients without COVID-19, which may be due to various reasons. The primary target of SARS-CoV-2 is the angiotensin-converting enzyme-2 (ACE-2) receptor, which is more frequently expressed in type II pneumocytes. Previous studies have shown that ACE-2 has a protective effect against pulmonary inflammation, pulmonary fibrosis, and pulmonary hypertension. However, the interaction between ACE-2 and SARS-CoV-2 may lead to downregulation [49]. A host-triggered dysregulated immune response causes lung injury in COVID-19 patients, leading to extensive inflammation and eventual fibrosis, which may lead to dysregulated surfactant production, impaired lung compliance, and increased alveolar air space surface tension, thus predisposing individuals with COVID-19 to PAL [50]. COVID-19 also causes the development of nonhomogeneous pulmonary parenchyma, which can result in maldistribution of ventilatory stress due to different local stresses in two consecutive lung regions with different compliance and lead to PAL [7]. The increased probability of PAL in COVID-19 patients may also be related to the Macklin effect, in which the rupture of the alveolar tree is related to the increase in pressure in the alveoli, which may release air and subsequently dissect the peribronchial sheath [51]. These alveoli rupture, isolate or merge and then cause pulmonary lacerations. Belletti et al. reported that almost all patients with pneumothorax/pneumomediastinum showed the Macklin effect on chest CT [35]. Another reason for the high incidence of PAL in patients with COVID-19 may be coughing, a common symptom of COVID-19. The increased intrathoracic pressure caused by coughing can cause the alveolar walls to rupture, leading to air leakage from damaged alveoli and separating the interstitial tissue around the bronchus [52, 53].

Noppen et al. showed that before the pandemic, the incidence of pneumothorax was higher in men (7.4 to 18 cases per 100,000 people) than in women (1.2 to 6 cases per 100,000 people) [54]. In 2015, Kouritas et al. indicated that men account for approximately 76% of patients with pneumomediastinum [55]. We obtained a similar conclusion that male COVID-19 patients are more likely to develop PAL. Bwire et al. explained the biological differences between the male and female immune systems, noting that men have higher ACE-2 expression due to different sex hormones and are less resistant to SARS-CoV-2 infection than women [56]. Chen et al. believed that among COVID-19 patients, the conditions of men were often more serious [57], which might lead to more air leak events in male patients. Our study found that in COVID-19 patients, younger individuals were more likely to develop PAL, which is consistent with findings in non-COVID-19 patients [55, 58]. Diabetes and hypertension are the most common comorbidities in COVID-19 patients with PAL because the expression of ACE-2 receptors is increased in patients with these conditions [7, 59]. Our study showed that among COVID-19 patients, patients with PAL had a lower risk of developing diabetes and hypertension. This finding may be because the incidence of diabetes and hypertension is positively correlated with age, while COVID-19 patients develop PAL at a younger age.

Infection with SARS-CoV-2 results in an insufficient respiratory capacity, and patients often require prolonged MV [60]. Our study found that COVID-19 patients with PAL had higher rates of MV and higher PEEP values. These higher rates are mainly because pulmonary barotrauma is a potential complication of MV, and it occurs as a result of excessive volume of lung parenchyma, which can cause alveolar rupture, leading to pneumothorax, pneumomediastinum and subcutaneous emphysema [61]. Gidaro et al. conducted a case‒control study and divided COVID-19 patients into two groups according to the levels of PEEP used (≤ 10 cmH_2_O and > 10 cmH_2_O). The number of patients in the high-PEEP group who developed PAL was much higher than that in the low-PEEP group [62]. Although a high PEEP maintains oxygenation and prevents repetitive alveolar collapse, it also partially overexpands lungs with normal compliance and then increases the risk of barotrauma [63, 64].

The COVID-19 pandemic rapidly saturated health care services, especially in the early stage. Some medical institutions suspended the services of other departments and converted their wards into COVID-19 wards to cope with the rapid increase in the number of patients [65]. The challenge was equally acute in the ICU, with some patients with severe COVID-19 admitted to “nonconventional” temporary ICUs, such as operating theatres or postanaesthesia care units [66]. Our research showed that the occurrence of PAL in COVID-19 patients aggravated this tense situation. These patients needed more ICU, longer hospital stays and ICU stays and required more medical resources. Therefore, it is necessary to reduce the incidence of PAL in COVID-19 patients to relieve the enormous pressure placed on the health care system.

SARS-CoV-2 infection has caused millions of deaths worldwide [2], and severe PAL can also pose a great threat to patients’ lives [67]. Although the presence of multiple types of PAL did not increase the mortality risk among COVID-19 patients with PAL, the finding that PAL contributed to an increased mortality risk among COVID-19 patients is still of concern. The combined effects of COVID-19 and PAL may cause more damage to patients’ lungs, which leads to more severe illness and worse clinical outcomes.

### Limitations

Our study has certain limitations. The type of PAL was not completely consistent across the included studies, which might lead to overestimation or underestimation of events when studies were included in the pooled analysis. Publication bias and high heterogeneity existed in a small part of our study results, which can be solved by incorporating more high-quality studies in the future. Since November 2021, the Omicron variant has rapidly spread worldwide [68]. In this meta-analysis, all the patients included had been infected prior to the Omicron outbreak. The effect of PAL on patients infected with the Omicron variant requires further study.

## Conclusions

In this systematic review and meta-analysis, we studied and discussed the effects of PAL on patients with COVID-19 in detail. Patients with lung injuries caused by COVID-19 may develop PAL. COVID-19 patients with PAL require more medical resources, have more serious conditions and have worse clinical outcomes.

### Electronic supplementary material

Below is the link to the electronic supplementary material.

Supplementary Material 1

Supplementary Material 2

Supplementary Material 3

## Data Availability

All data relevant to the study are included in the article/supplementary material, further inquiries can be directed to the corresponding author.

## Abbreviations

ACE-2: Angiotensin-converting enzyme-2
ARDS: Acute respiratory distress syndrome
CI: Confidence intervals
COPD: Chronic obstructive pulmonary disease
COVID-19: Coronavirus disease 2019
CT: Computed tomography
ECMO: Extracorporeal membrane oxygenation
ICU: Intensive care unit
MERS: Middle East respiratory syndrome
MV: Mechanical ventilation
OR: Odds ratio
PAL: Pulmonary air leak
PEEP: Positive end-expiratory pressure
PRISMA: Preferred reporting items for systematic reviews and meta-analyses
SARS: Severe acute respiratory syndrome
SMD: Standardized mean difference
