# Supplementary material for: Effects of pulmonary air leak on patients with coronavirus disease 2019 (COVID-19): a systematic review and meta-analysis
Source: BMC Pulm Med. 2023 Oct 19;23:398. doi: 10.1186/s12890-023-02710-2 (PMC10588255; doi:10.1186/s12890-023-02710-2)
Supplement: Supplementary file 1 — Supplementary Material 1 [file 12890_2023_2710_MOESM1_ESM.docx]

**Search strategy**

Search strategy in PubMed: **((((((air leak[Title/Abstract]) OR (barotrauma[Title/Abstract])) OR (barotraumas[Title/Abstract])) OR (pneumothorax[Title/Abstract])) OR (pneumomediastinum[Title/Abstract])) OR (subcutaneous emphysema[Title/Abstract])) AND (((((((COVID-19[Title]) OR (COVID-19 Virus Disease[Title])) OR (2019-nCoV[Title])) OR (Coronavirus Disease-19[Title])) OR (2019 Novel Coronavirus Disease[Title])) OR (SARS-CoV-2[Title])) OR (Coronavirus Disease 2019[Title]))**
