# Supplementary material for: Effects of pulmonary air leak on patients with coronavirus disease 2019 (COVID-19): a systematic review and meta-analysis
Source: BMC Pulm Med. 2023 Oct 19;23:398. doi: 10.1186/s12890-023-02710-2 (PMC10588255; doi:10.1186/s12890-023-02710-2)
Supplement: Supplementary file 2 — Supplementary Material 2 [file 12890_2023_2710_MOESM2_ESM.docx]

**Funnel plots and Egger’s publication bias plots.**
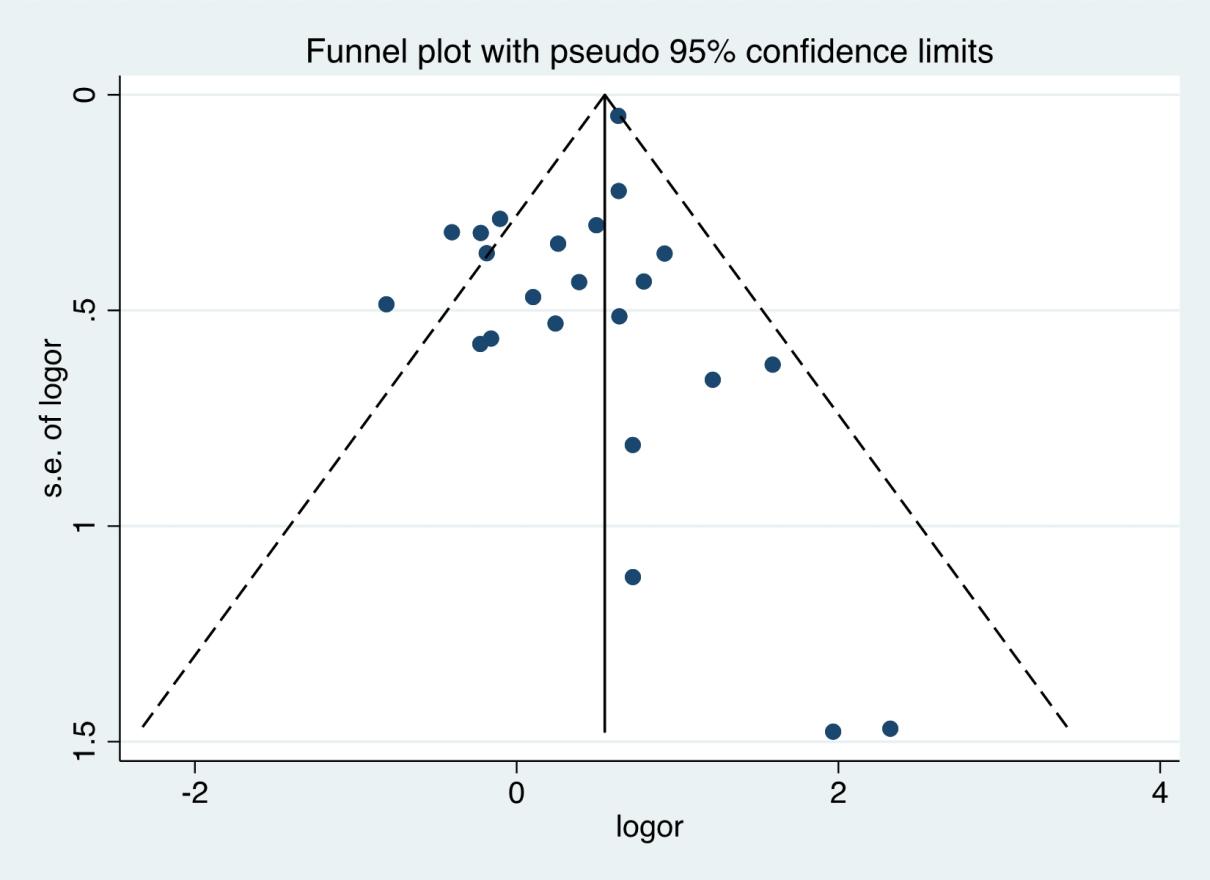


Funnel plot of differences in sex between COVID-19 patients with and without

pulmonary air leak (number of events: 23).


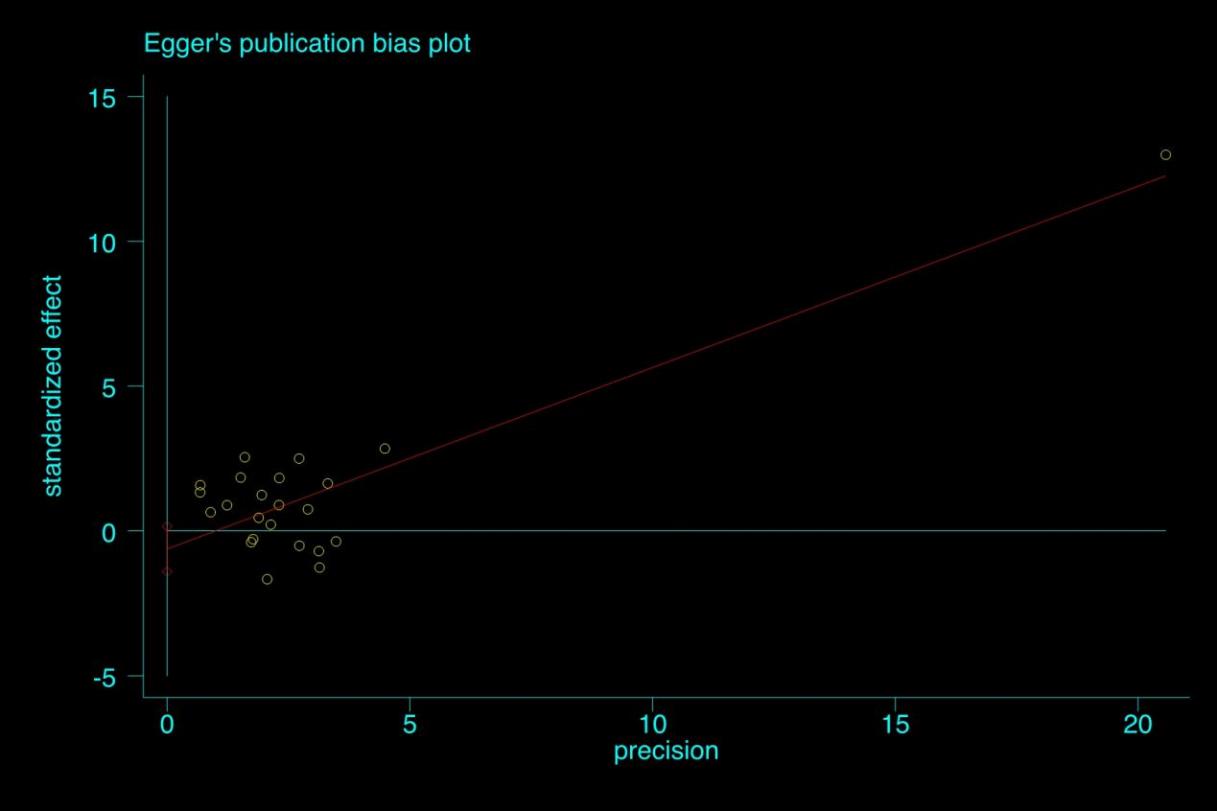


Egger’s publication bias plot of differences in sex between COVID-19 patients with and without pulmonary air leak (p=0.113, number of events: 23).


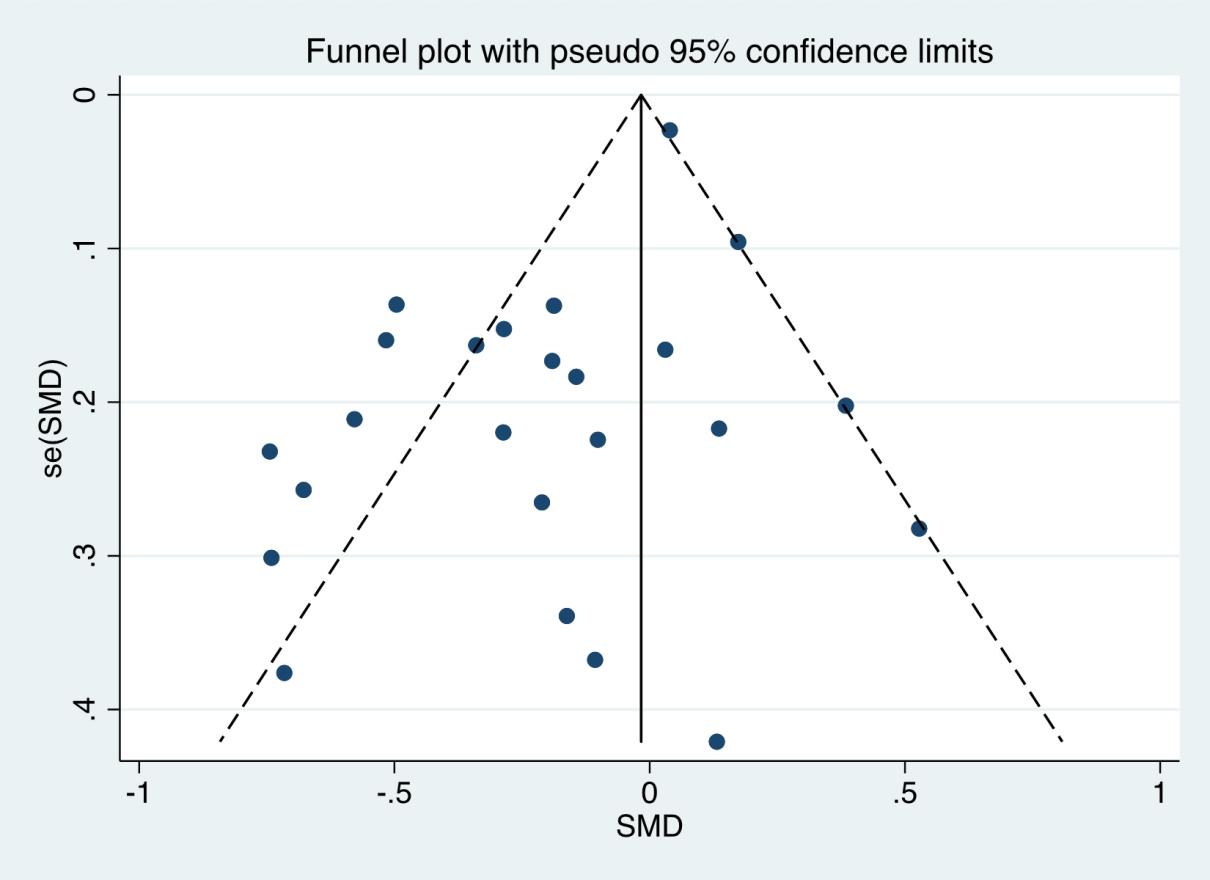


Funnel plot of differences in age between COVID-19 patients with and without

pulmonary air leak (number of events: 24).


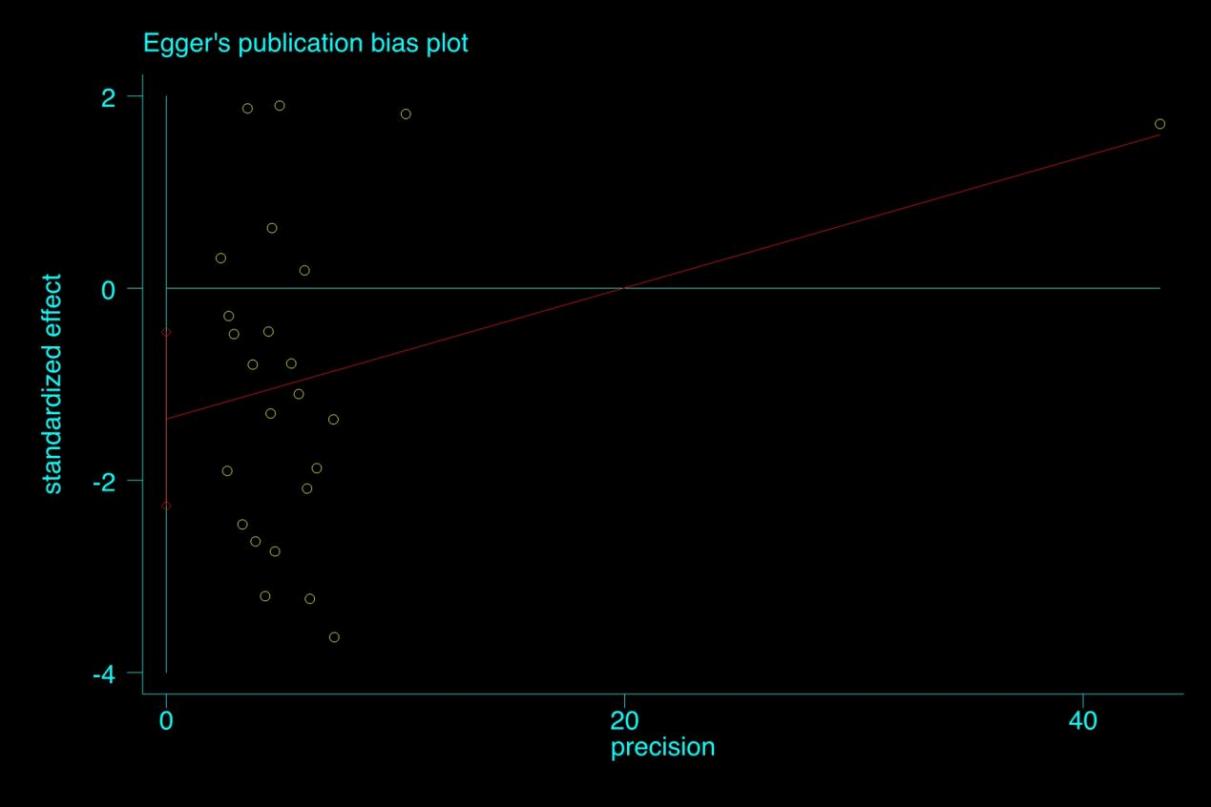


Egger’s publication bias plot of differences in age between COVID-19 patients with and without pulmonary air leak (p=0.005, number of events: 24).


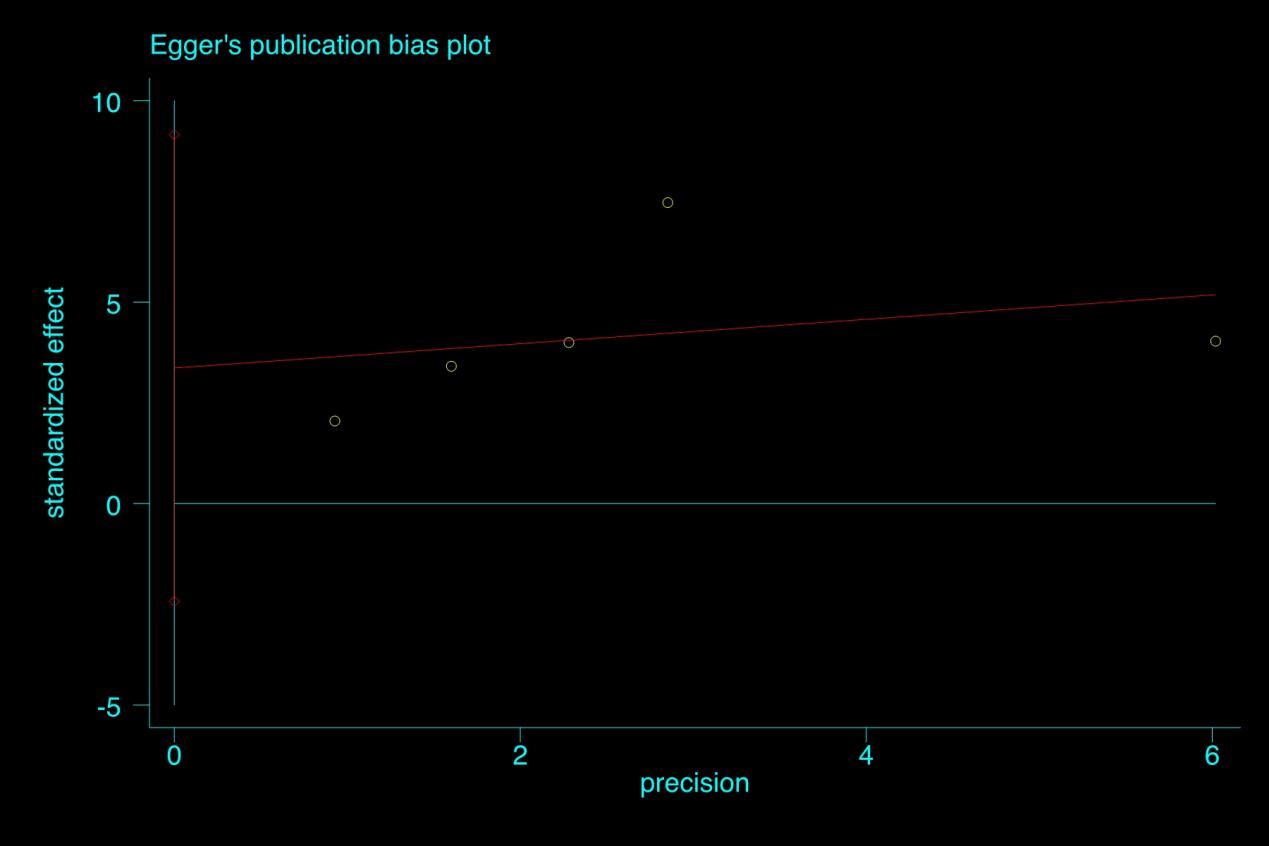


Egger’s publication bias plot of the incidence of pulmonary air leak (p=0.075, number of events: 5).


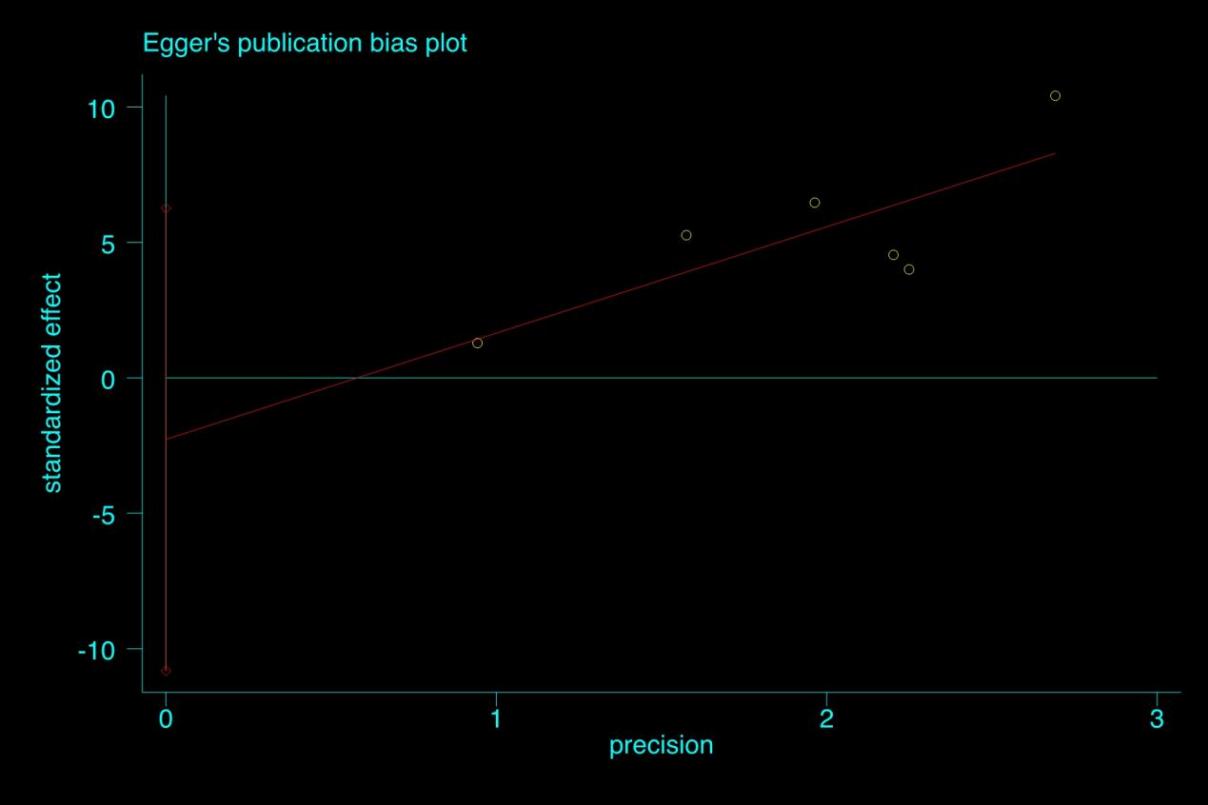


Egger’s publication bias plot of differences in [intensive care unit](javascript:;) admission between COVID-19 patients with and without pulmonary air leak (p=0.501, number of events: 6).


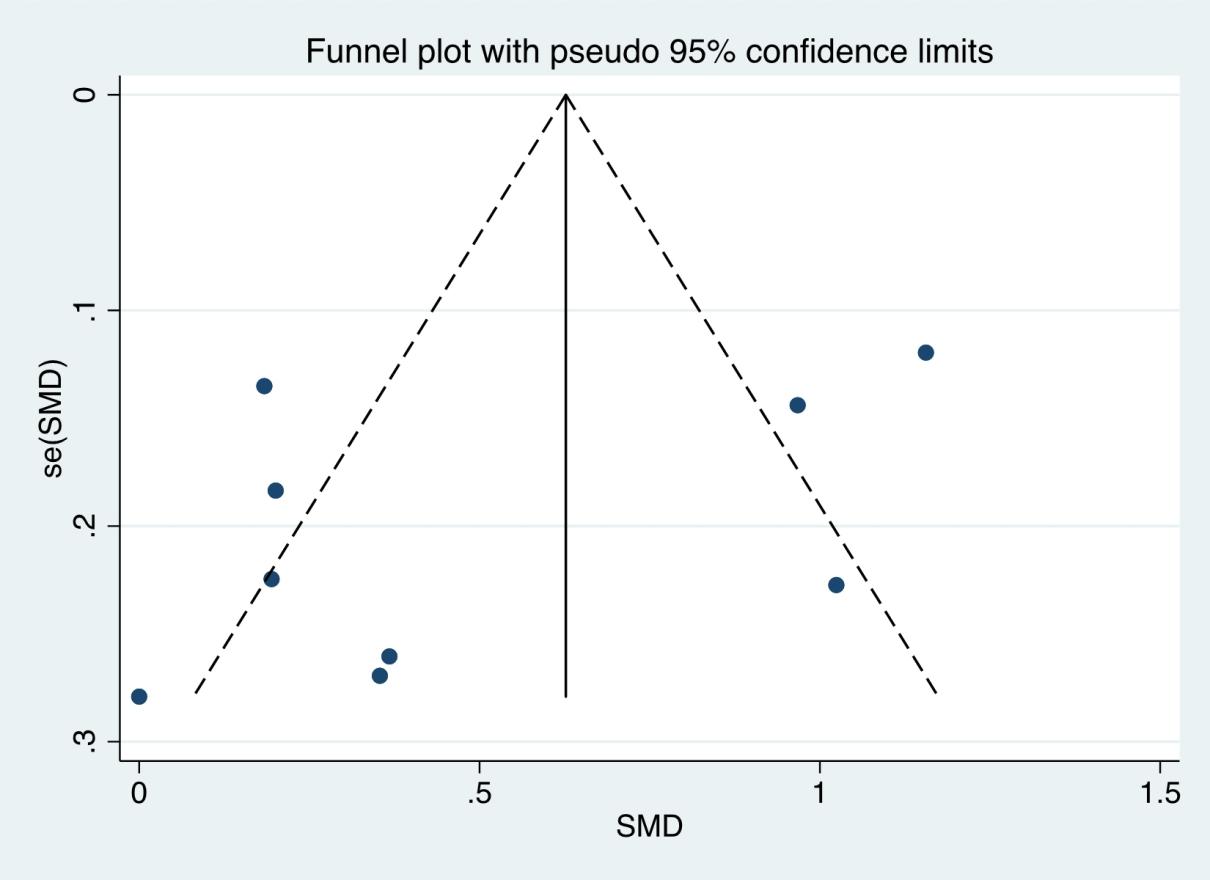


Funnel plot of differences in [intensive care unit](javascript:;) stay between COVID-19 patients with and without pulmonary air leak (number of events: 10).


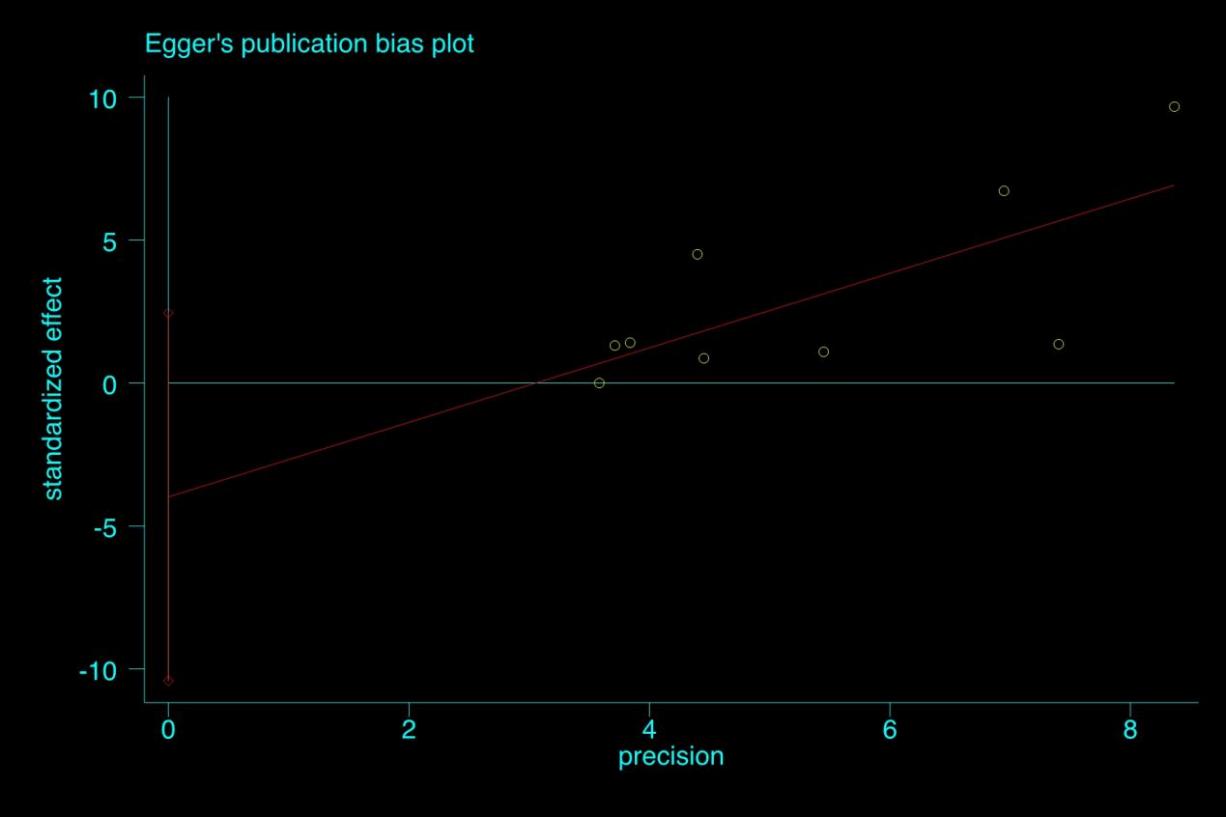


Egger’s publication bias plot of differences in [intensive care unit](javascript:;) stay between COVID-19 patients with and without pulmonary air leak (p=0.187, number of events: 10).


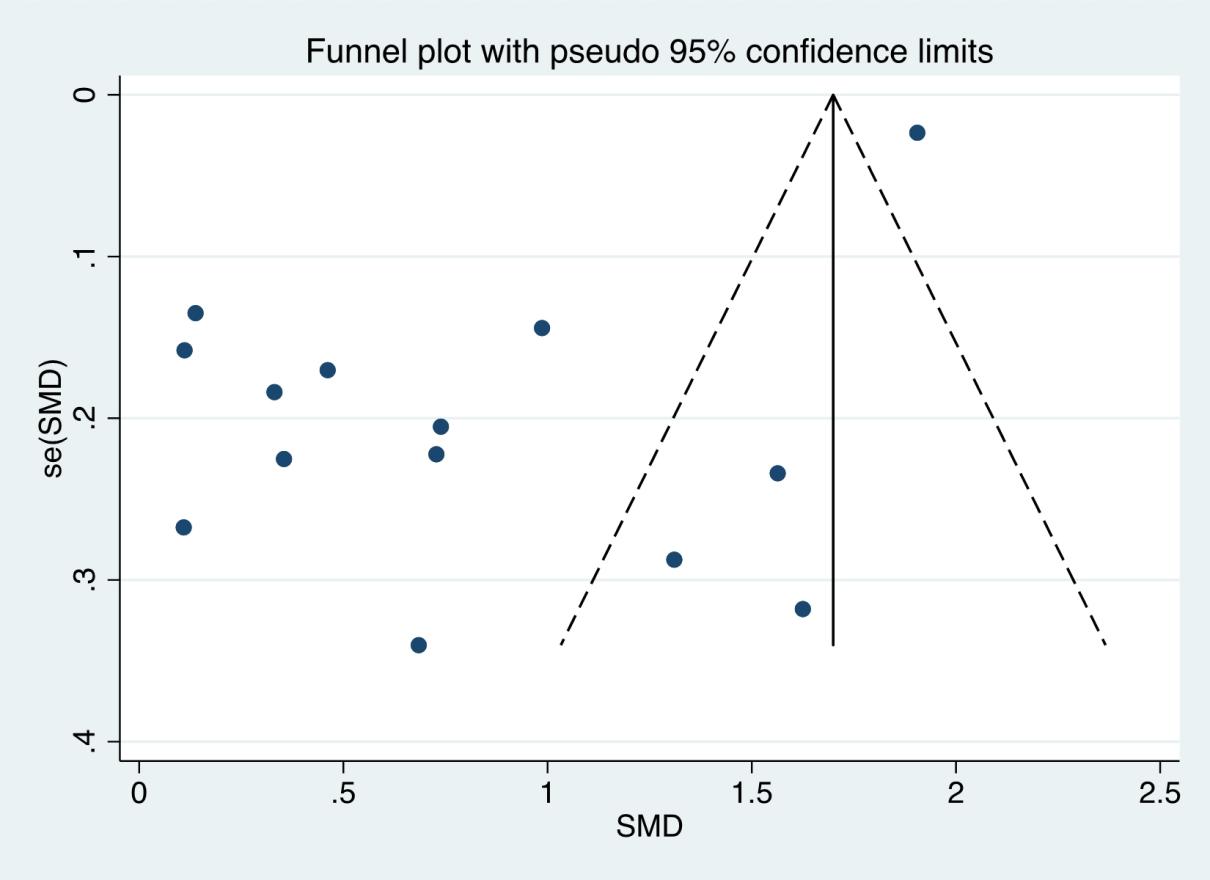


Funnel plot of differences in hospital stay between COVID-19 patients with and without pulmonary air leak (number of events: 14).


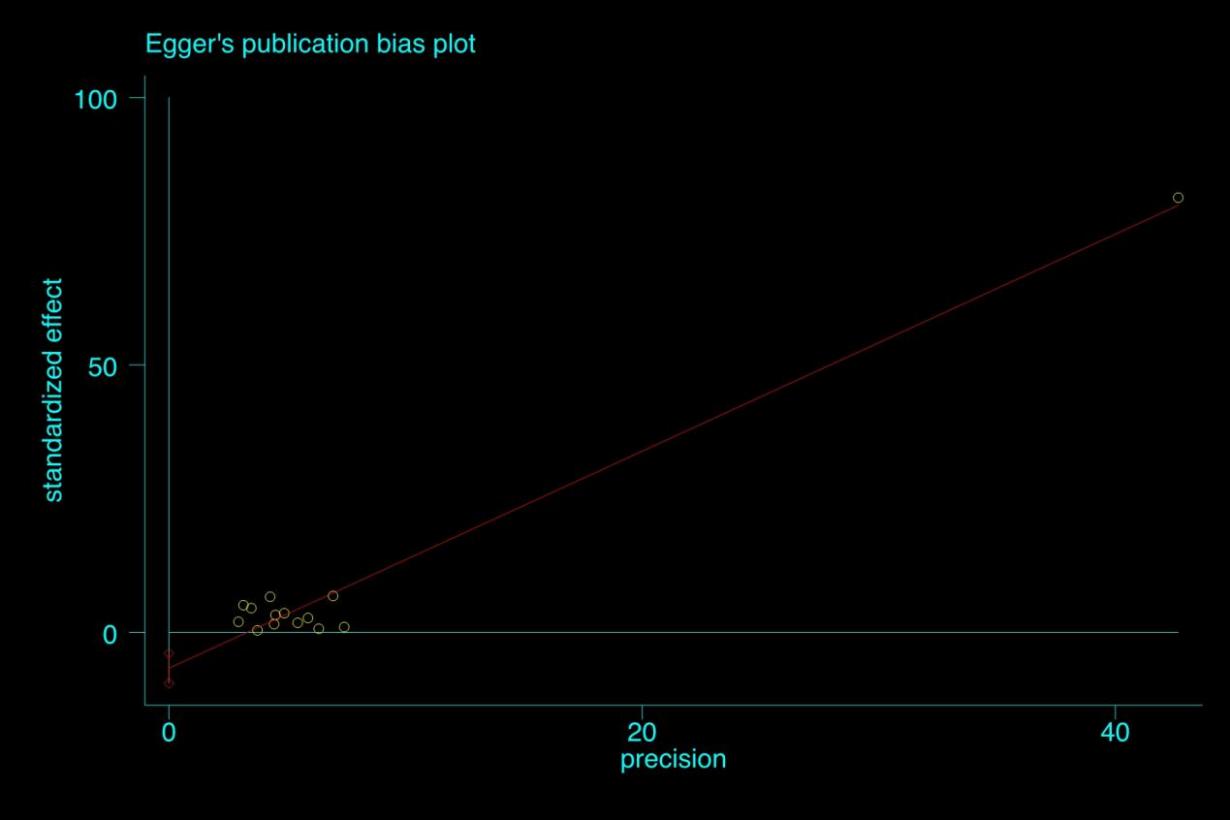


Egger’s publication bias plot of differences in hospital stay between COVID-19 patients with and without pulmonary air leak (p<0.001, number of events: 14).


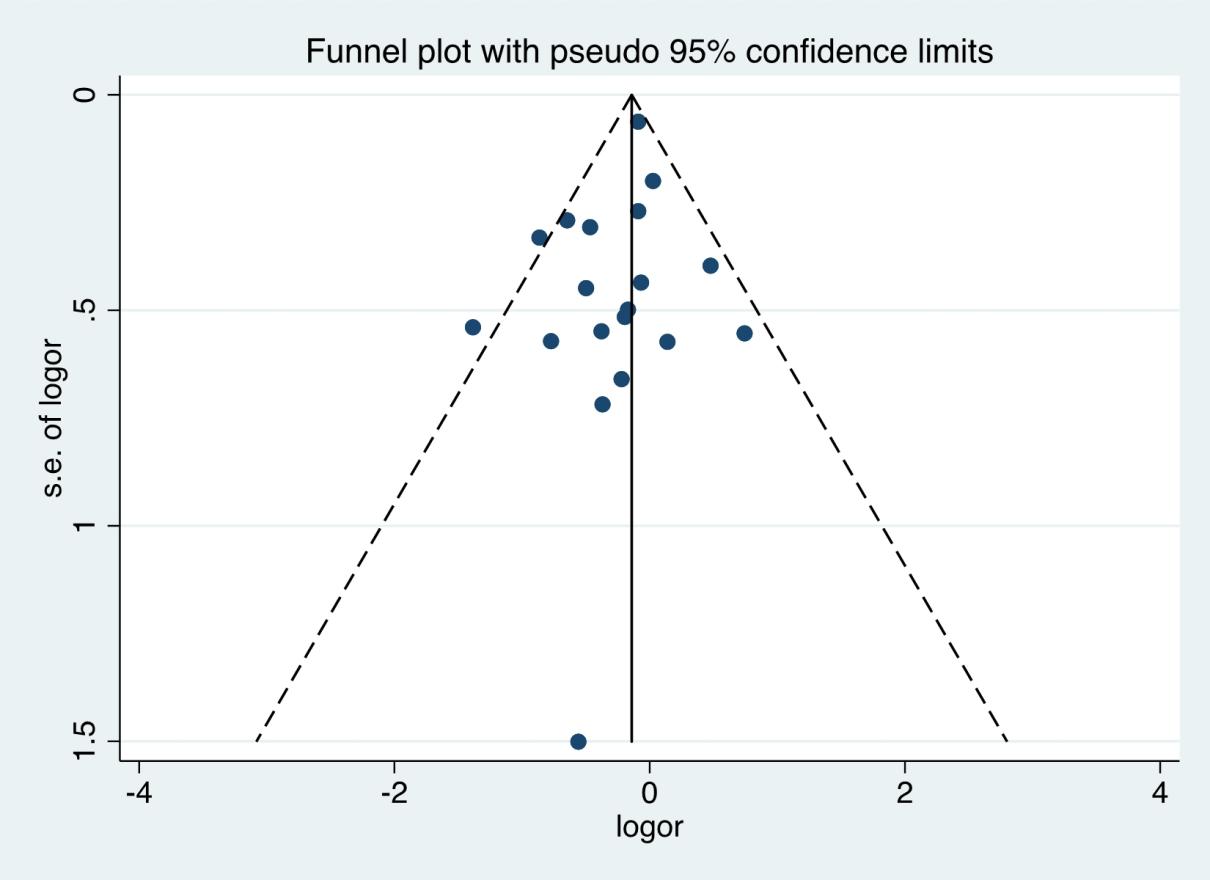


Funnel plot of differences in diabetes between COVID-19 patients with and without pulmonary air leak (number of events: 19).


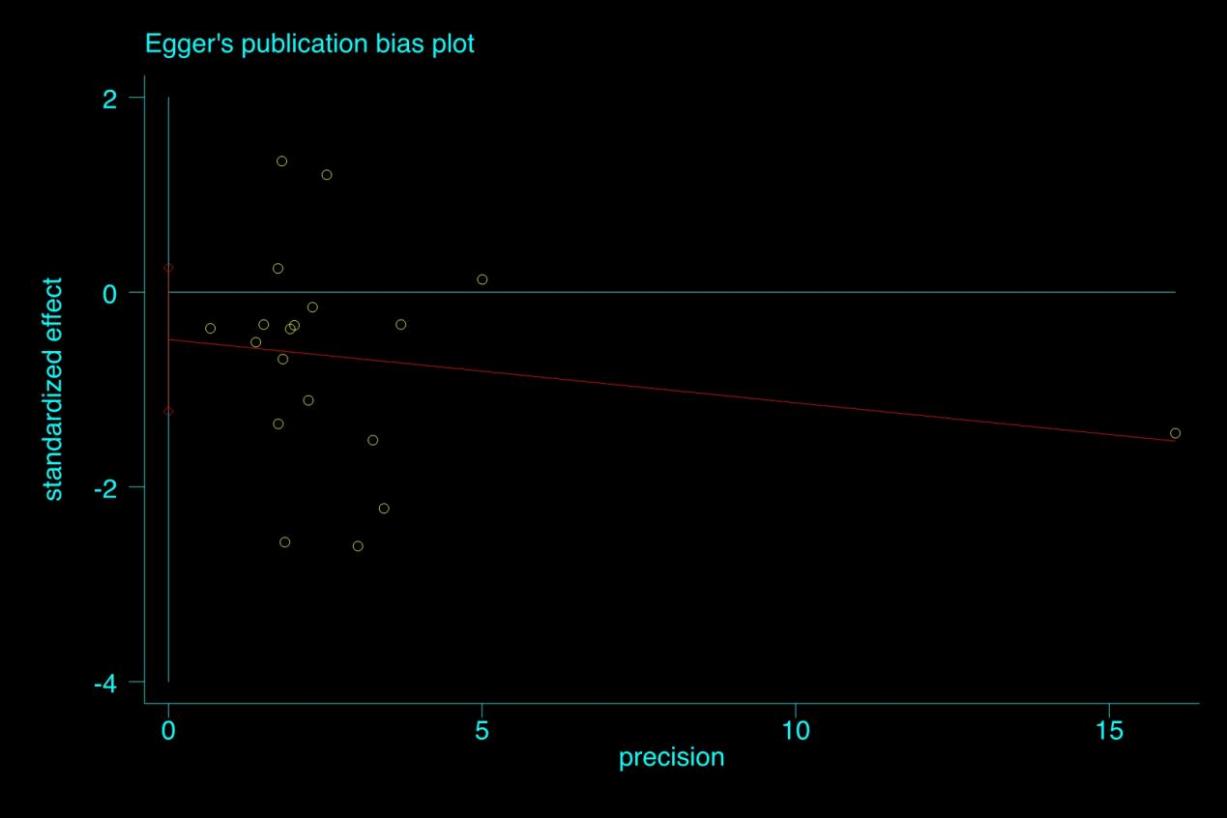


Egger’s publication bias plot of differences in diabetes between COVID-19 patients with and without pulmonary air leak (p=0.181, number of events: 19).


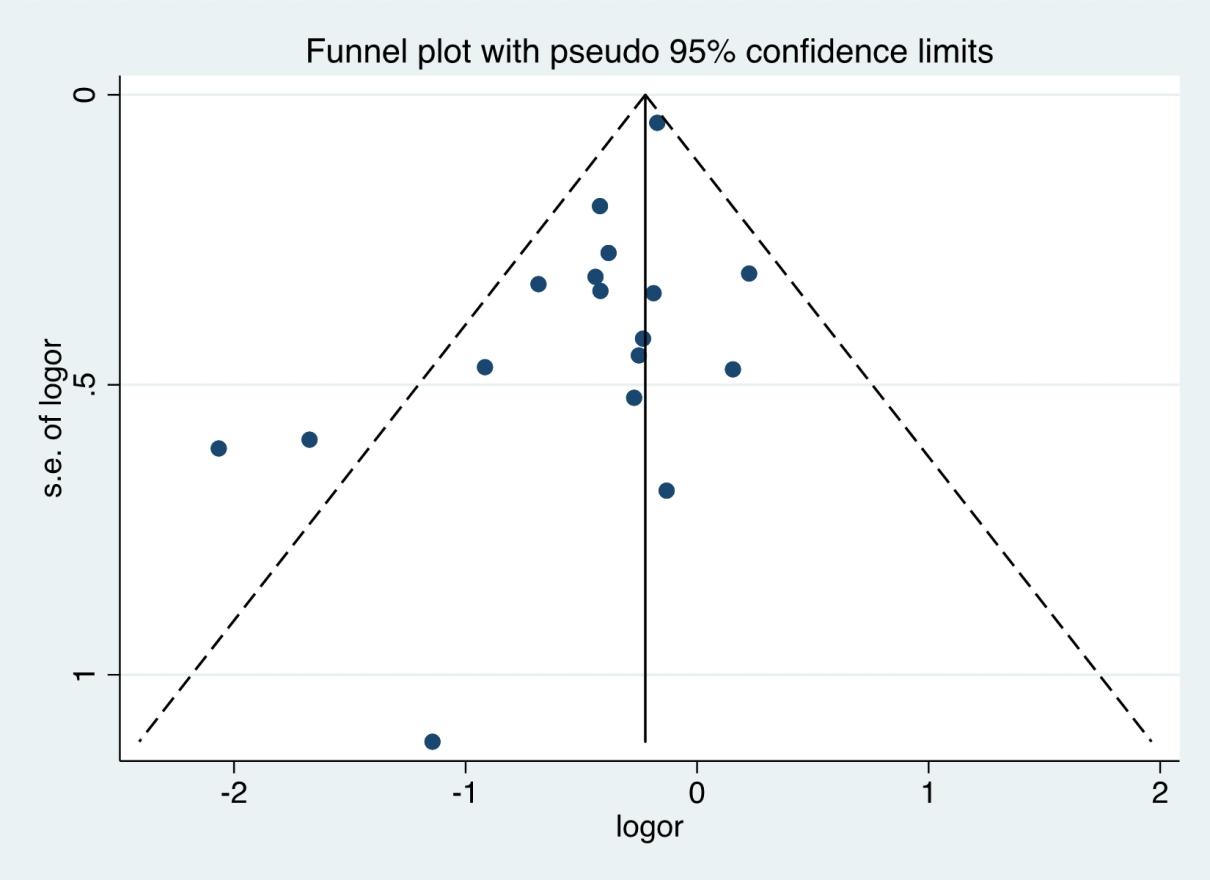


Funnel plot of differences in hypertension between COVID-19 patients with and without pulmonary air leak (number of events: 17).


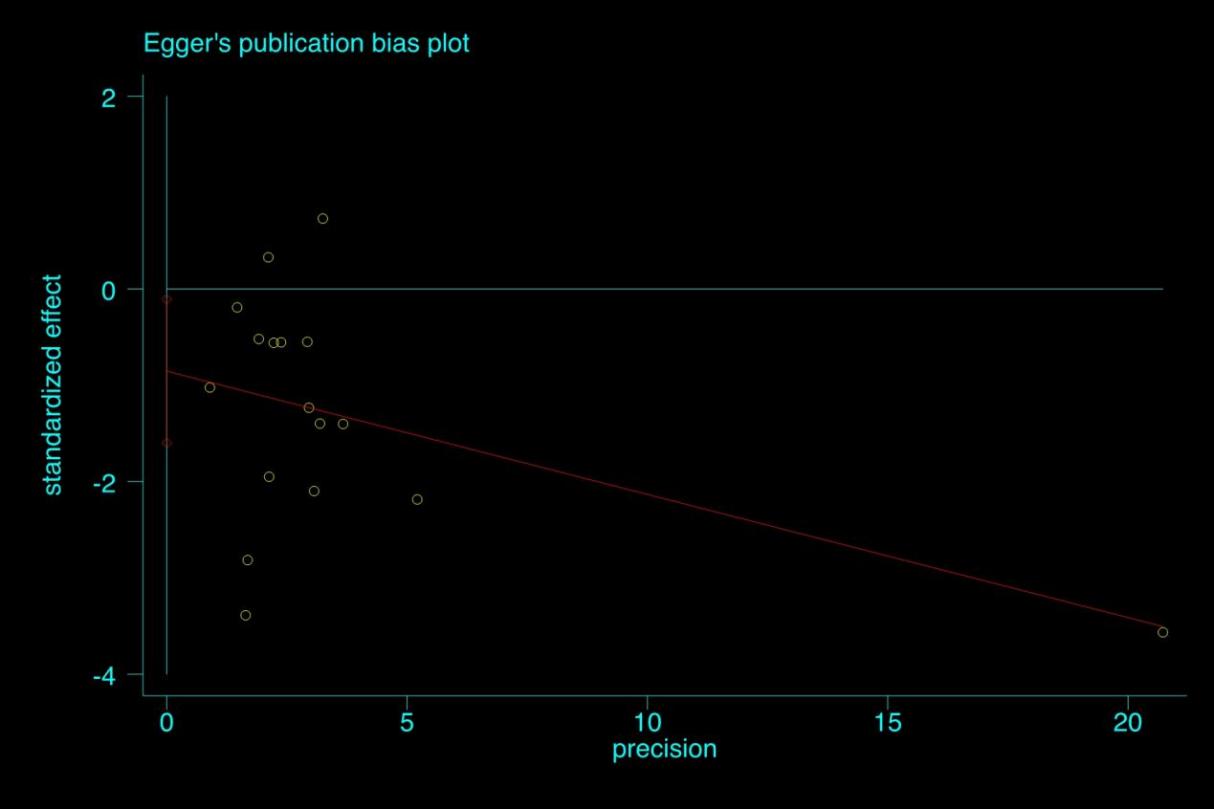


Egger’s publication bias plot of differences in hypertension between COVID-19 patients with and without pulmonary air leak (p=0.028, number of events: 17).


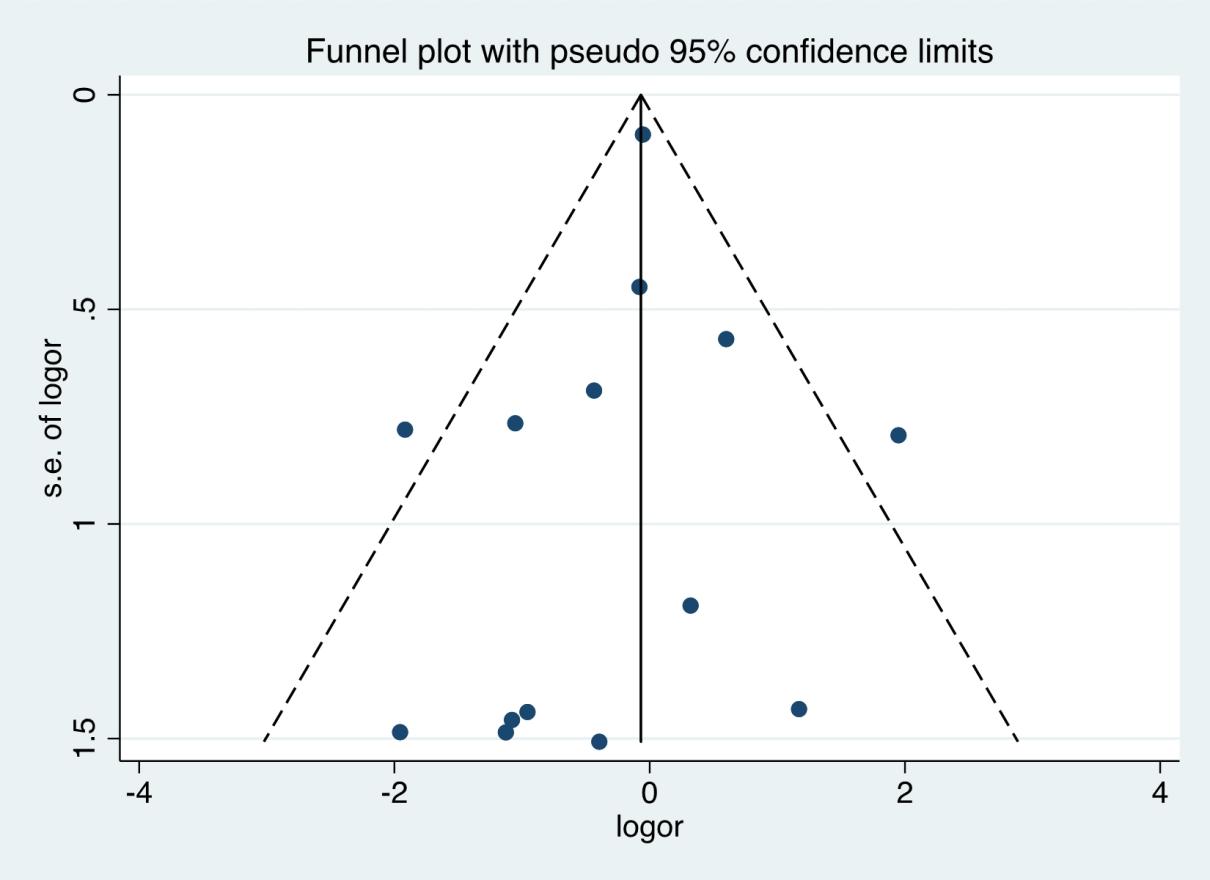


Funnel plot of differences in chronic obstructive pulmonary disease between COVID-19 patients with and without pulmonary air leak (number of events: 14).


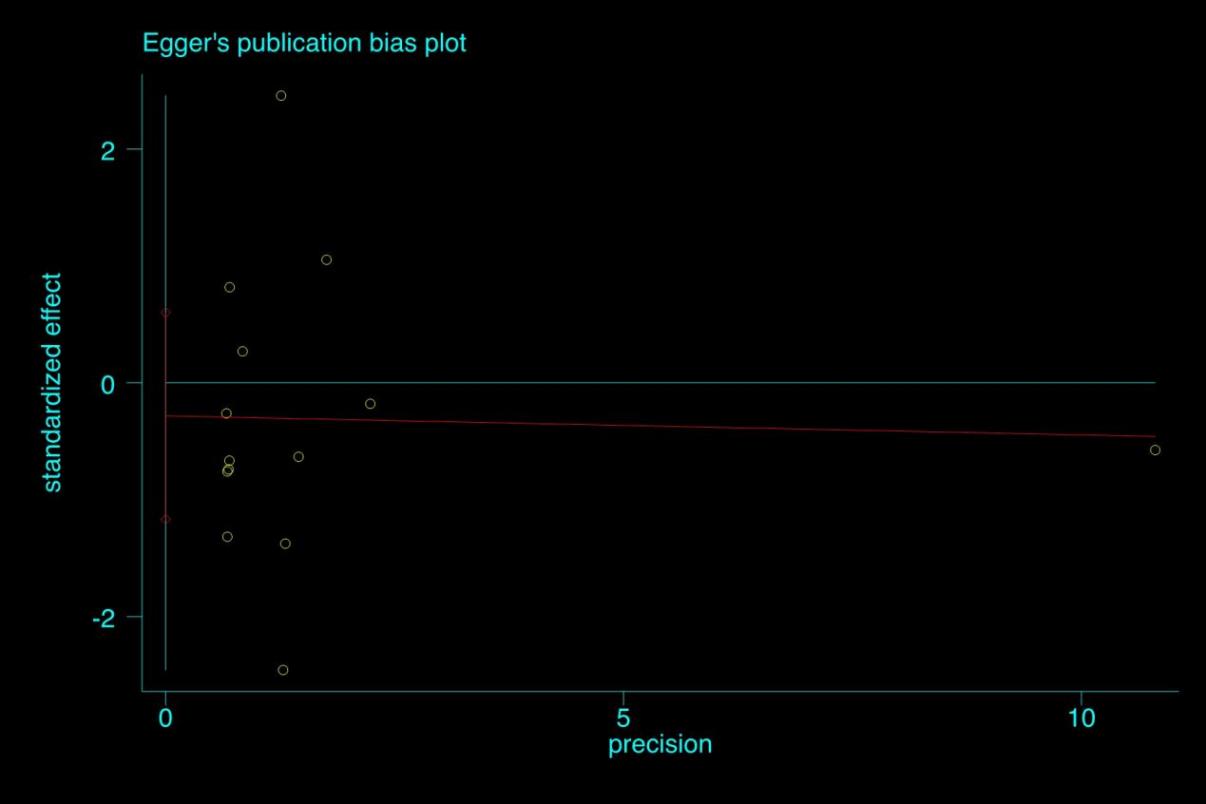


Egger’s publication bias plot of differences in chronic obstructive pulmonary disease between COVID-19 patients with and without pulmonary air leak (p=0.499, number of events: 14)


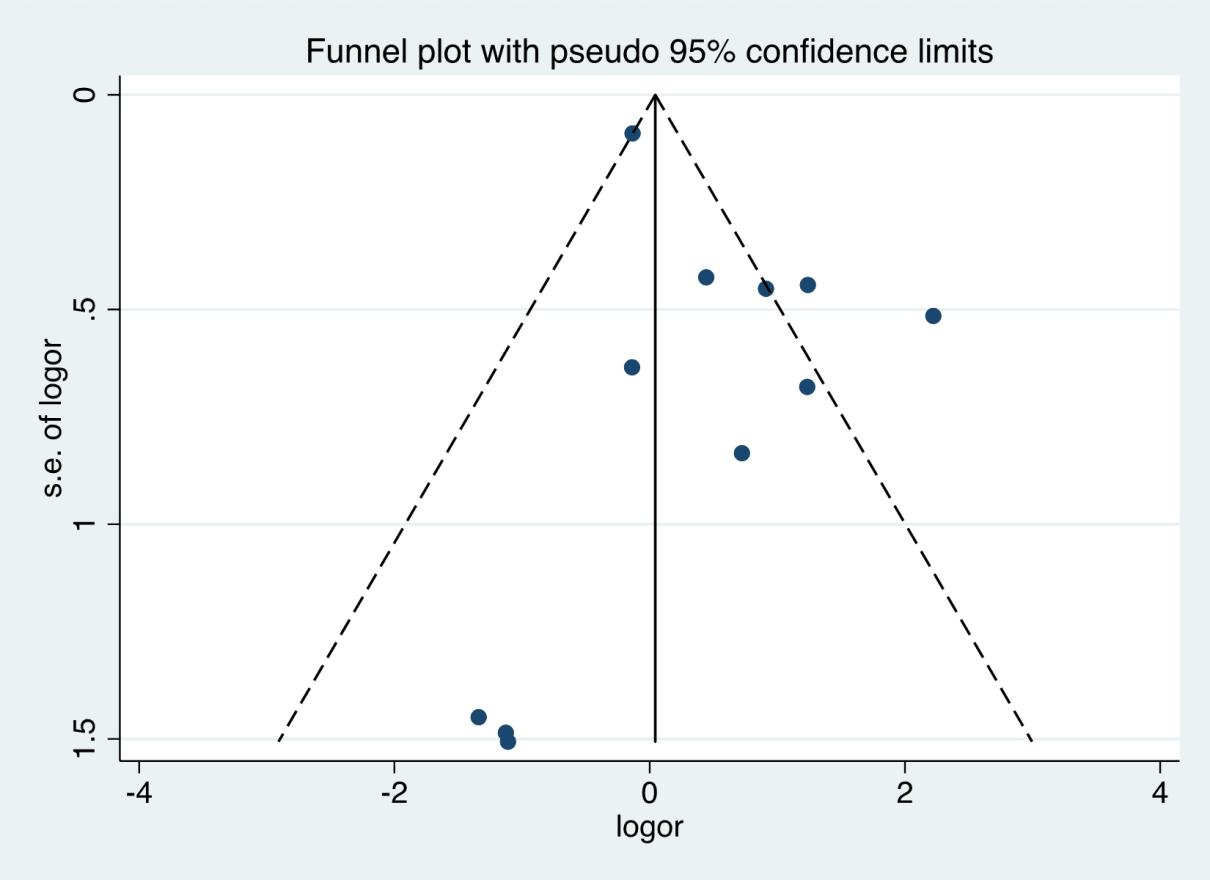


Funnel plot of differences in asthma between COVID-19 patients with and without pulmonary air leak (number of events: 11).


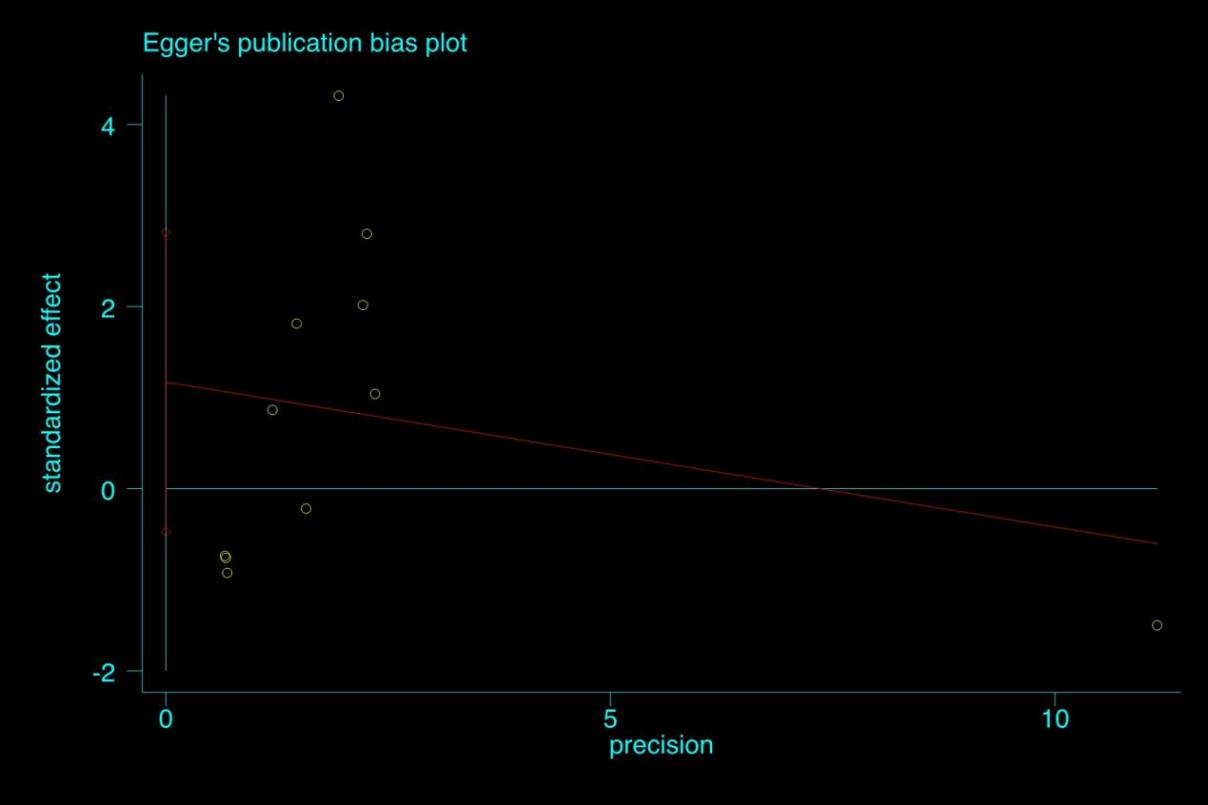


Egger’s publication bias plot of differences in asthma between COVID-19 patients with and without pulmonary air leak (p=0.142, number of events: 11).

.
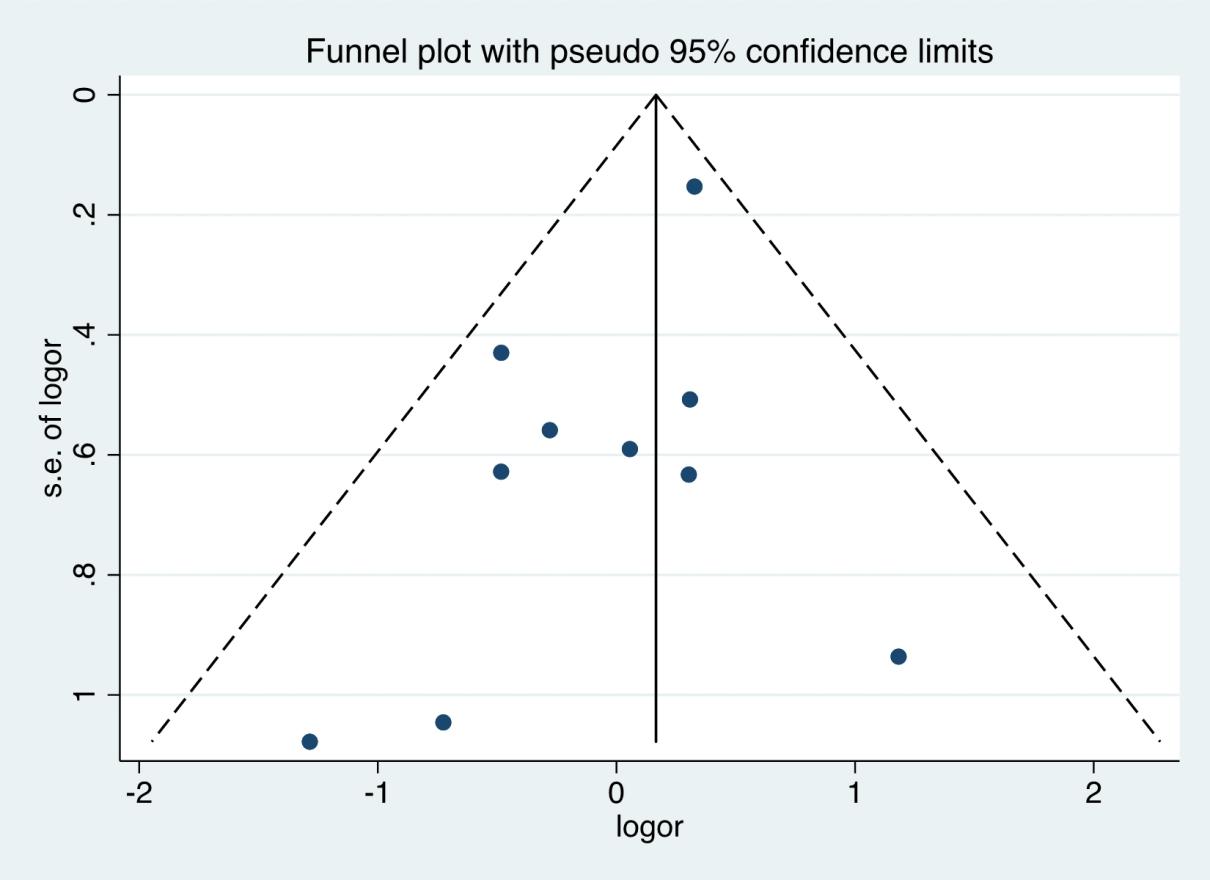


Funnel plot of differences in cancer between COVID-19 patients with and without pulmonary air leak (number of events: 10).


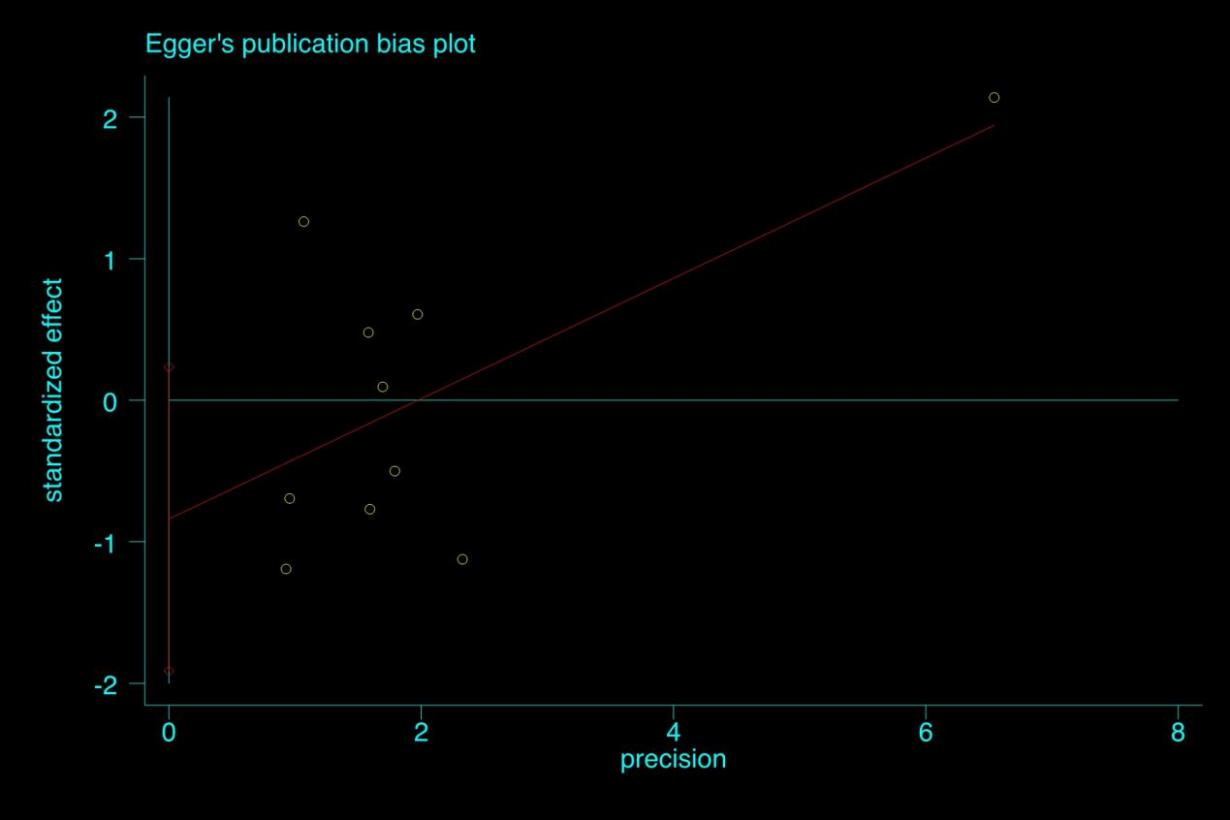


Egger’s publication bias plot of differences in cancer between COVID-19 patients with and without pulmonary air leak (p=0.109, number of events: 10).


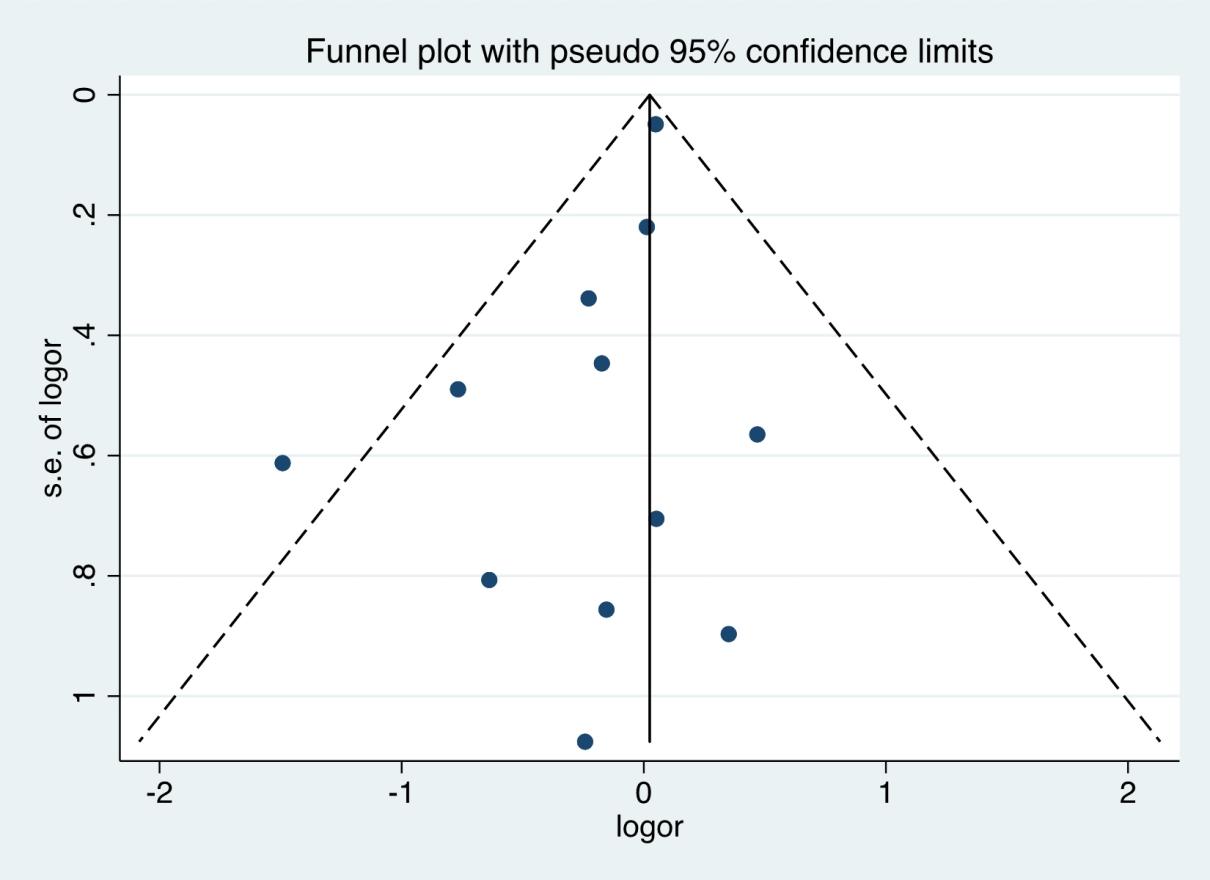


Funnel plot of differences in smoking between COVID-19 patients with and without pulmonary air leak (number of events: 12).


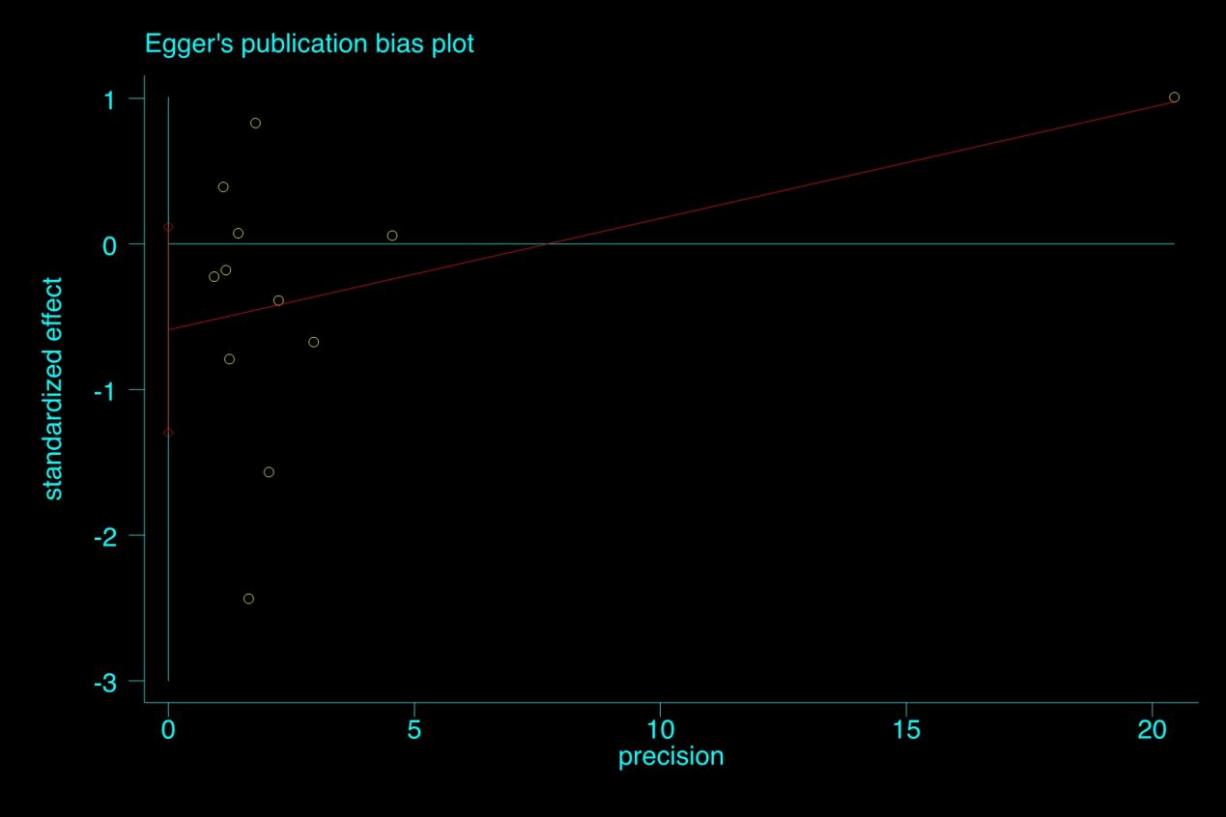


Egger’s publication bias plot of differences in smoking between COVID-19 patients with and without pulmonary air leak (p=0.093, number of events: 12).


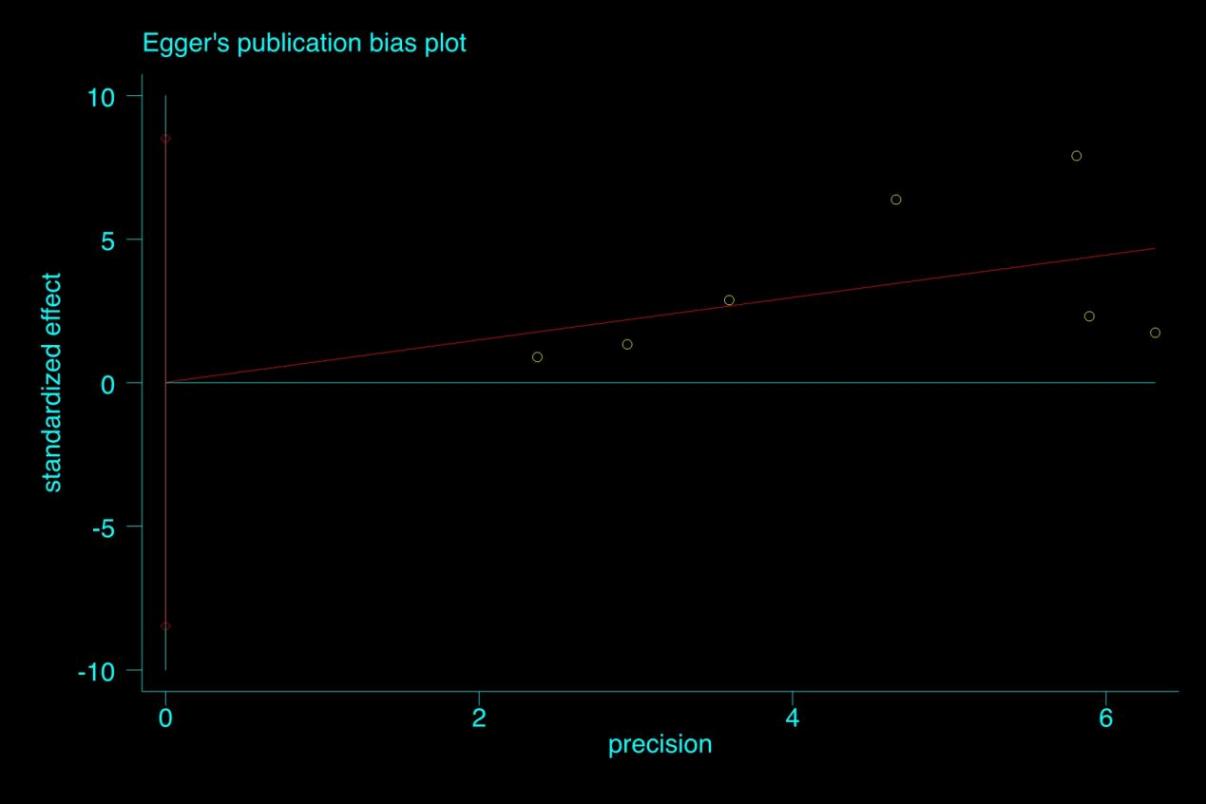


Egger’s publication bias plot of differences in D-dimer levels between COVID-19 patients with and without pulmonary air leak (p=0.996, number of events: 7).


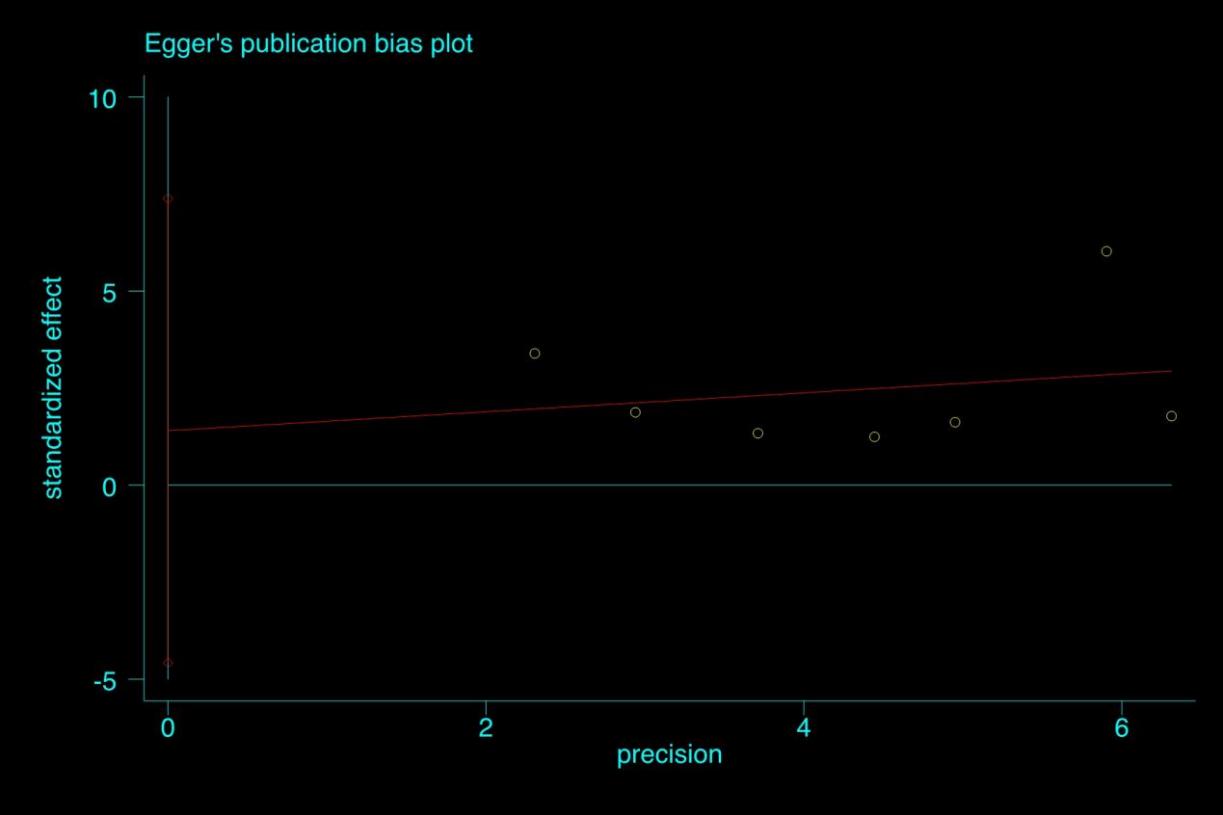


Egger’s publication bias plot of differences in leucocyte counts between COVID-19 patients with and without pulmonary air leak (p=0.573, number of events: 7).


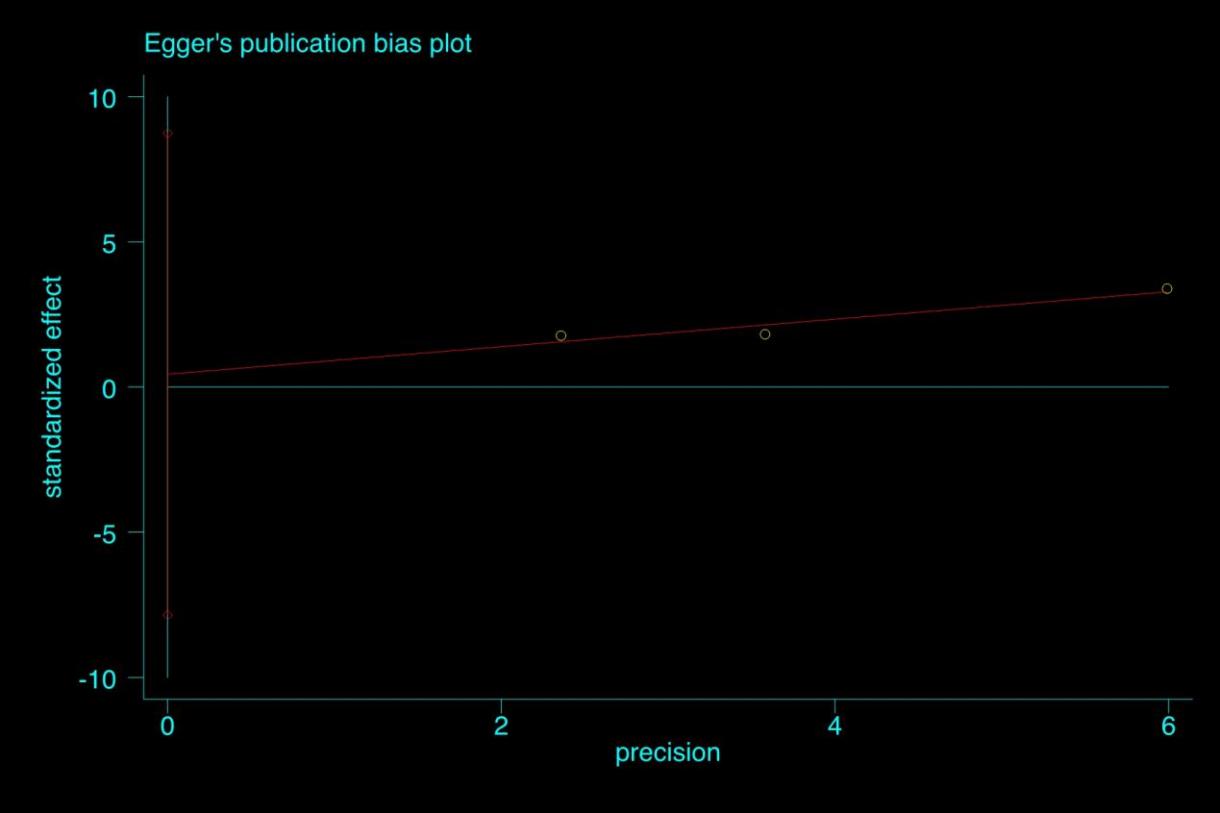


Egger’s publication bias plot of differences in aspartate aminotransferase levels between COVID-19 patients with and without pulmonary air leak (p=0.620, number of events: 3).


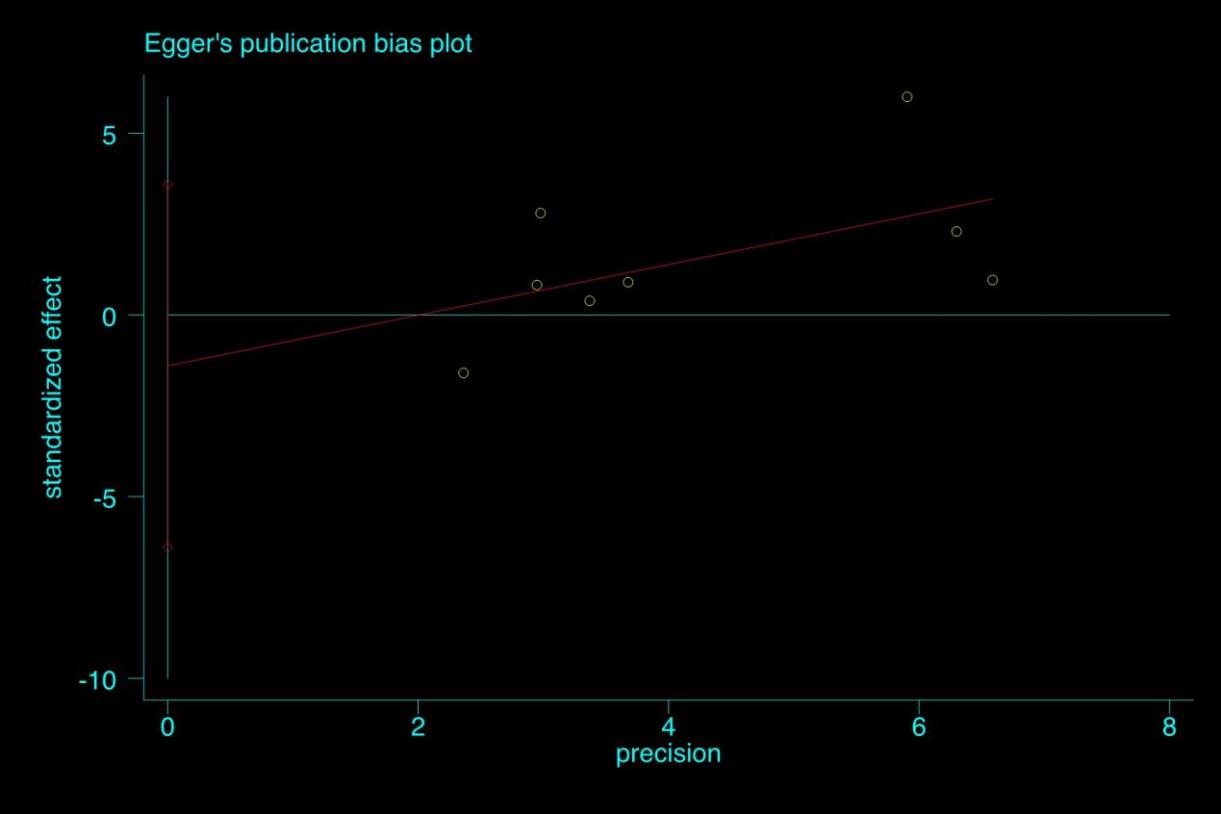


Egger’s publication bias plot of differences in lactate dehydrogenase levels between COVID-19 patients with and without pulmonary air leak (p=0.518, number of events: 8).


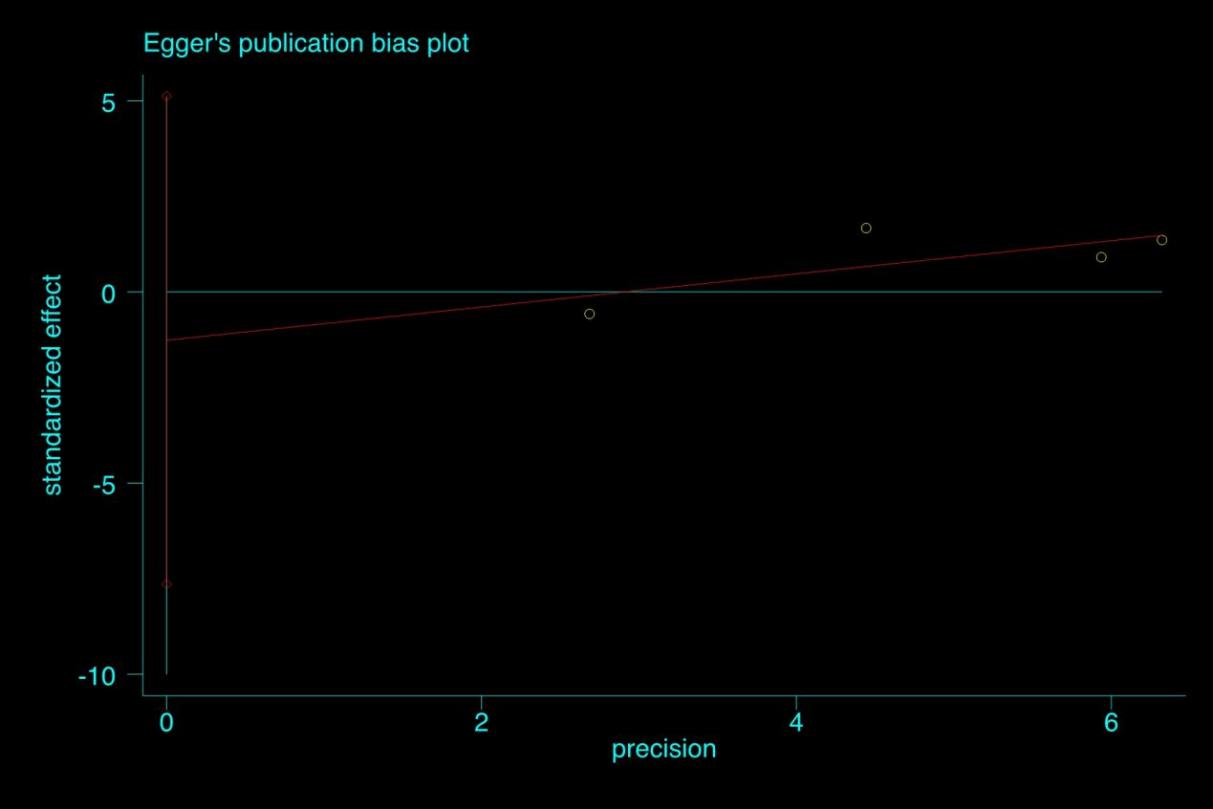


Egger’s publication bias plot of differences in neutrophil counts between COVID-19 patients with and without pulmonary air leak (p=0.484, number of events: 4).


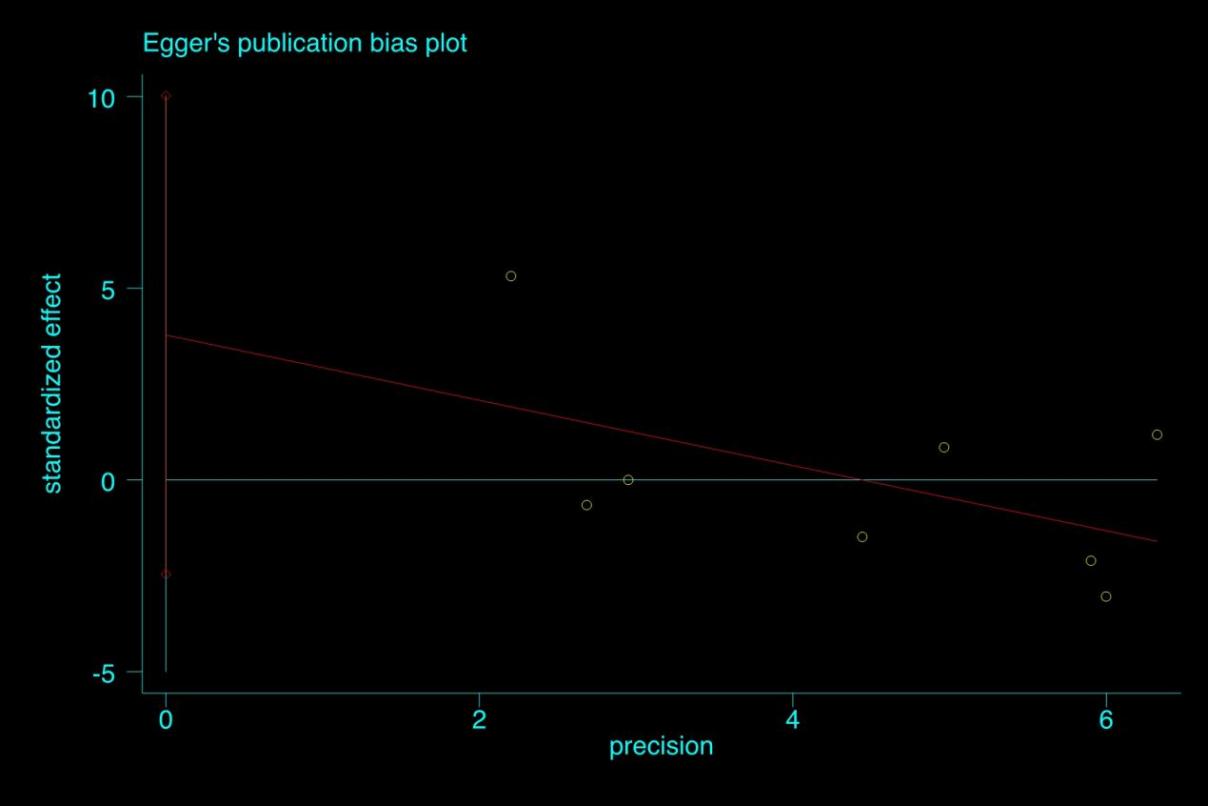


Egger’s publication bias plot of differences in lymphocyte counts between COVID-19 patients with and without pulmonary air leak (p=0.169, number of events: 8).


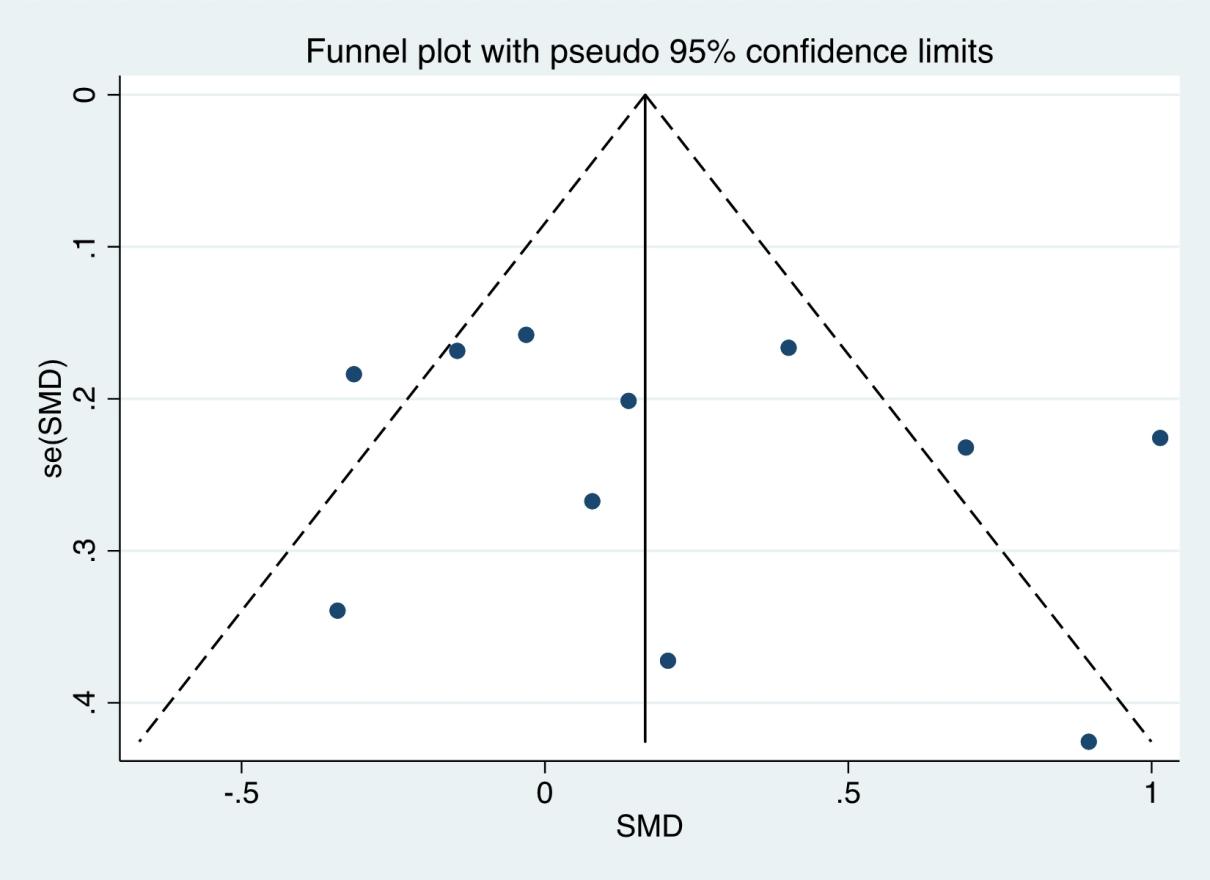


Funnel plot of differences in C-reactive protein levels between COVID-19 patients with and without pulmonary air leak (number of events: 11).


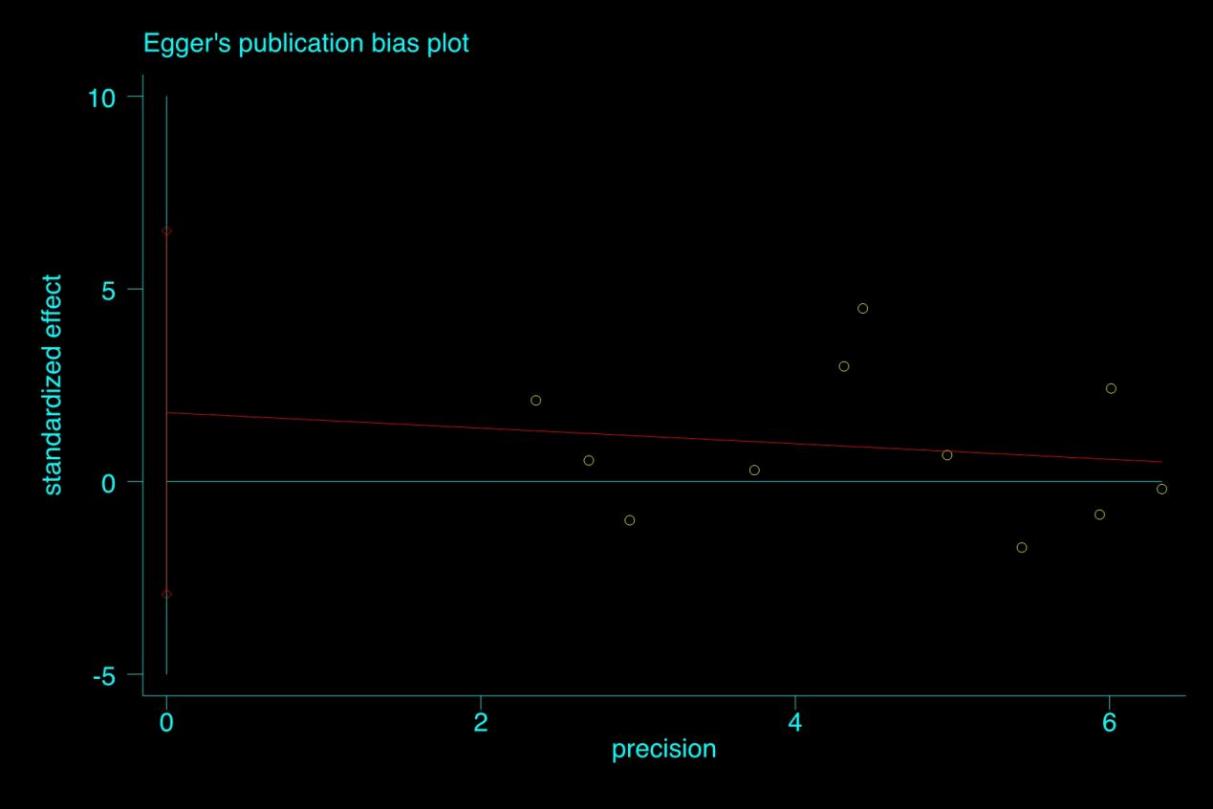


Egger’s publication bias plot of differences in C-reactive protein levels between COVID-19 patients with and without pulmonary air leak (p=0.413, number of events: 11).


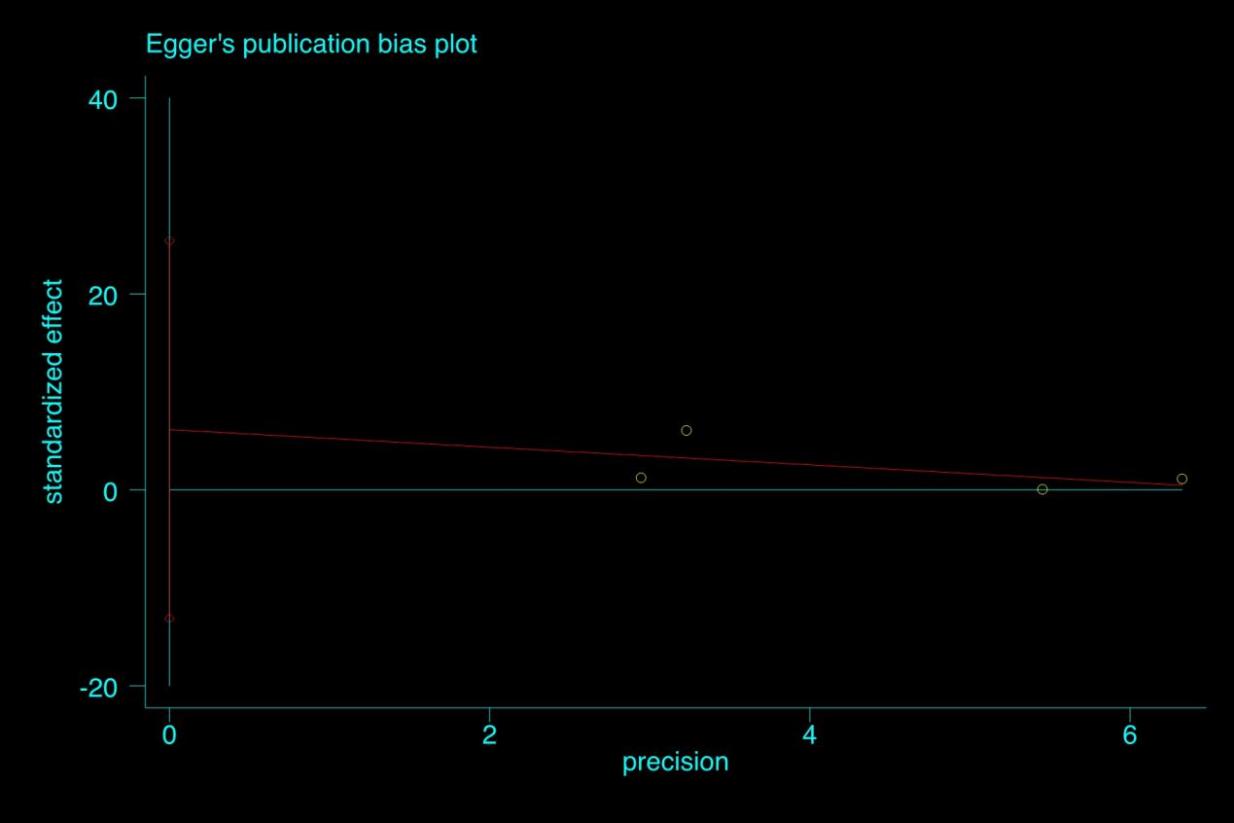


Egger’s publication bias plot of differences in ferritin levels between COVID-19 patients with and without pulmonary air leak (p=0.304, number of events: 4).


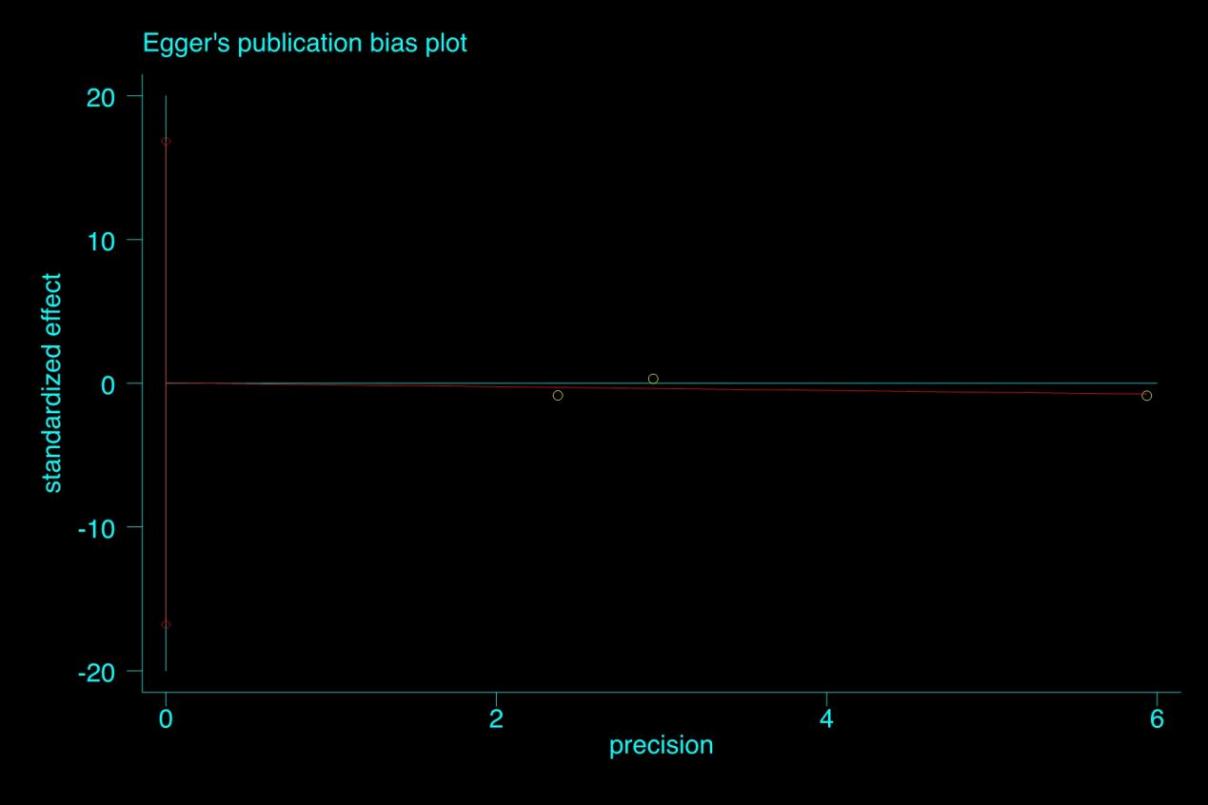


Egger’s publication bias plot of differences in platelet counts between COVID-19 patients with and without pulmonary air leak (p=0.986, number of events: 3).


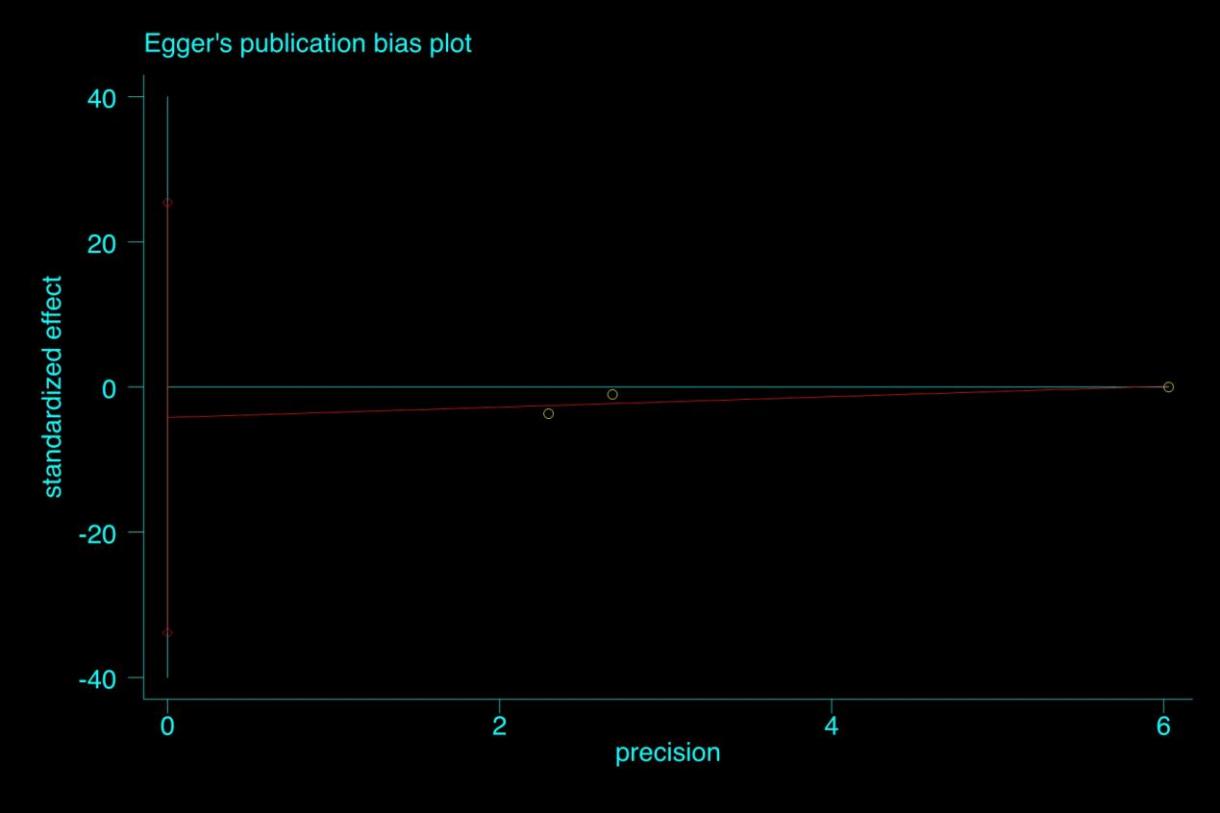


Egger’s publication bias plot of differences in creatinine levels between COVID-19 patients with and without pulmonary air leak (p=0.323, number of events: 3).


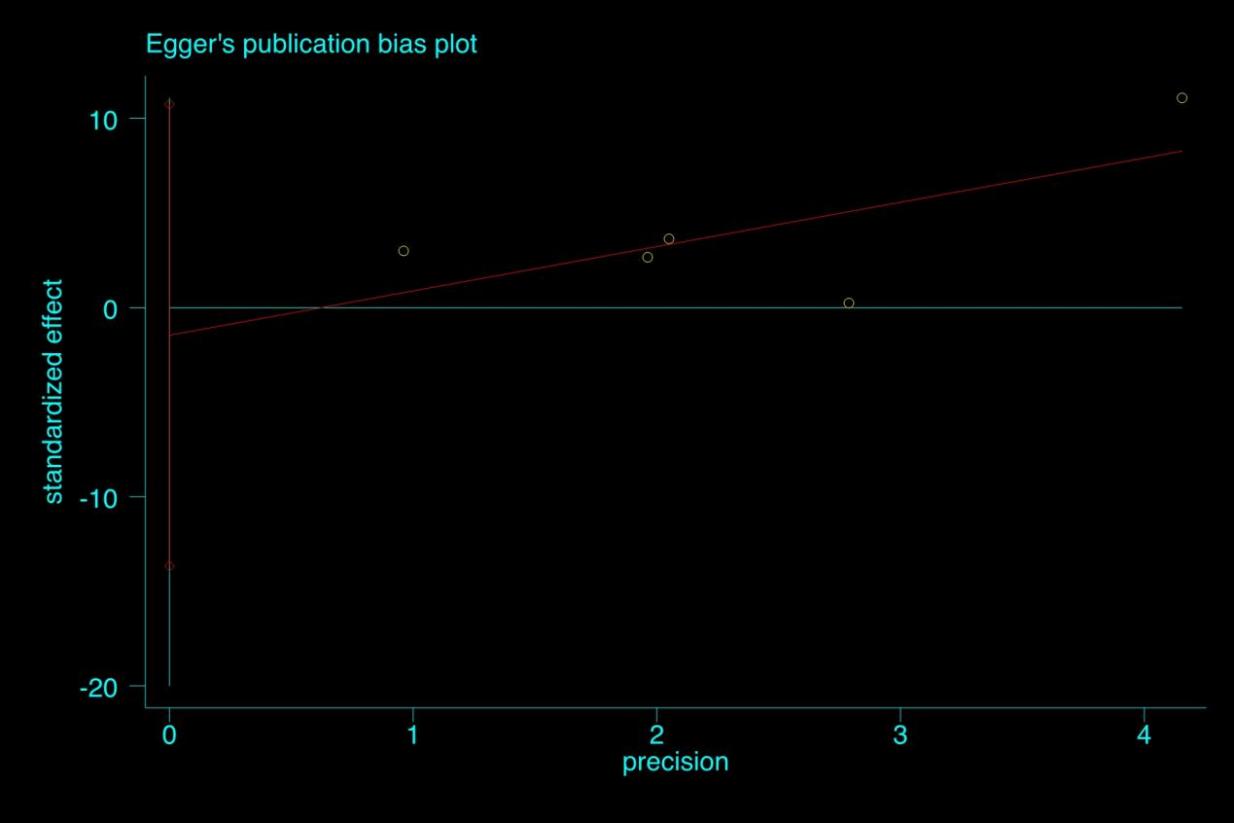


Egger’s publication bias plot of differences in mechanical ventilation between COVID-19 patients with and without pulmonary air leak (p=0.728, number of events: 5).


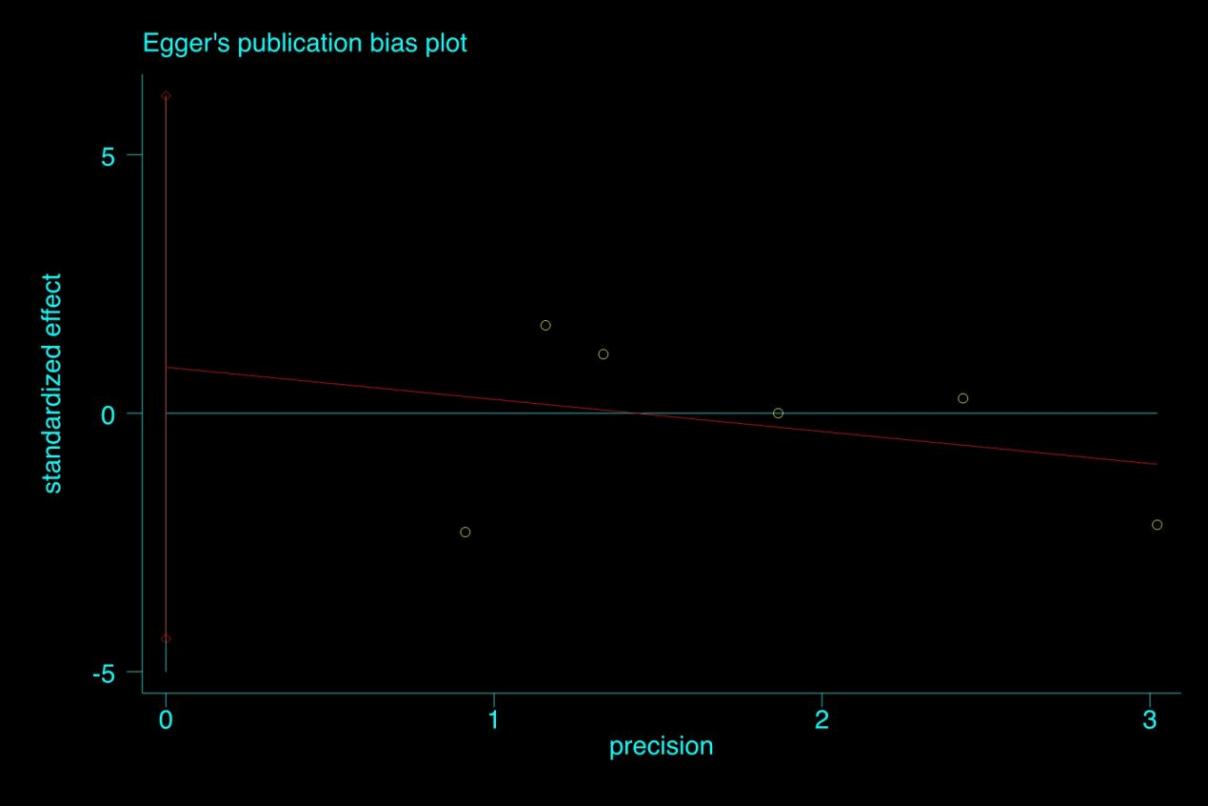


Egger’s publication bias plot of differences in invasive mechanical ventilation between COVID-19 patients with and without pulmonary air leak (p=0.663, number of events: 6).


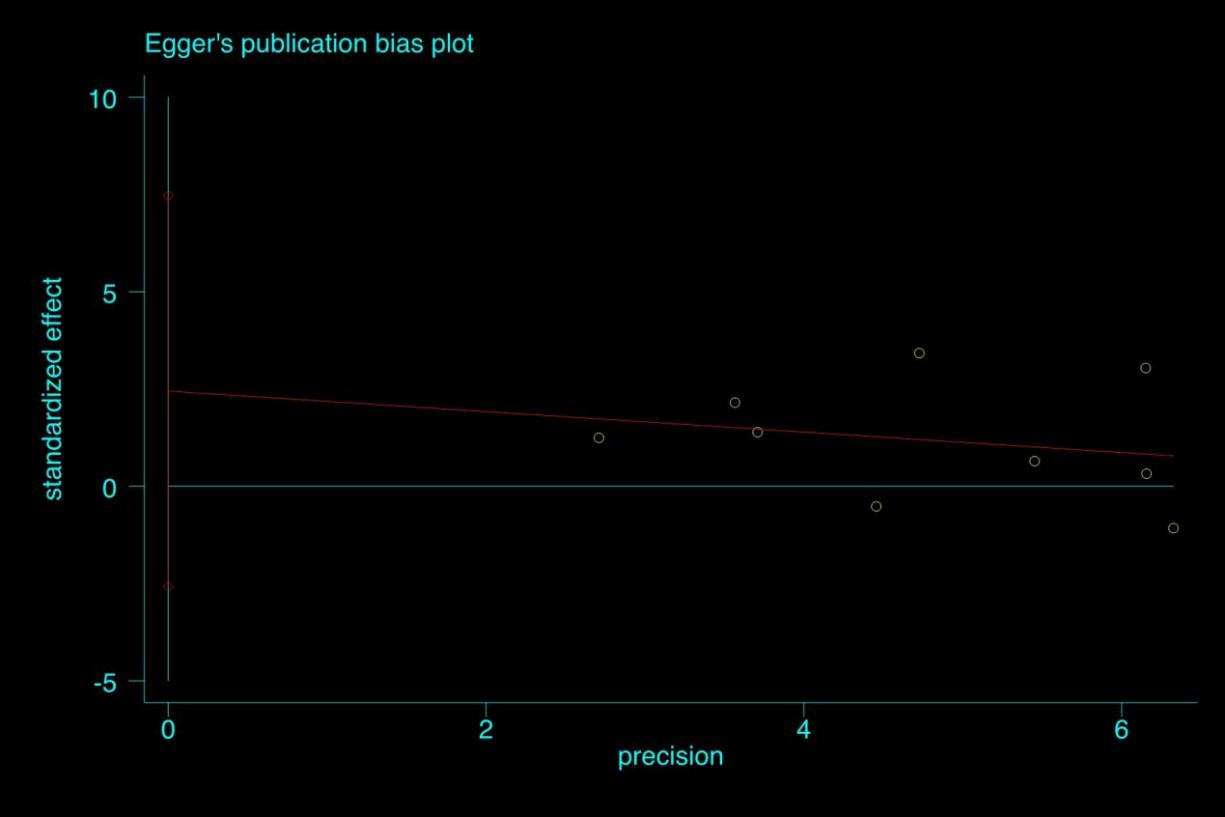


Egger’s publication bias plot of differences in positive end-expiratory pressure between COVID-19 patients with and without pulmonary air leak (p=0.288, number of events: 9).


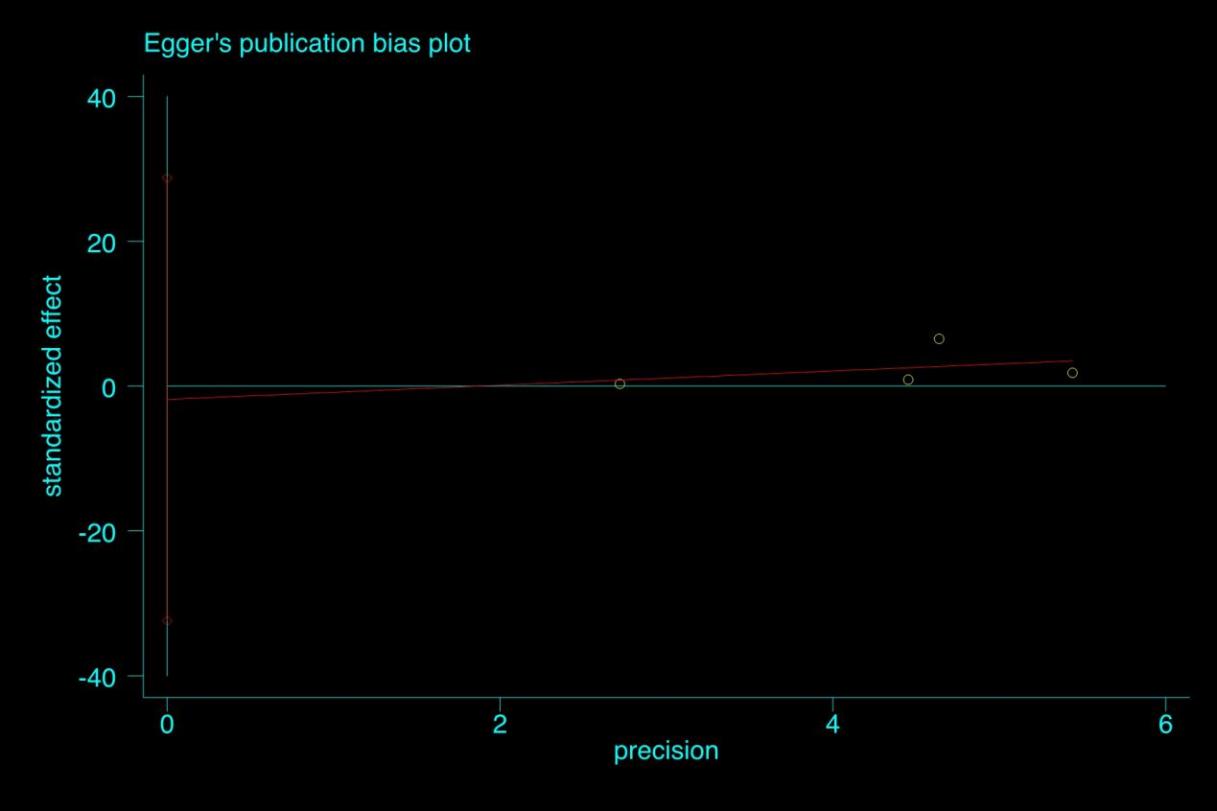


Egger’s publication bias plot of differences in peak inspiratory pressure between

COVID-19 patients with and without pulmonary air leak (p=0.821, number of events: 4).


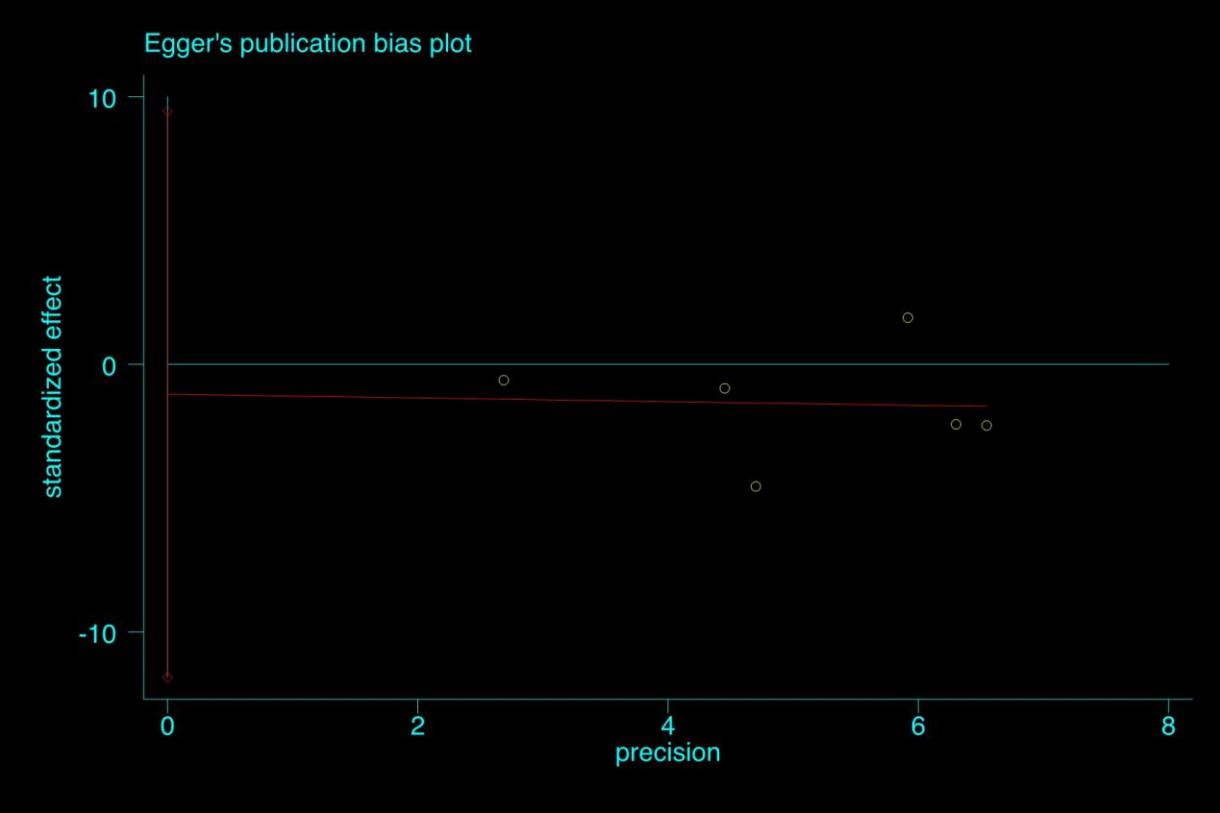


Egger’s publication bias plot of differences in PaO_2_/FiO_2_ ratio between COVID-19 patients with and without pulmonary air leak (p=0.784, number of events: 6).


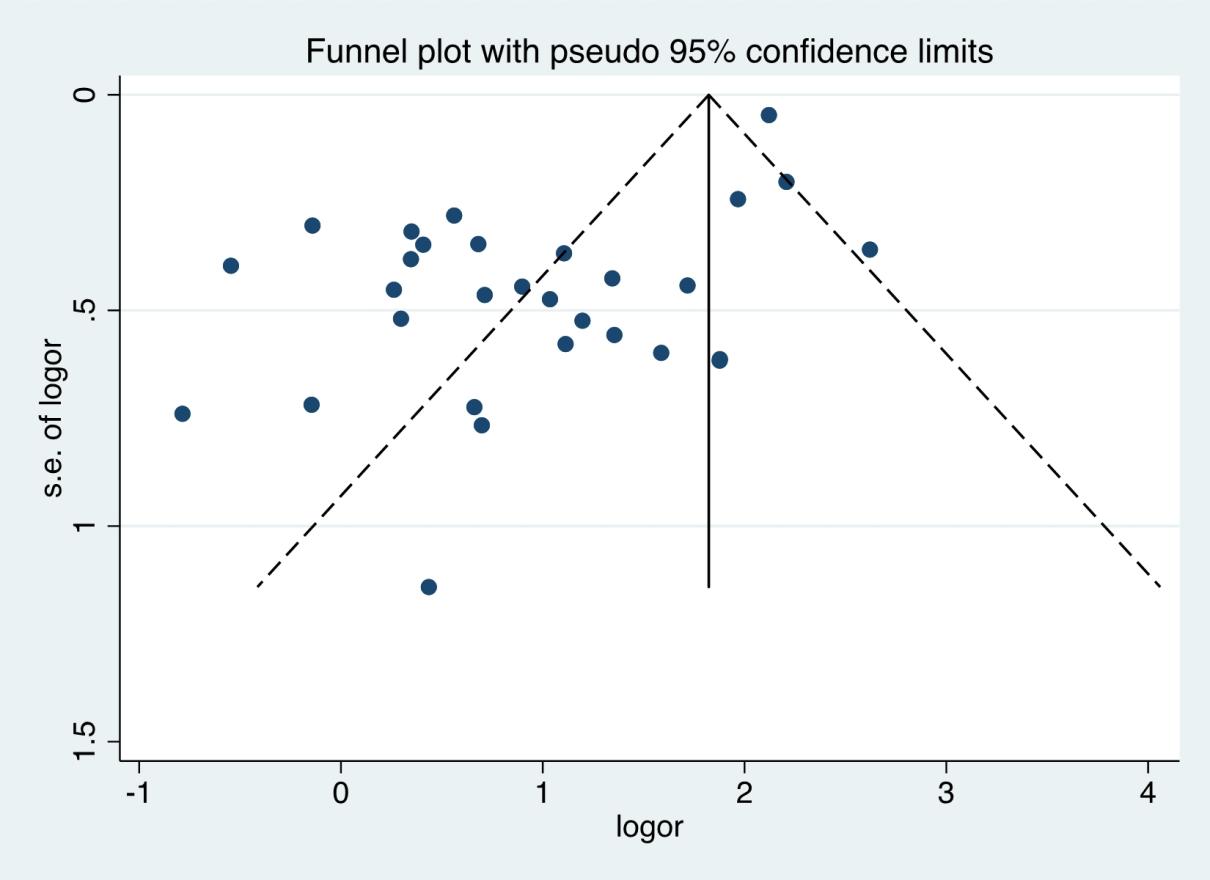


Funnel plot of differences in the mortality rate between COVID-19 patients with and without pulmonary air leak (number of events: 30).


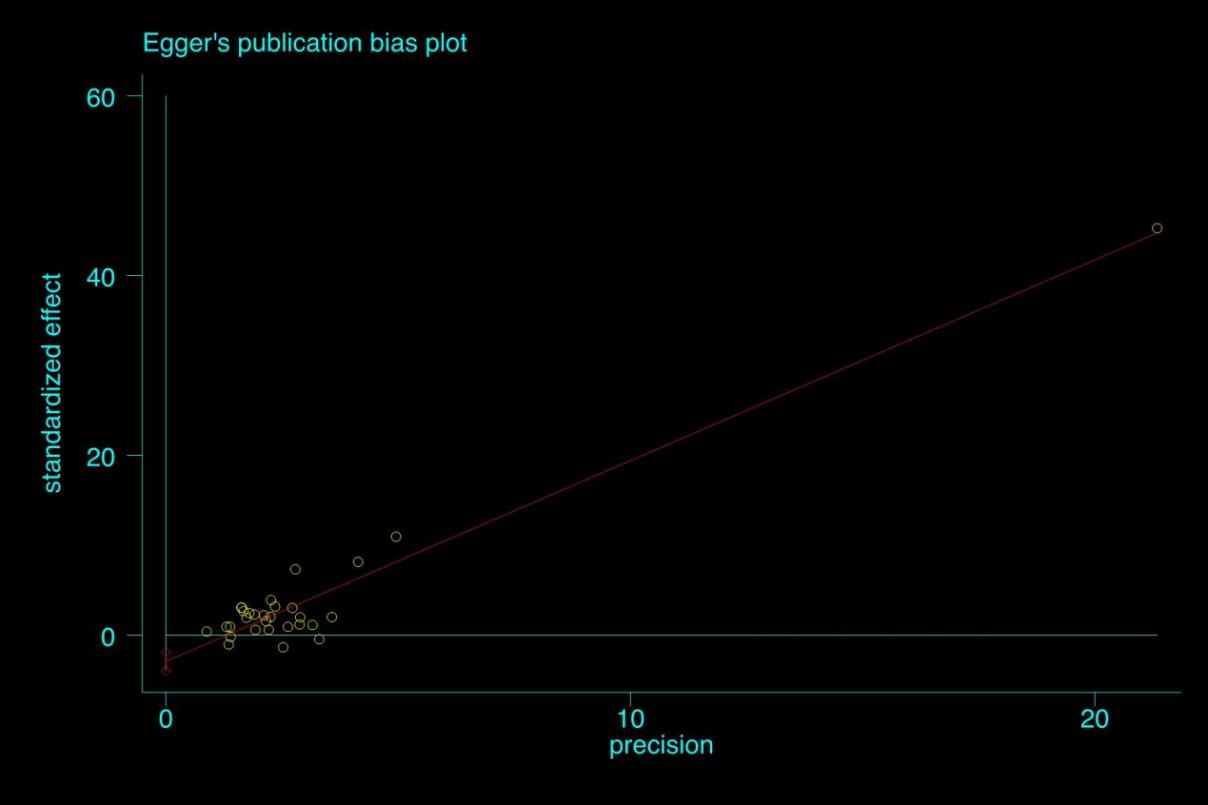


Egger’s publication bias plot of differences in the mortality rate between COVID-19 patients with and without pulmonary air leak (p<0.001, number of events: 30).


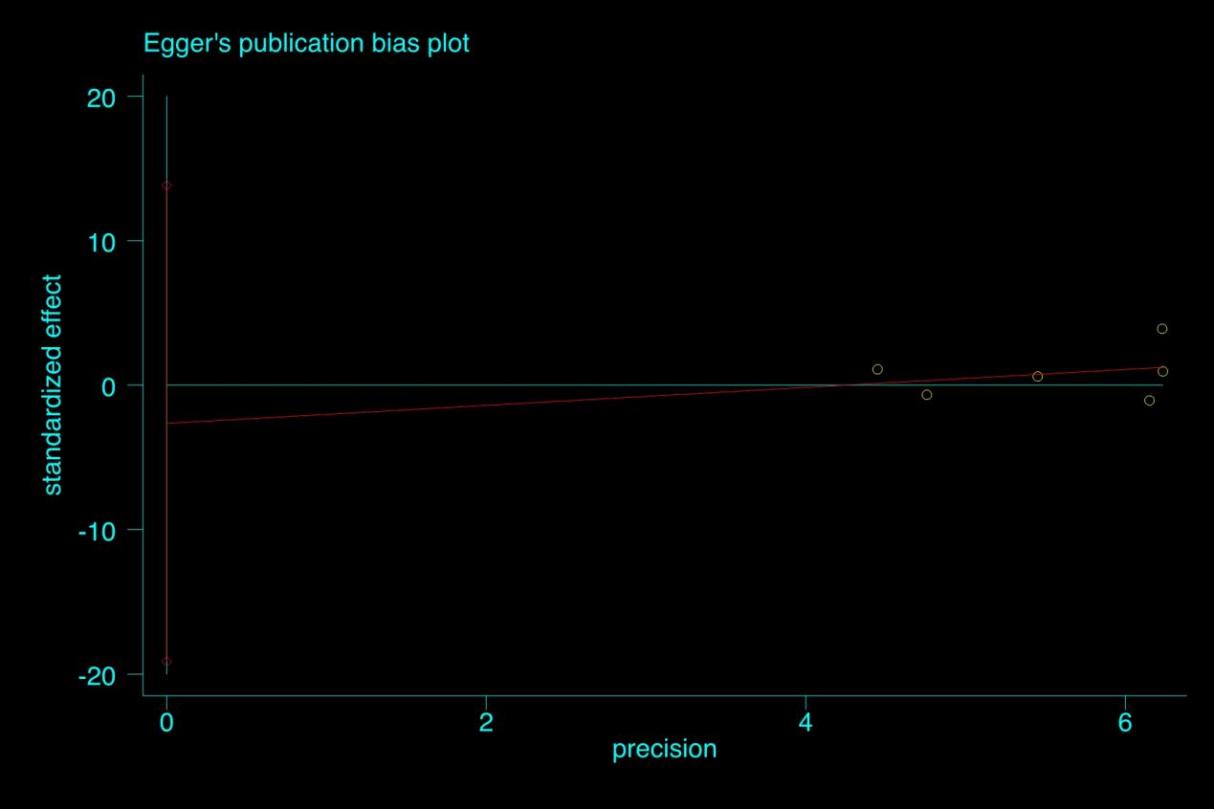


Egger’s publication bias plot of differences in tidal volume between COVID-19 patients with and without pulmonary air leak (p=0.679, number of events: 6).


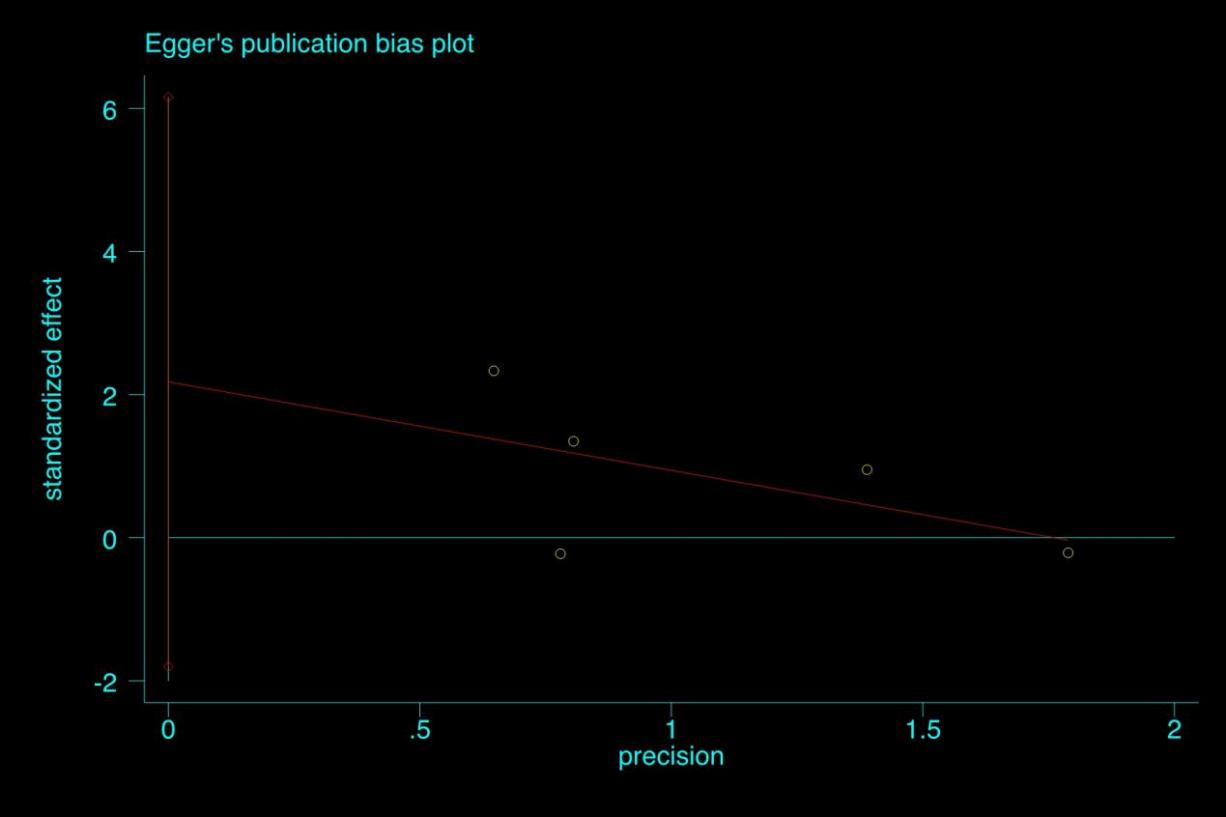


Egger’s publication bias plot of differences in the mortality rate between COVID-19 patients with multiple types of pulmonary air leak at the same time and those with only one type (p=0.180, number of events: 5).
