# Supplementary material for: Effects of pulmonary air leak on patients with coronavirus disease 2019 (COVID-19): a systematic review and meta-analysis
Source: BMC Pulm Med. 2023 Oct 19;23:398. doi: 10.1186/s12890-023-02710-2 (PMC10588255; doi:10.1186/s12890-023-02710-2)
Supplement: Supplementary file 3 — Supplementary Material 3 [file 12890_2023_2710_MOESM3_ESM.docx]

**Supplementary tables and figures.
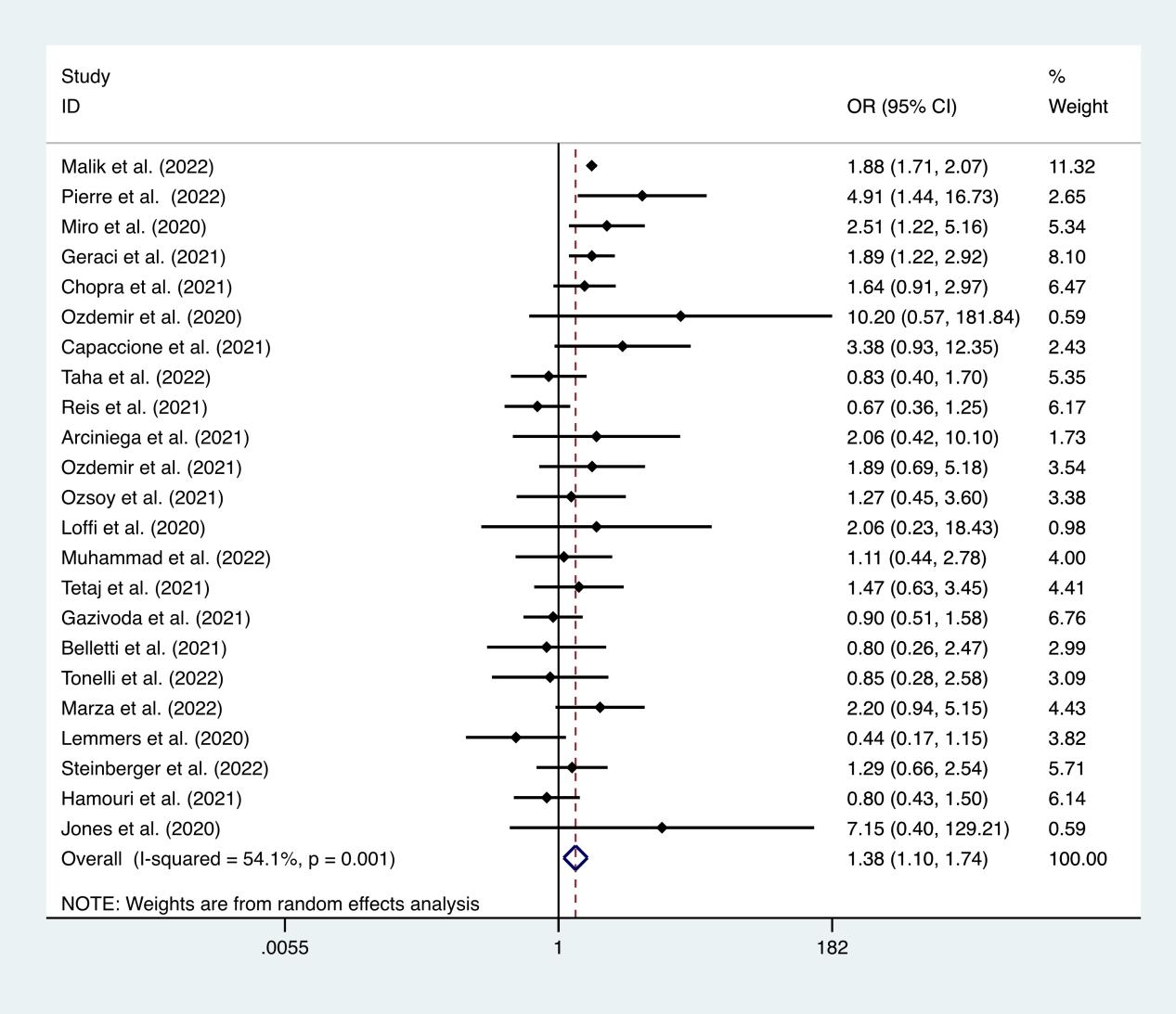
**

**Figure S1**: Forest plot of differences in sex between COVID-19 patients with and without pulmonary air leak (number of events: 23).


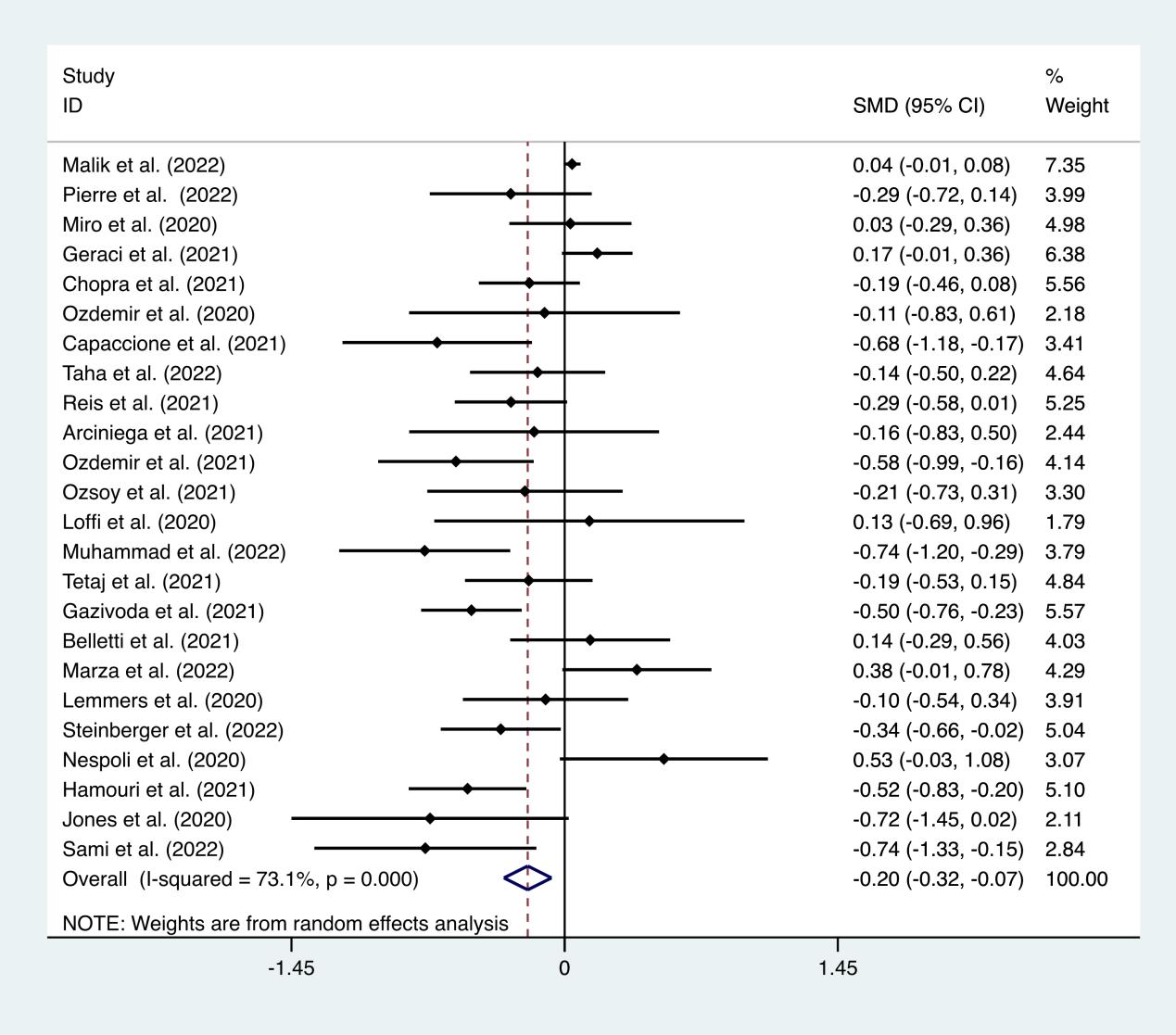


**Figure S2**: Forest plot of differences in age between COVID-19 patients with and without pulmonary air leak (number of events: 24).


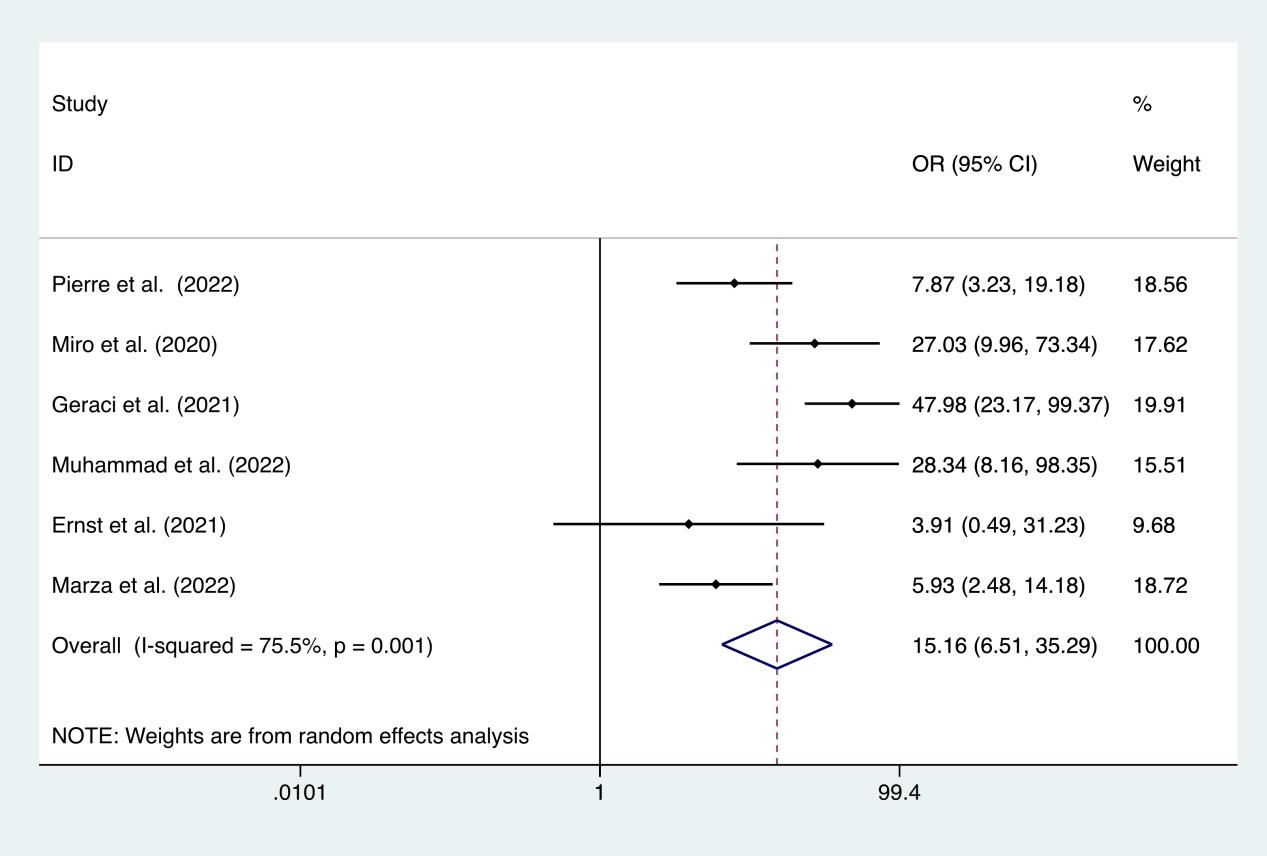


**Figure S3**: Forest plot of differences in [intensive care unit](javascript:;) admission between COVID-19 patients with and without pulmonary air leak (number of events: 6).


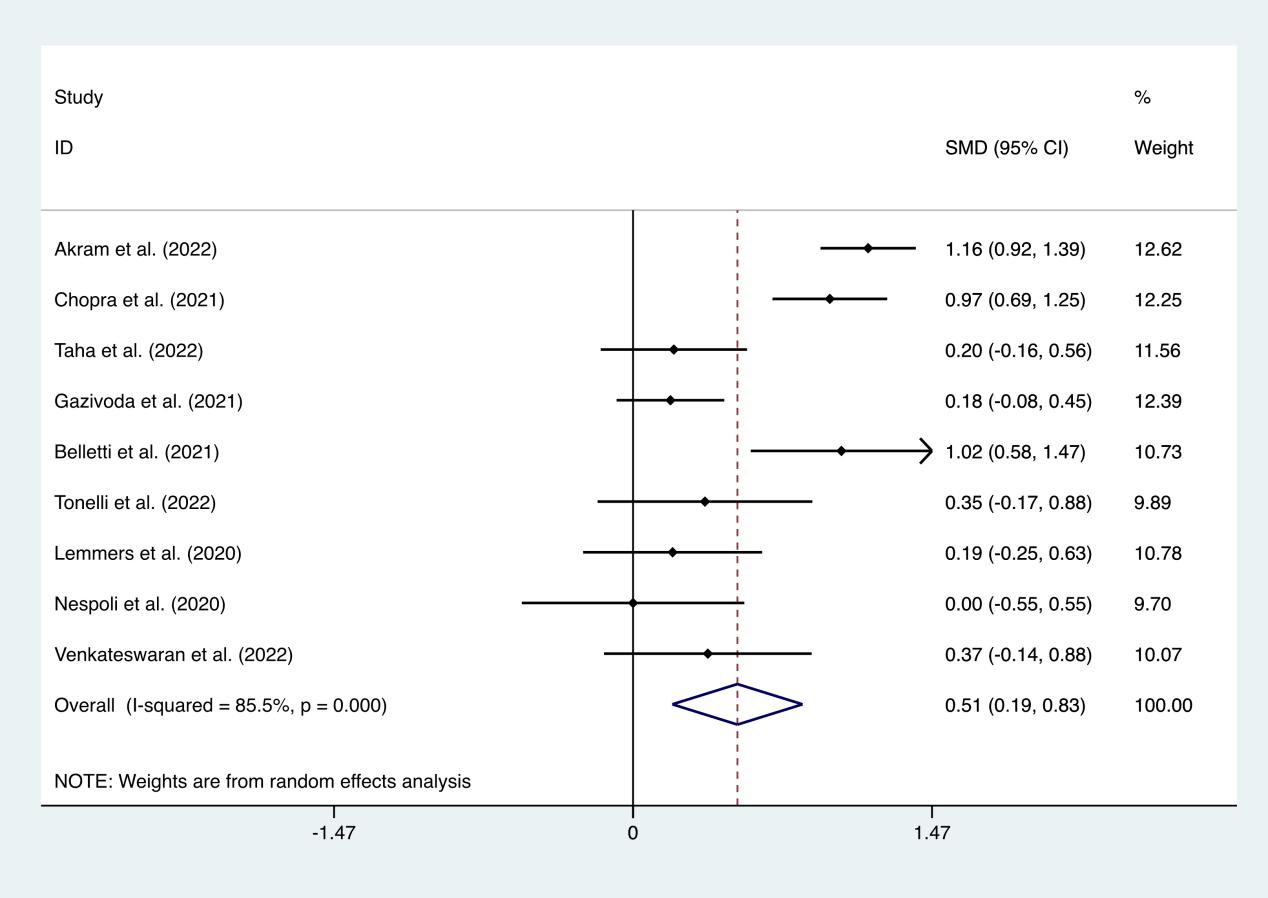


**Figure S4**: Forest plot of differences in [intensive care unit](javascript:;) stay between COVID-19

patients with and without pulmonary air leak (number of events: 10).


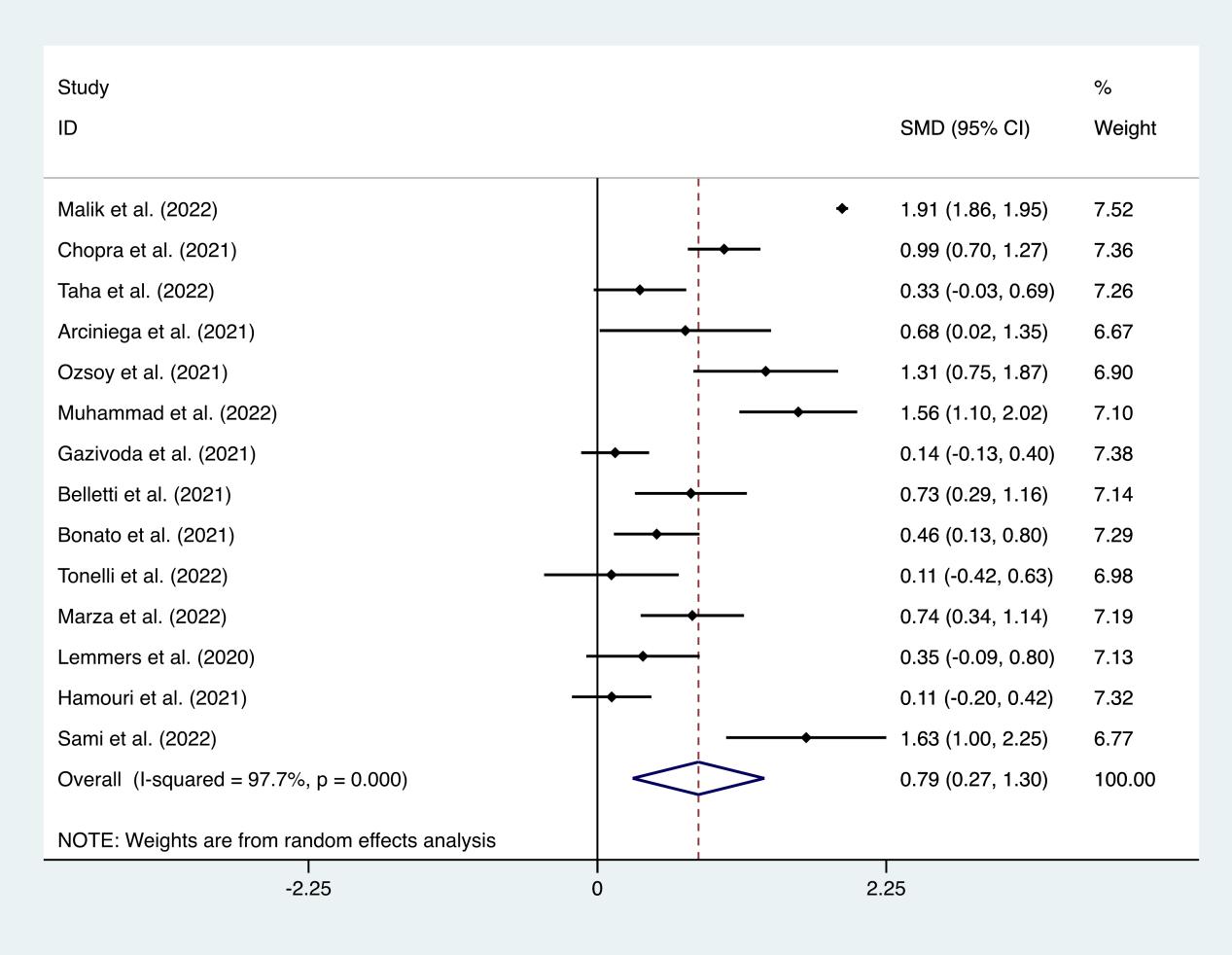


**Figure S5**: Forest plot of differences in hospital stay between COVID-19 patients with

and without pulmonary air leak (number of events: 14).


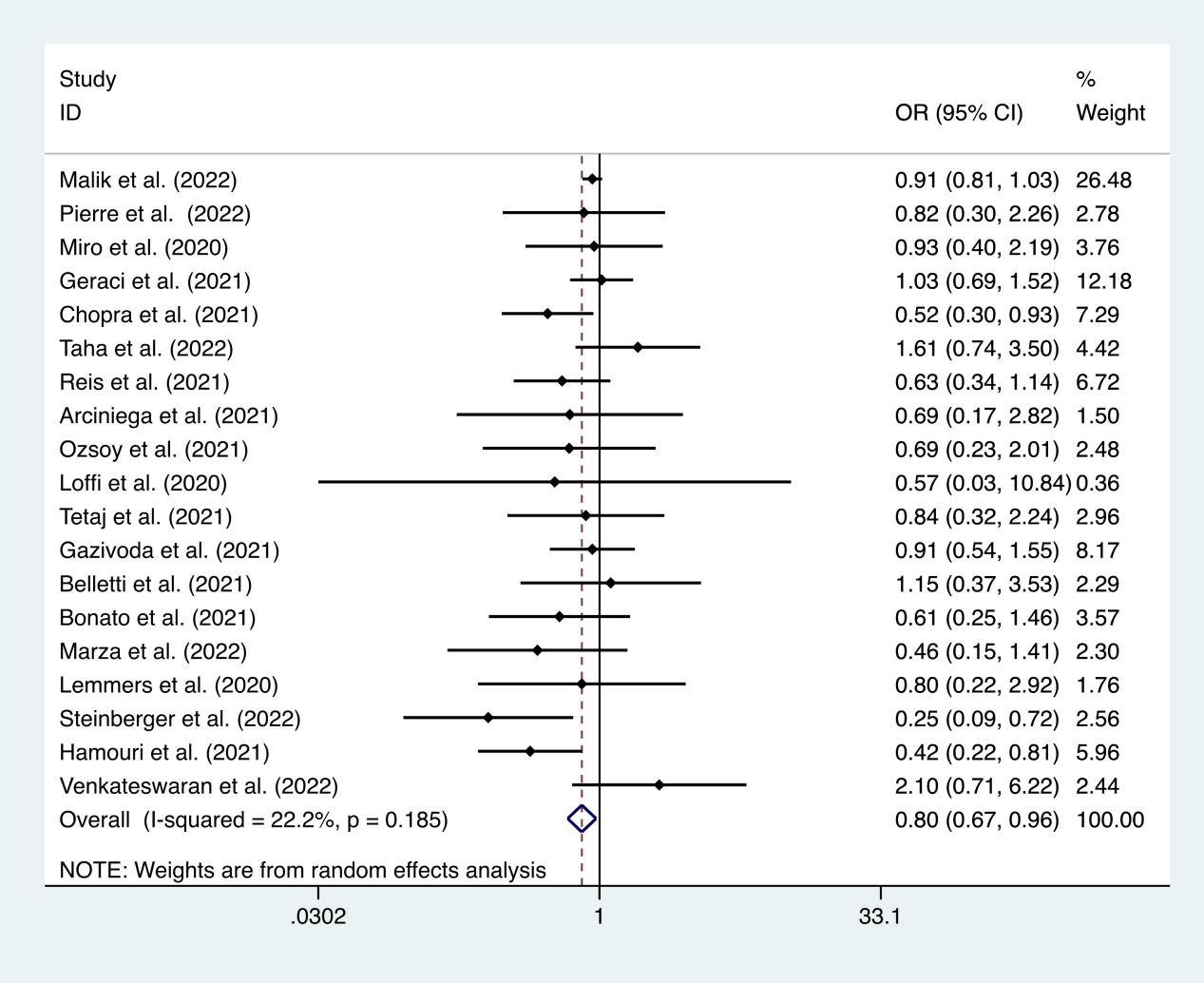


**Figure S6**: Forest plot of differences in diabetes between COVID-19 patients with and

without pulmonary air leak (number of events: 19).


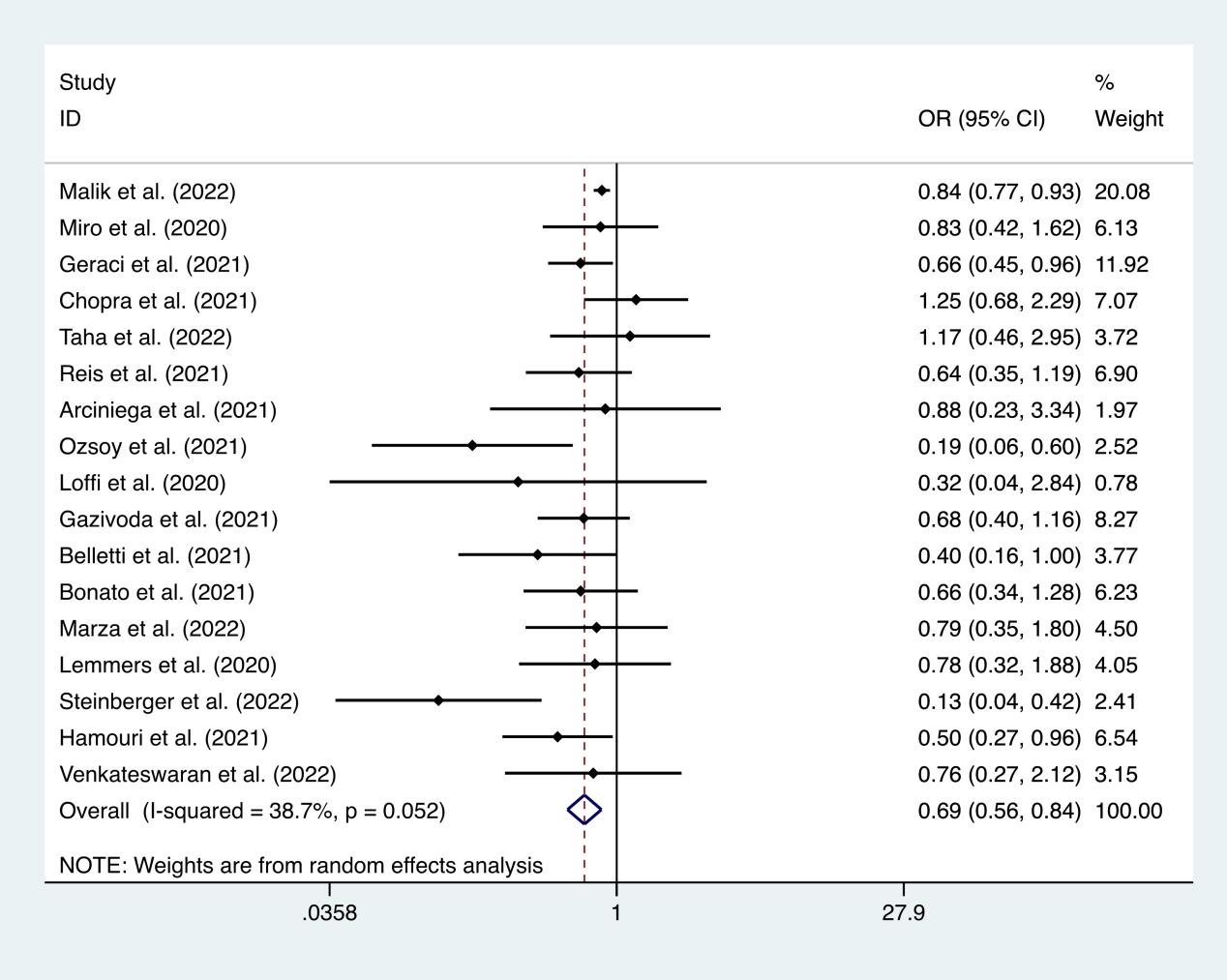


**Figure S7**: Forest plot of differences in hypertension between COVID-19 patients with

and without pulmonary air leak (number of events: 17).


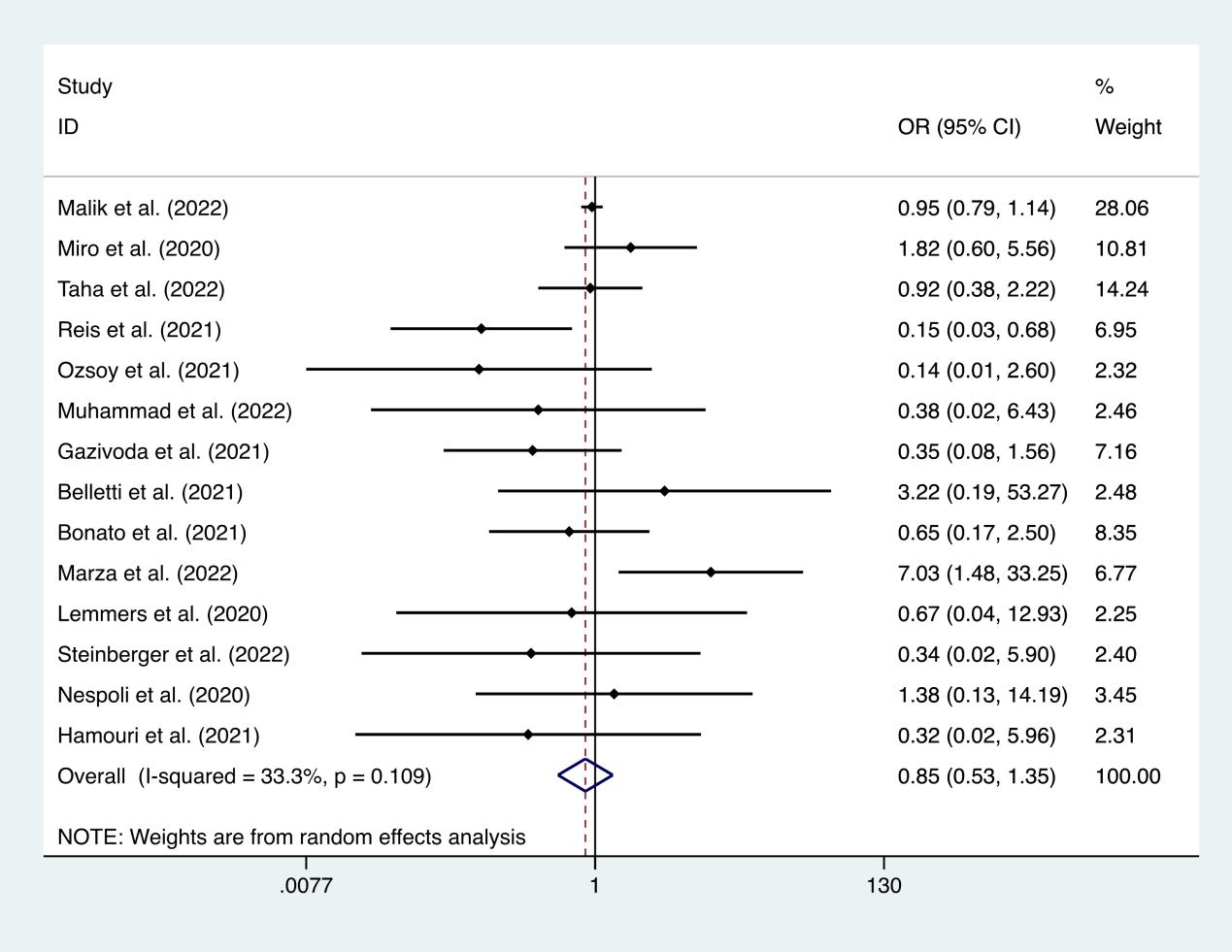


**Figure S8**: Forest plot of differences in chronic obstructive pulmonary disease between COVID-19 patients with and without pulmonary air leak (number of events: 14).


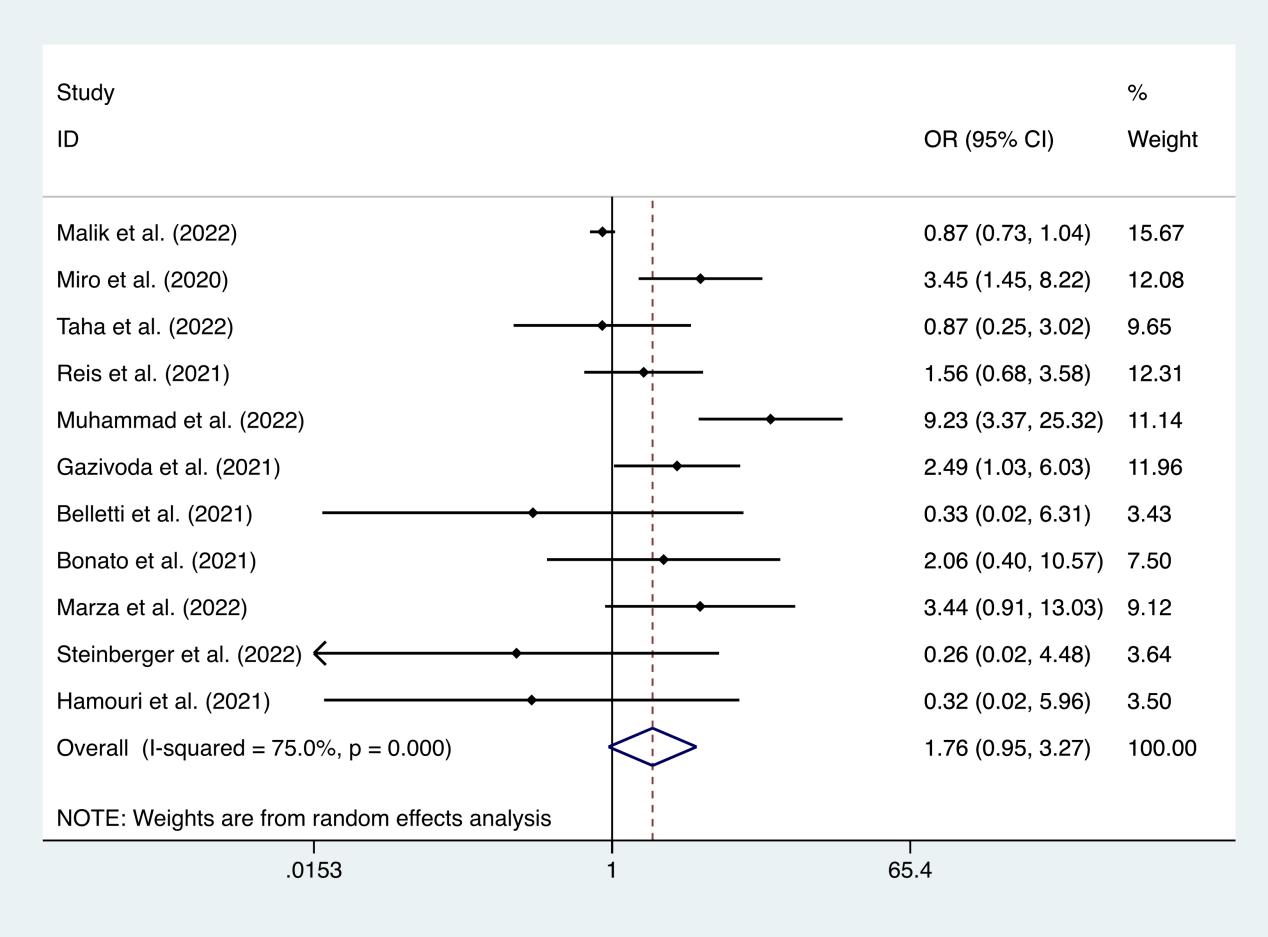


**Figure S9**: Forest plot of differences in asthma between COVID-19 patients with and

without pulmonary air leak (number of events: 11).


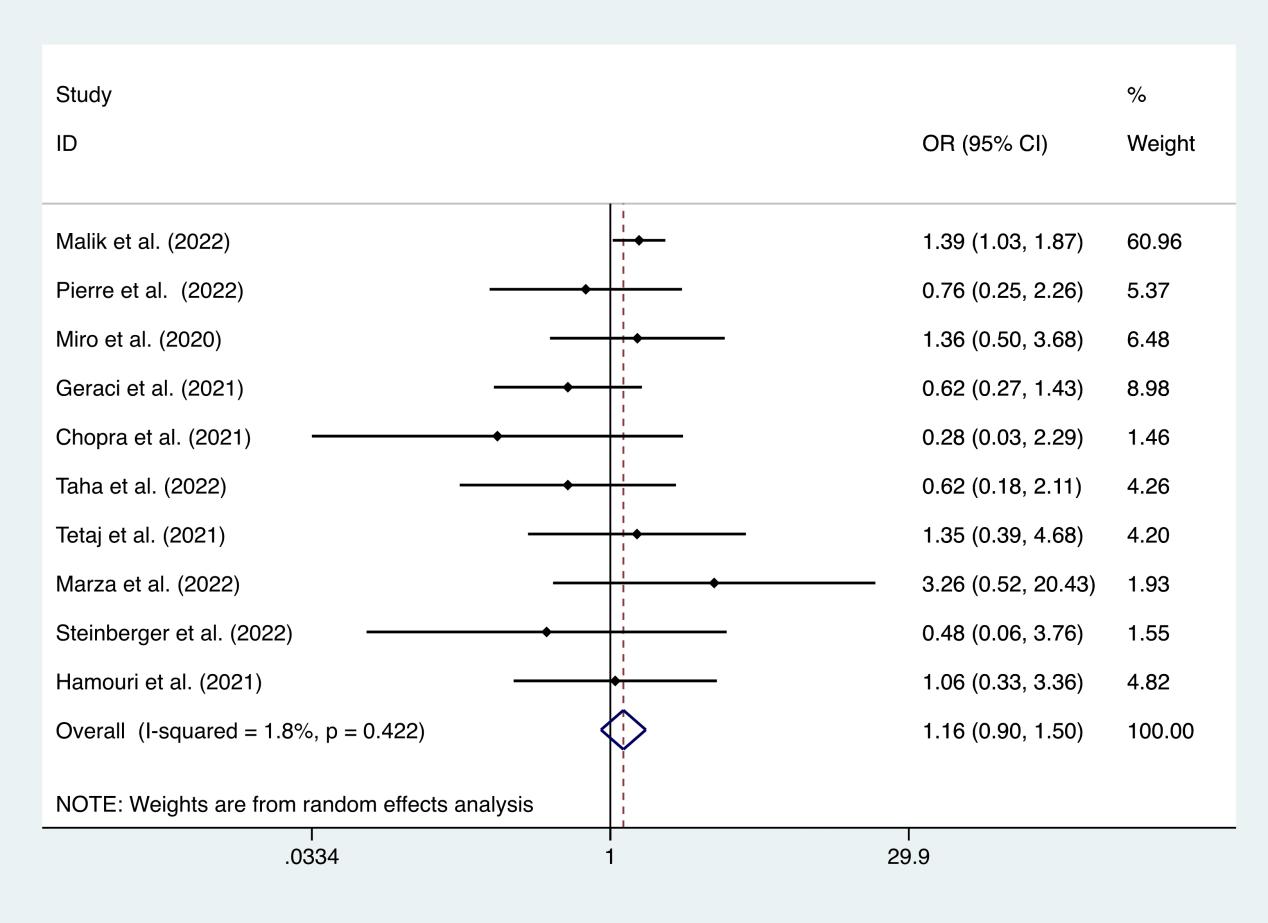


**Figure S10**: Forest plot of differences in cancer between COVID-19 patients with and

without pulmonary air leak (number of events: 10).


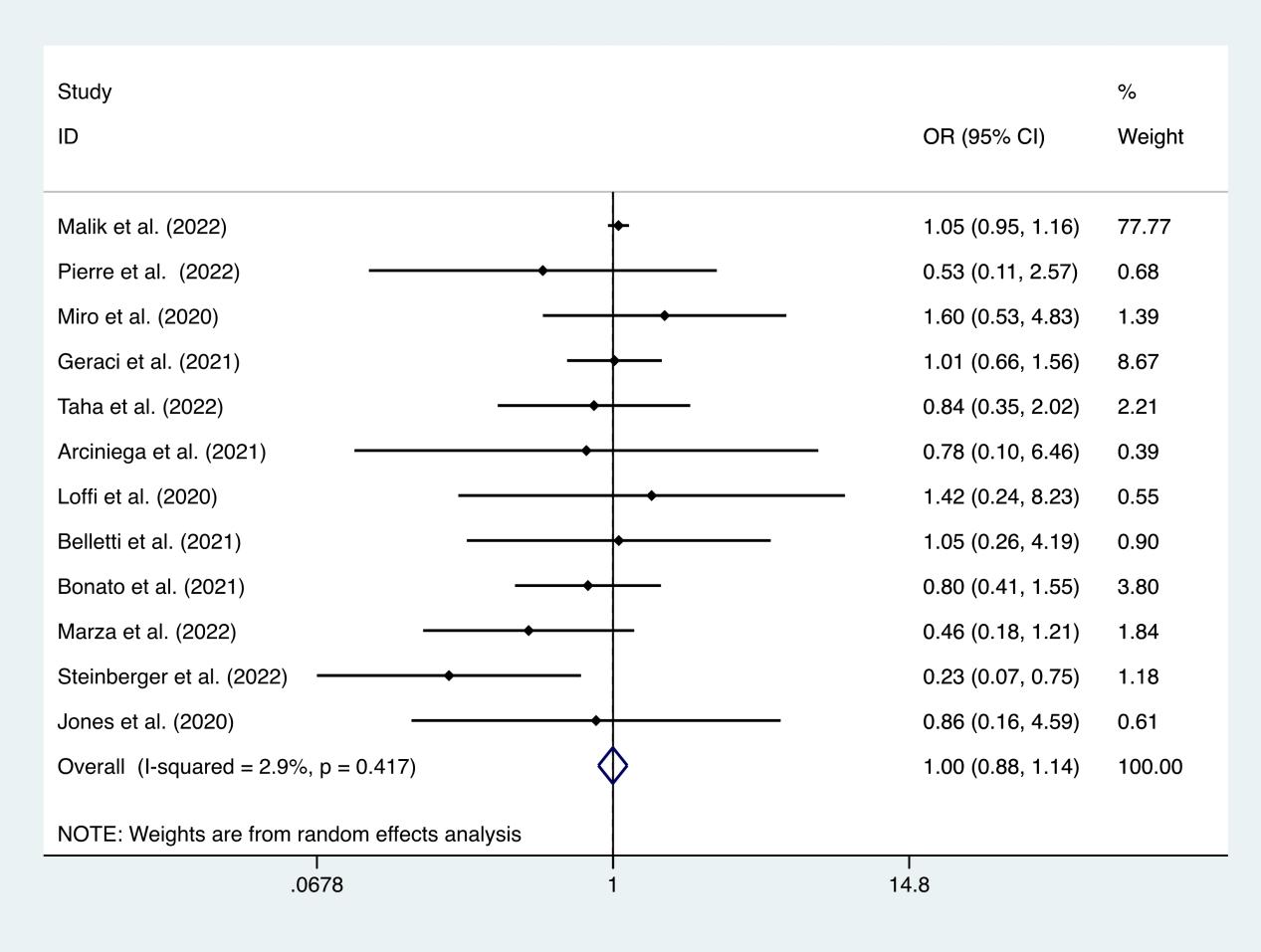


**Figure S11**: Forest plot of differences in smoking between COVID-19 patients with and without pulmonary air leak (number of events: 12).


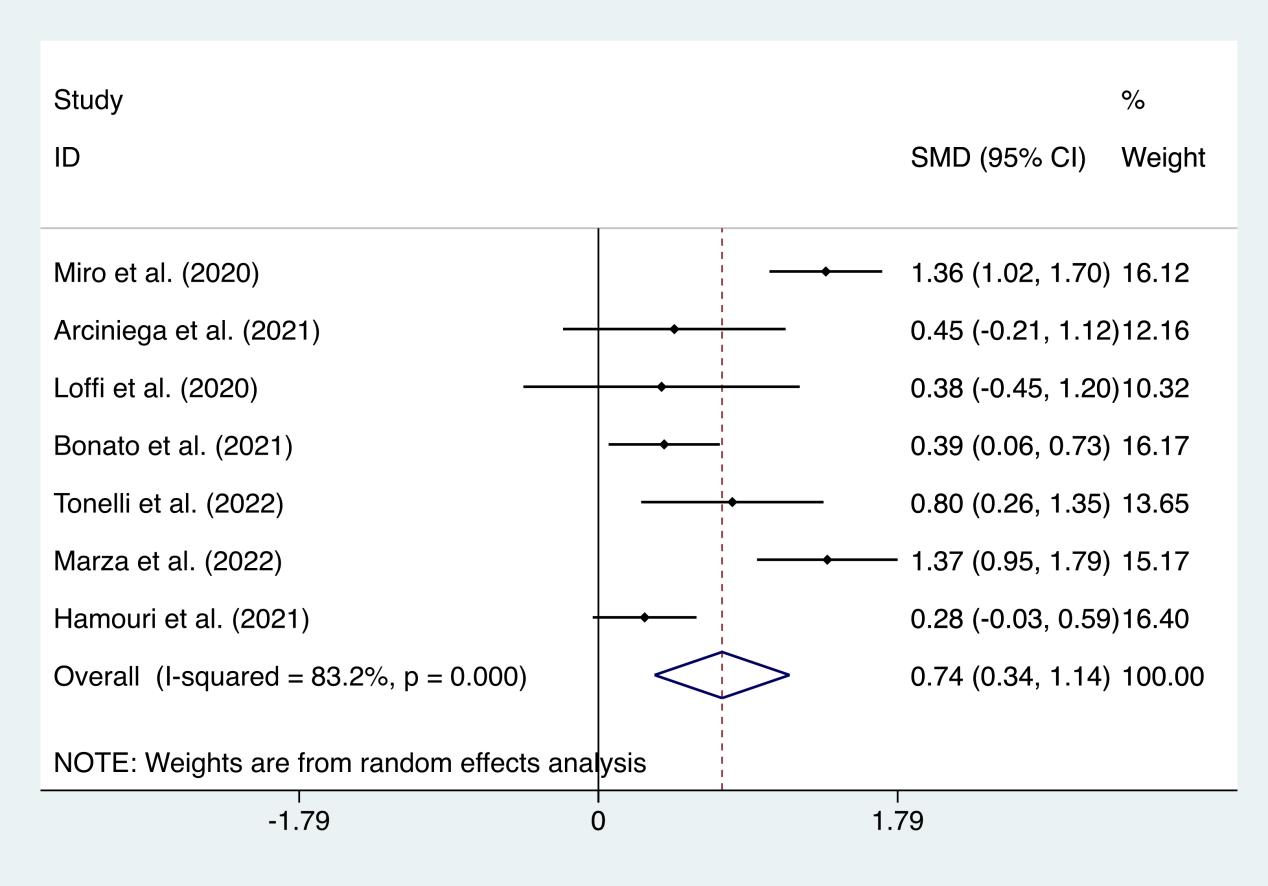


**Figure S12**: Forest plot of differences in D-dimer levels between COVID-19 patients with and without pulmonary air leak (number of events: 7).


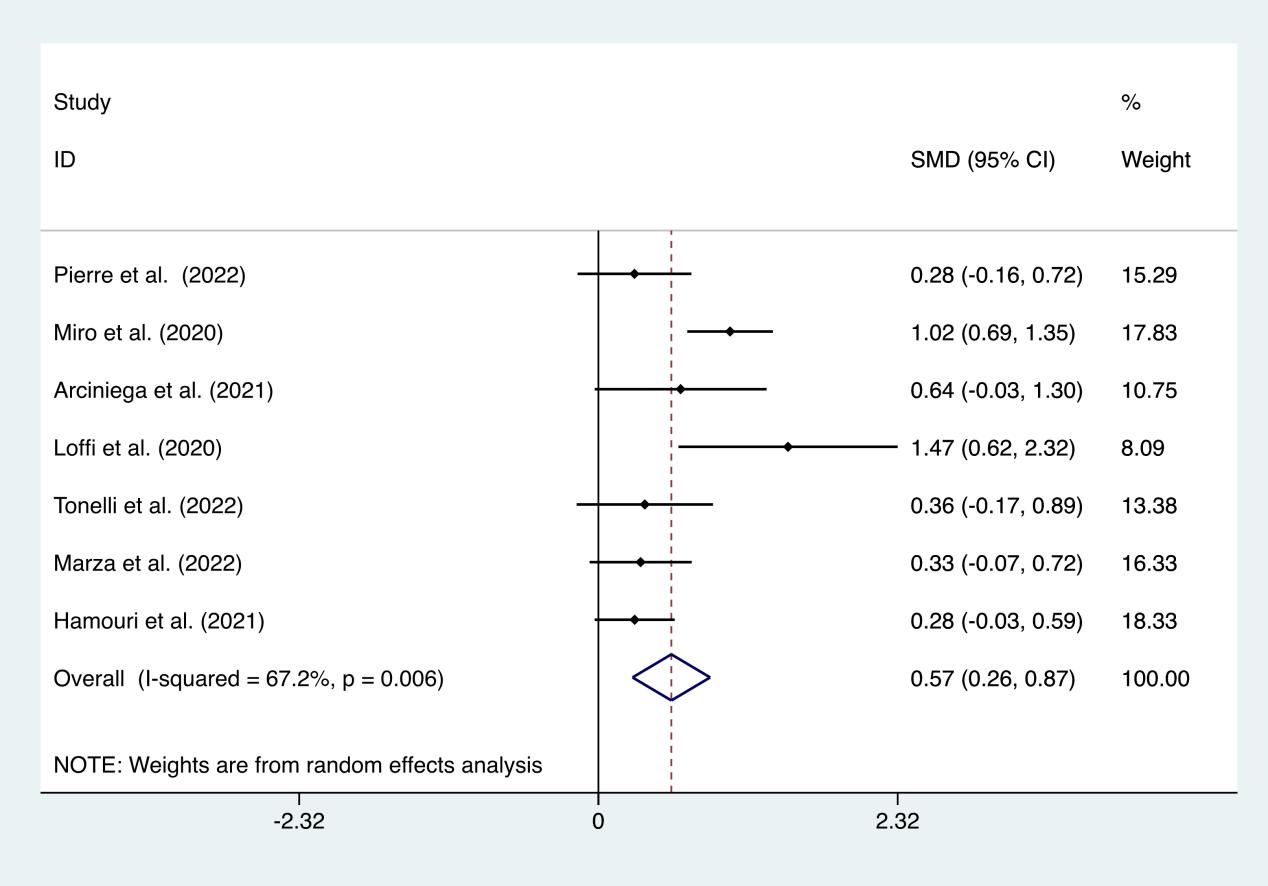


**Figure S13**: Forest plot of differences in leucocyte counts between COVID-19 patients with and without pulmonary air leak (number of events: 7).


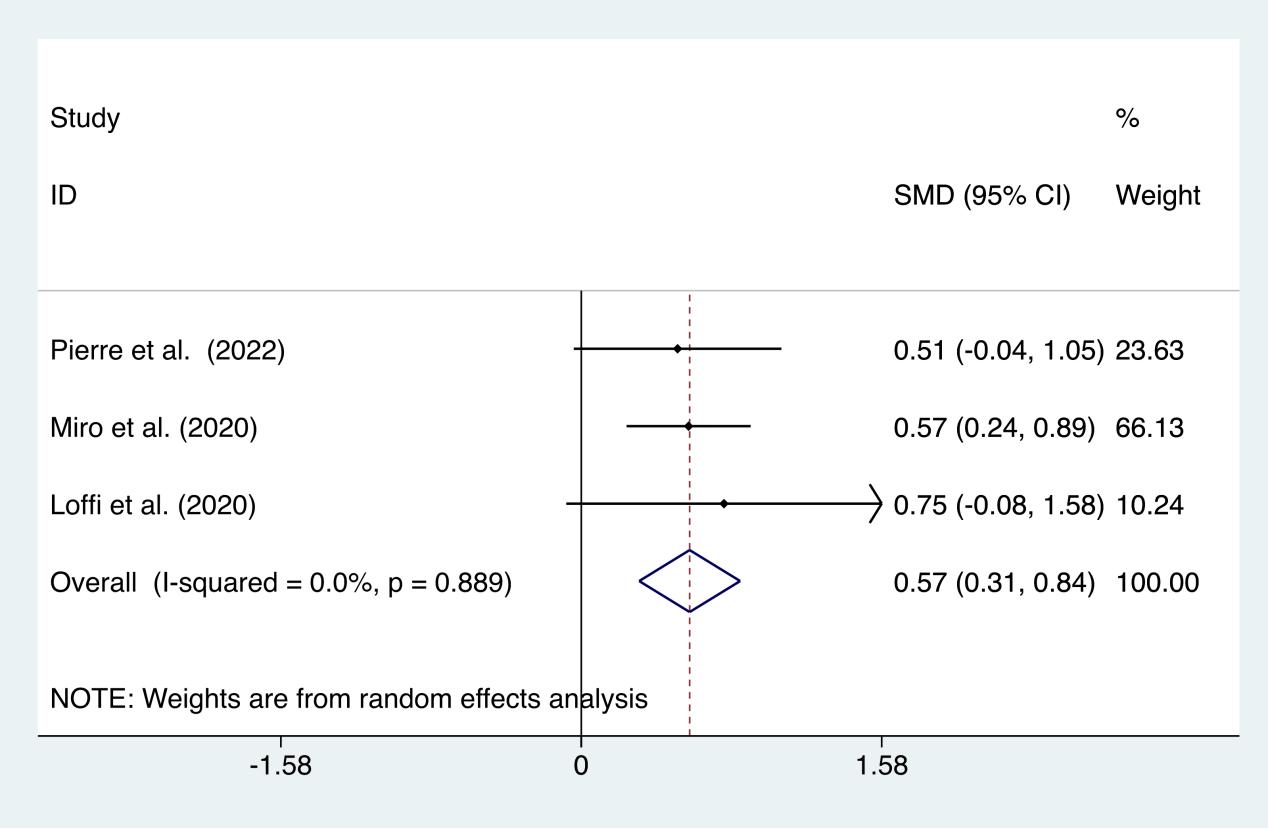


**Figure S14**: Forest plot of differences in aspartate aminotransferase levels between COVID-19 patients with and without pulmonary air leak (number of events: 3).


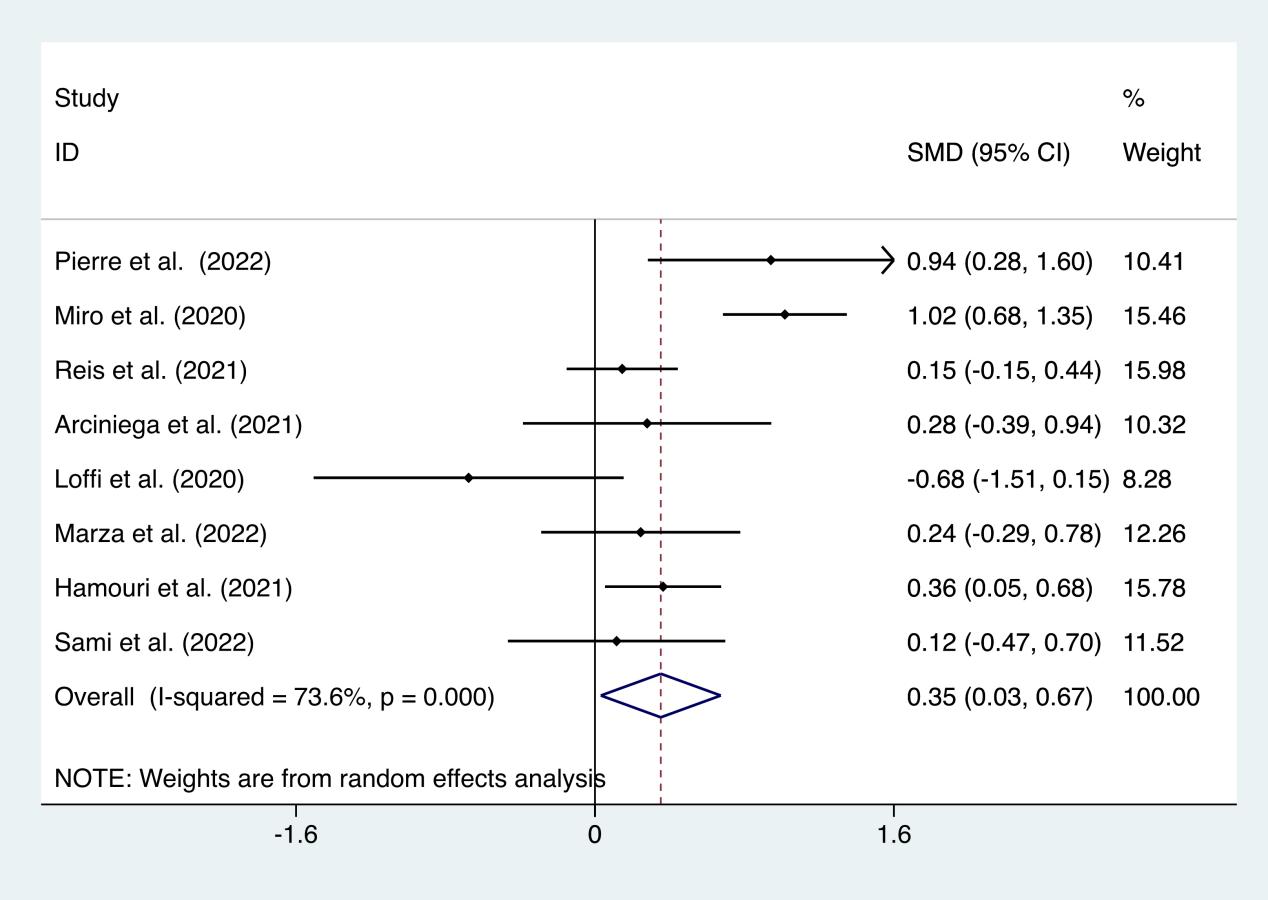


**Figure S15**: Forest plot of differences in lactate dehydrogenase levels between COVID-19

patients with and without pulmonary air leak (number of events: 8).


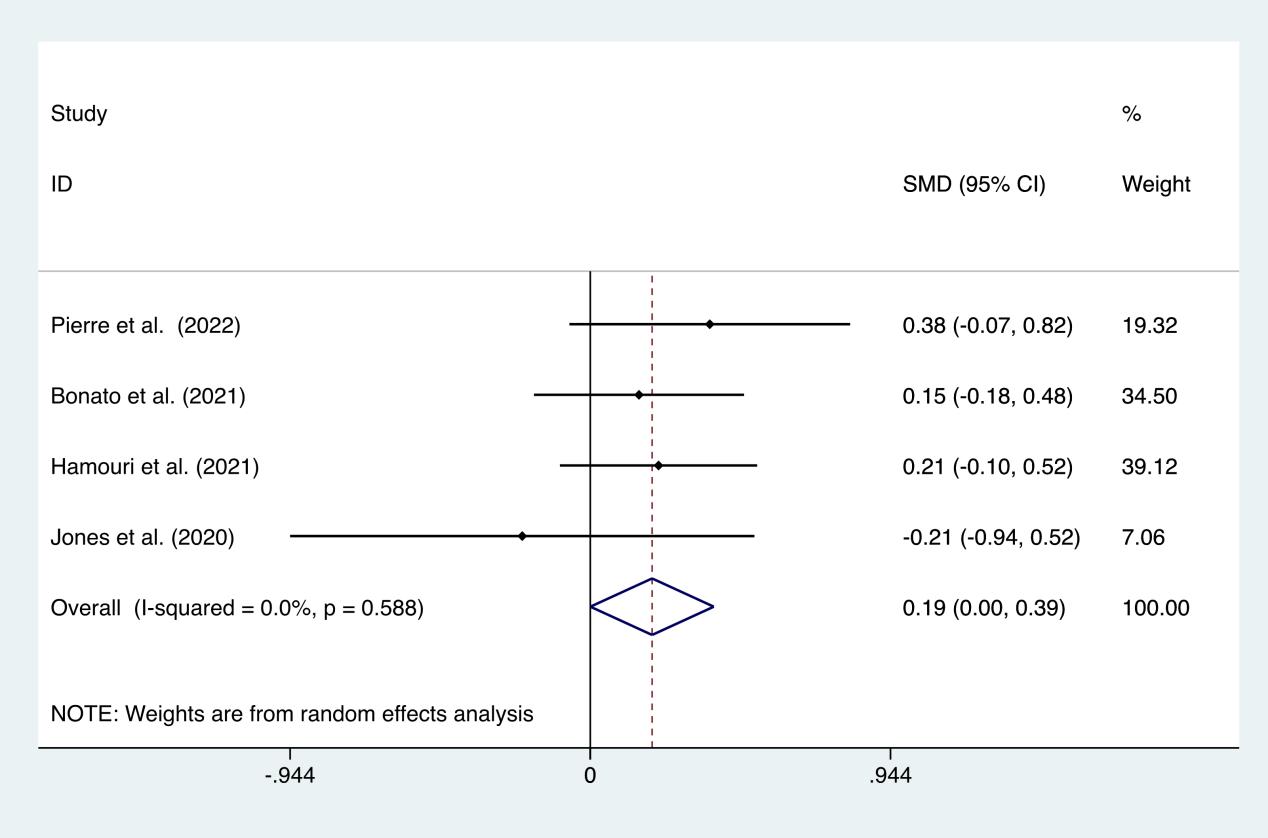


**Figure S16**: Forest plot of differences in neutrophil counts between COVID-19 patients with and without pulmonary air leak (number of events: 4).


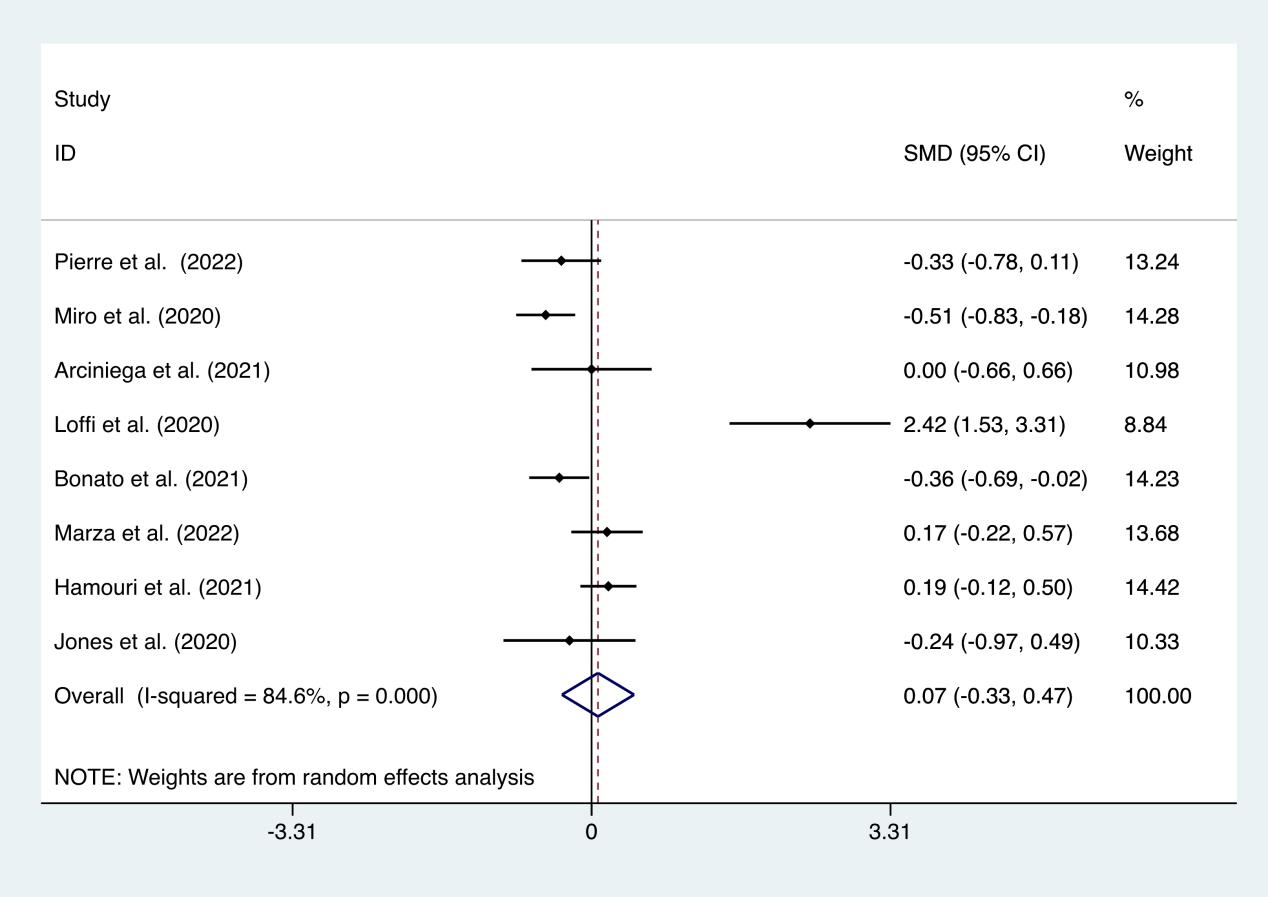


**Figure S17**: Forest plot of differences in lymphocyte counts between COVID-19 patients with and without pulmonary air leak (number of events: 8).


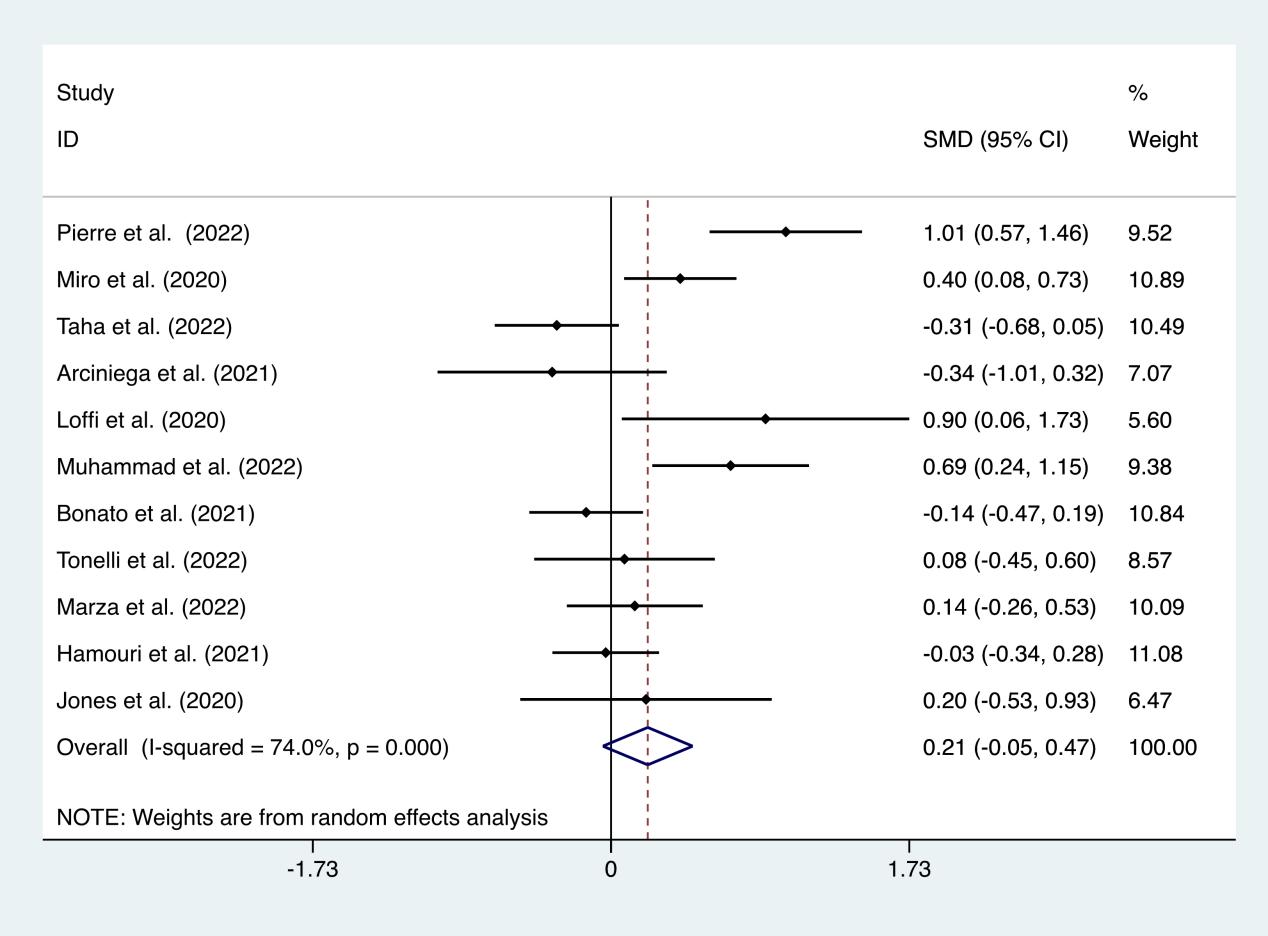


**Figure S18**: Forest plot of differences in C-reactive protein levels between COVID-19 patients with and without pulmonary air leak (number of events: 11).


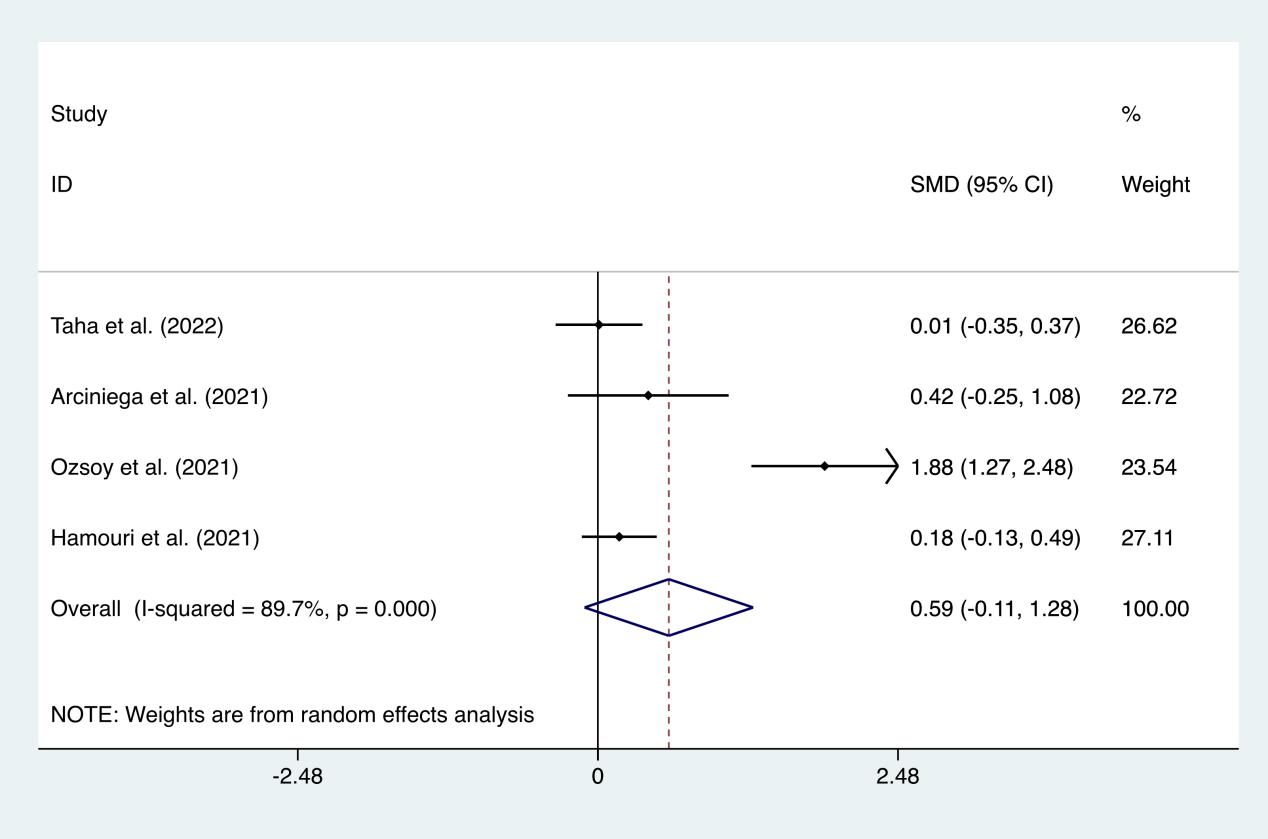


**Figure S19**: Forest plot of differences in ferritin levels between COVID-19 patients with and

without pulmonary air leak (number of events: 4).


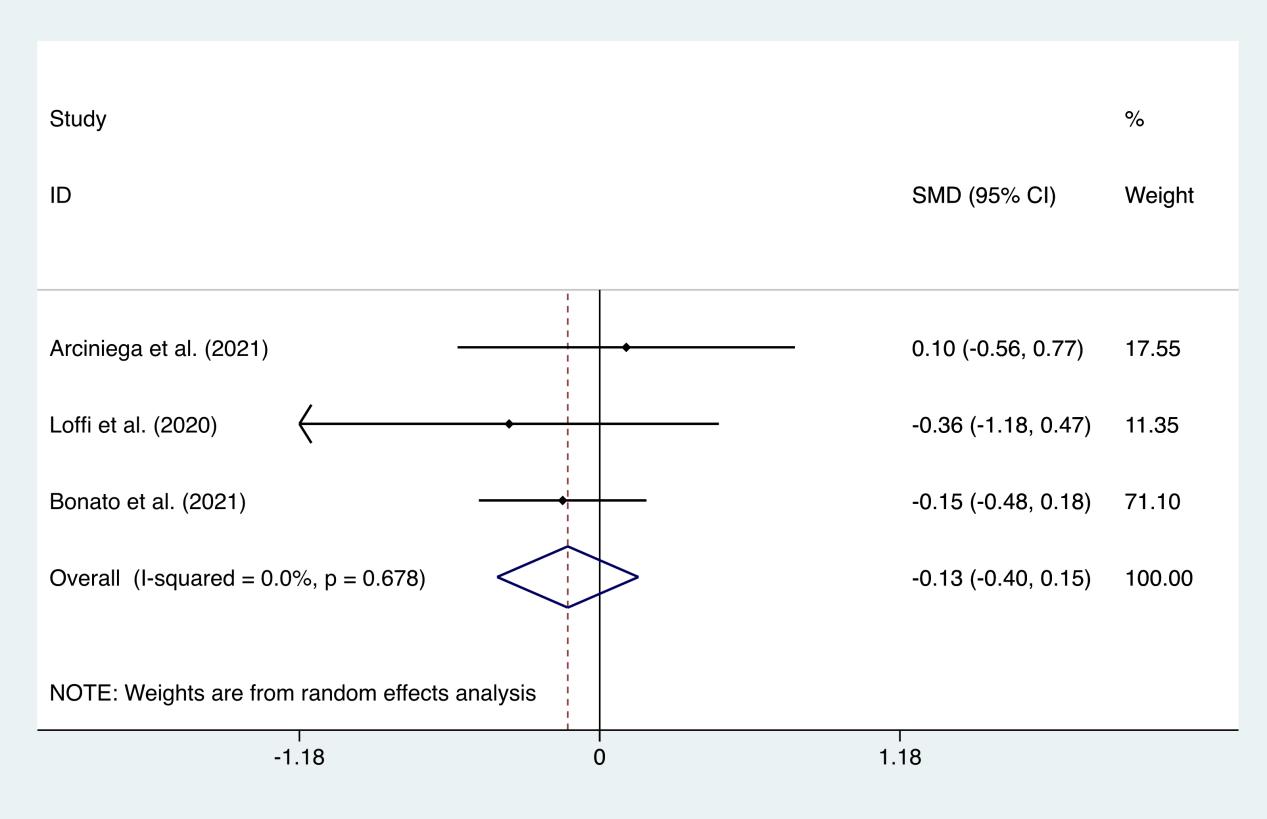


**Figure S20**: Forest plot of differences in platelet counts between COVID-19 patients with and without pulmonary air leak (number of events: 3).


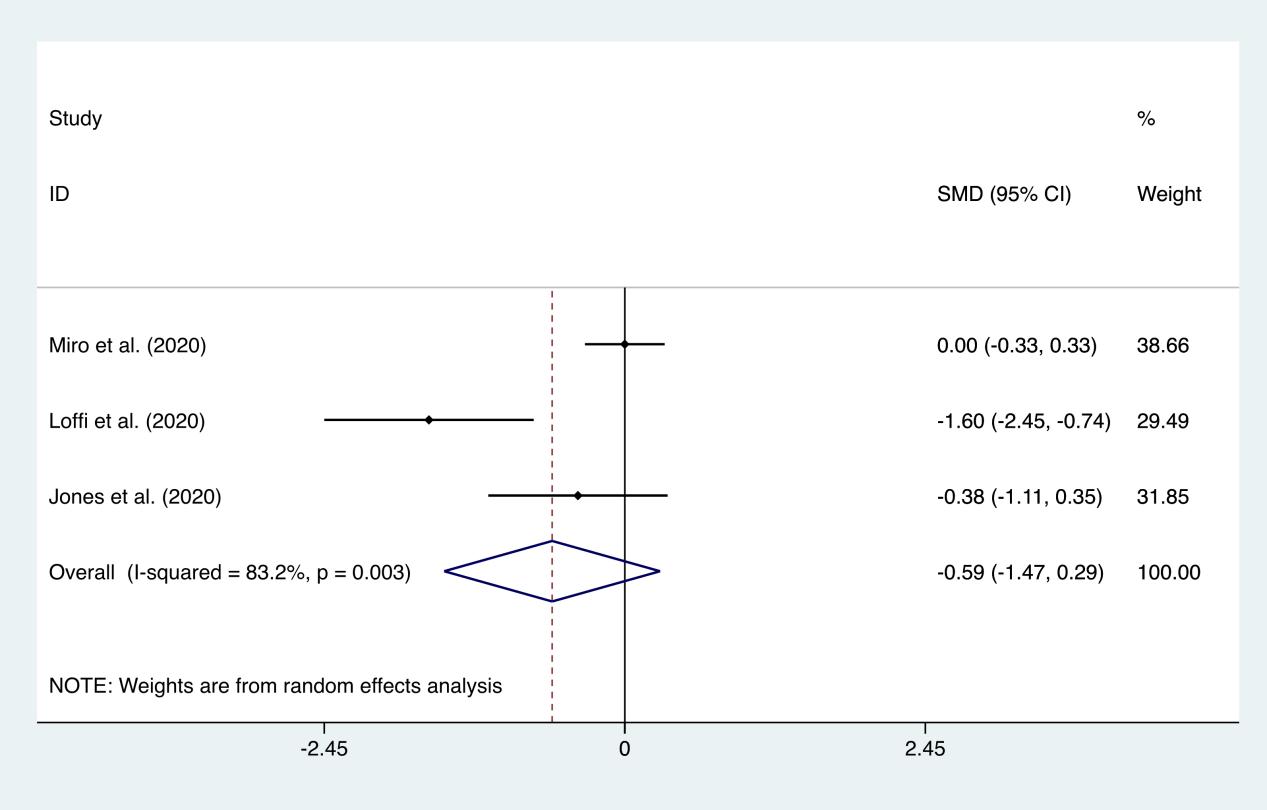


**Figure S21**: Forest plot of differences in creatinine levels between COVID-19 patients with and without pulmonary air leak (number of events: 3).


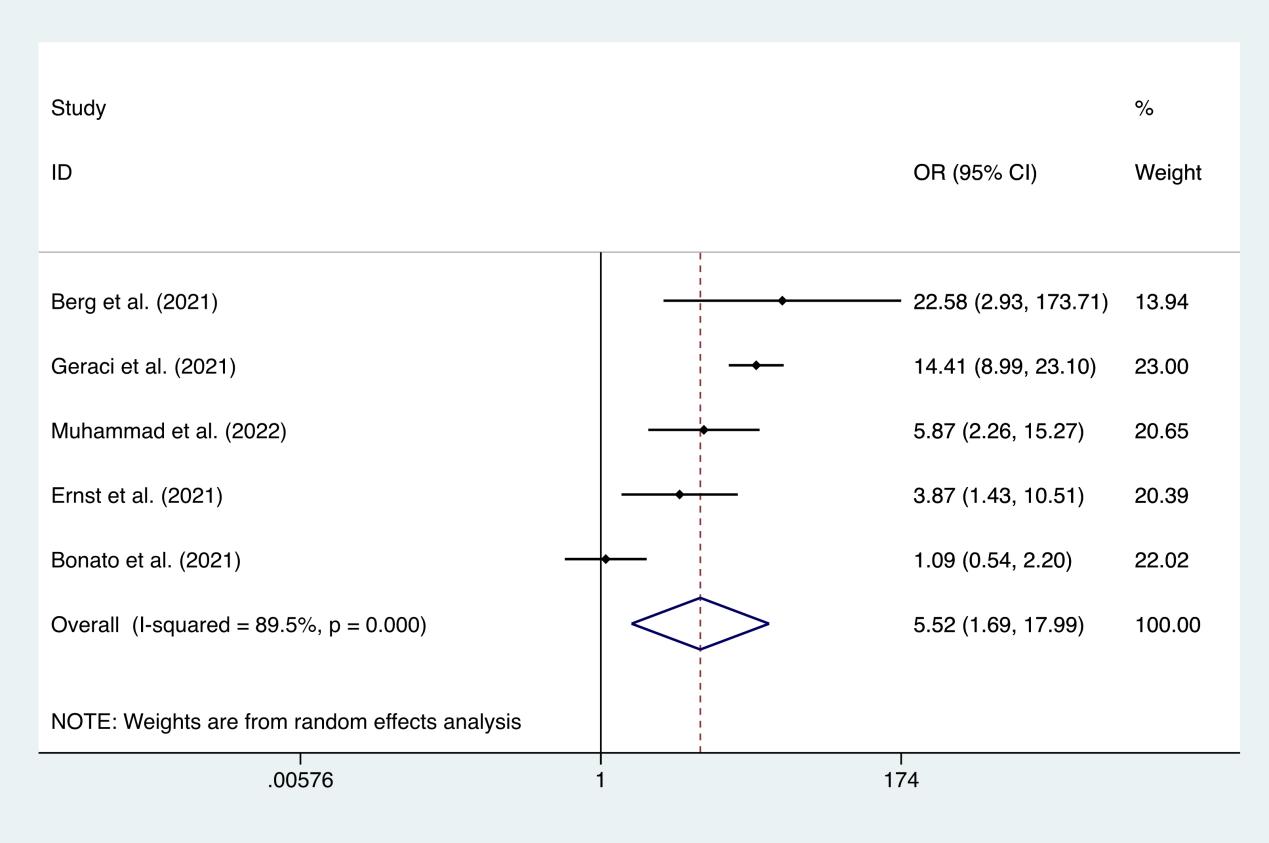


**Figure S22**: Forest plot of differences in mechanical ventilation between COVID-19

patients with and without pulmonary air leak (number of events: 5).


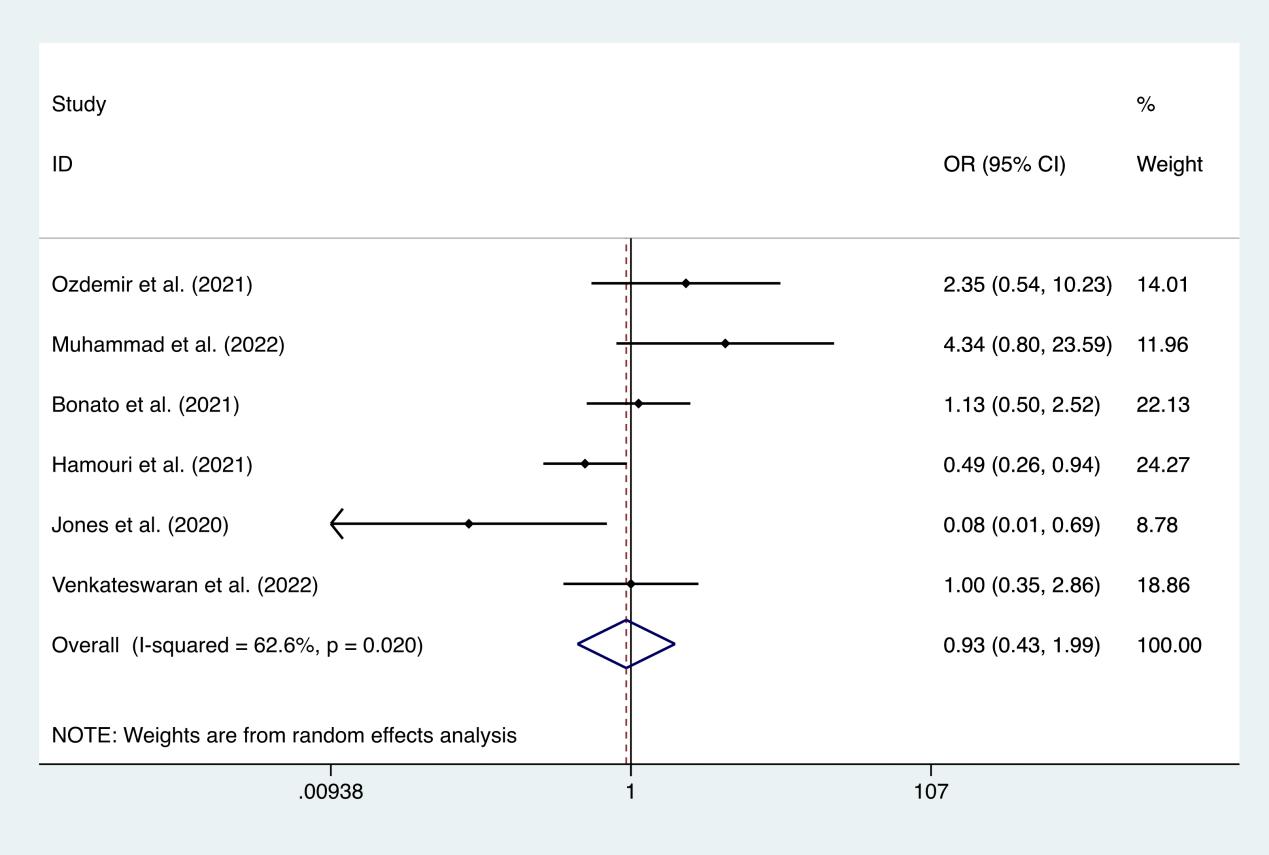


**Figure S23**: Forest plot of differences in invasive mechanical ventilation between

COVID-19 patients with and without pulmonary air leak (number of events: 6).


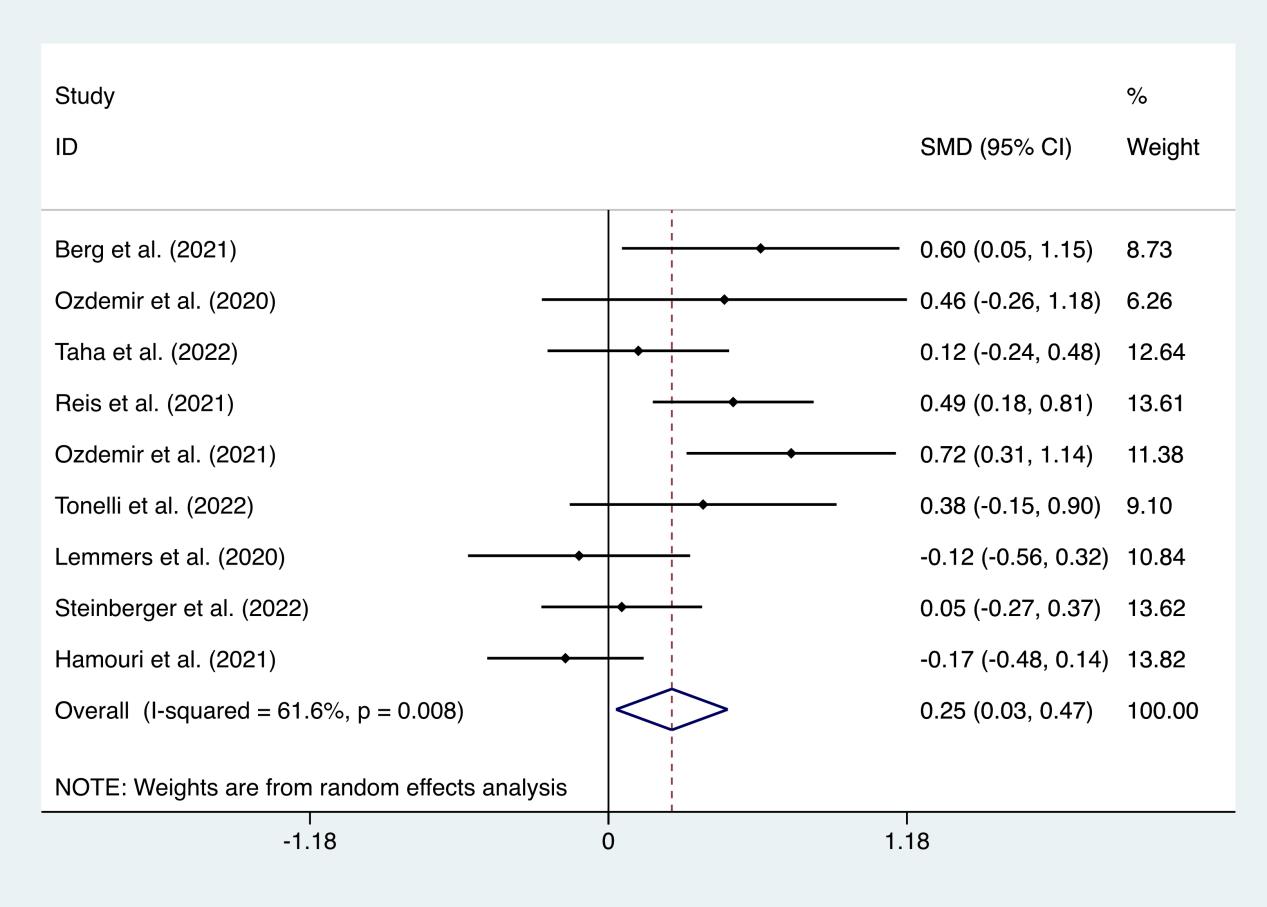


**Figure S24**: Forest plot of differences in positive end-expiratory pressure between

COVID-19 patients with and without pulmonary air leak (number of events: 9).


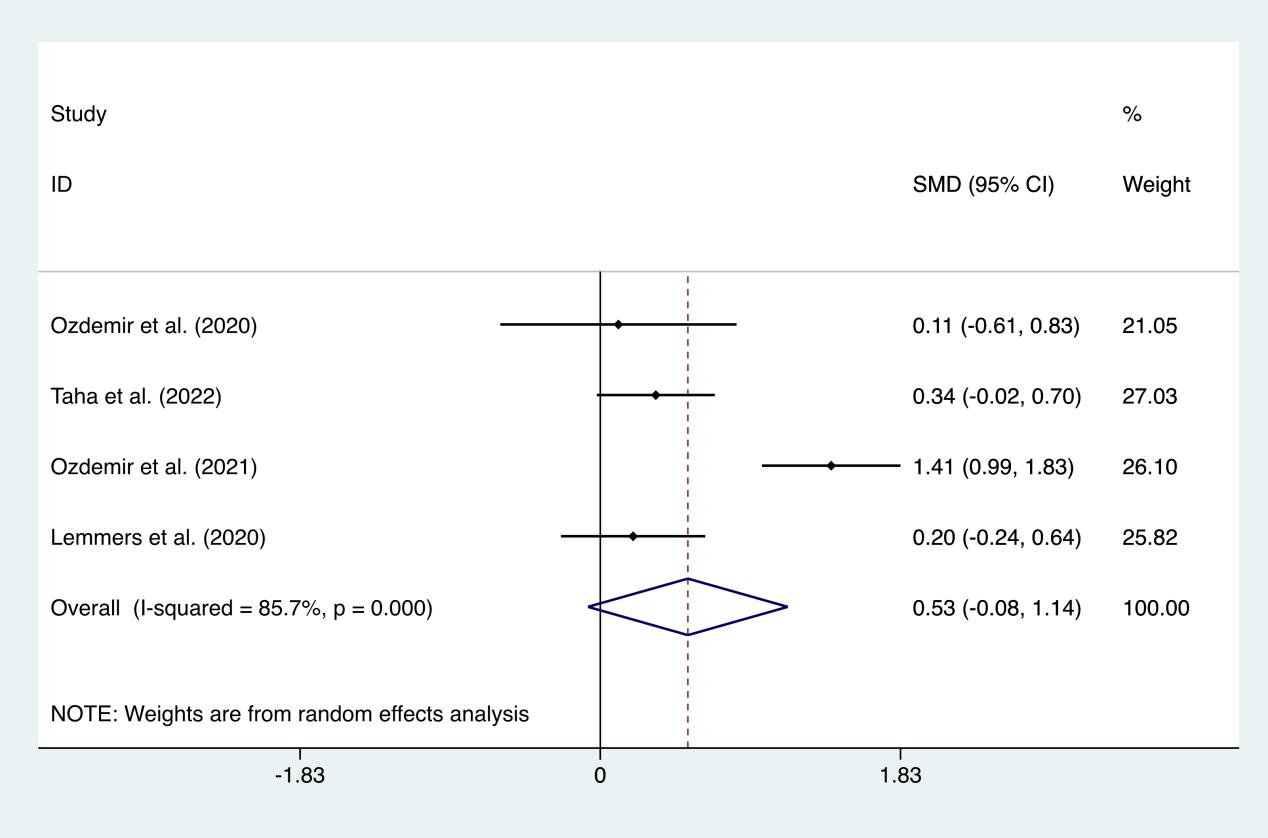


**Figure S25**: Forest plot of differences in peak inspiratory pressure between COVID-19

patients with and without pulmonary air leak (number of events: 4).


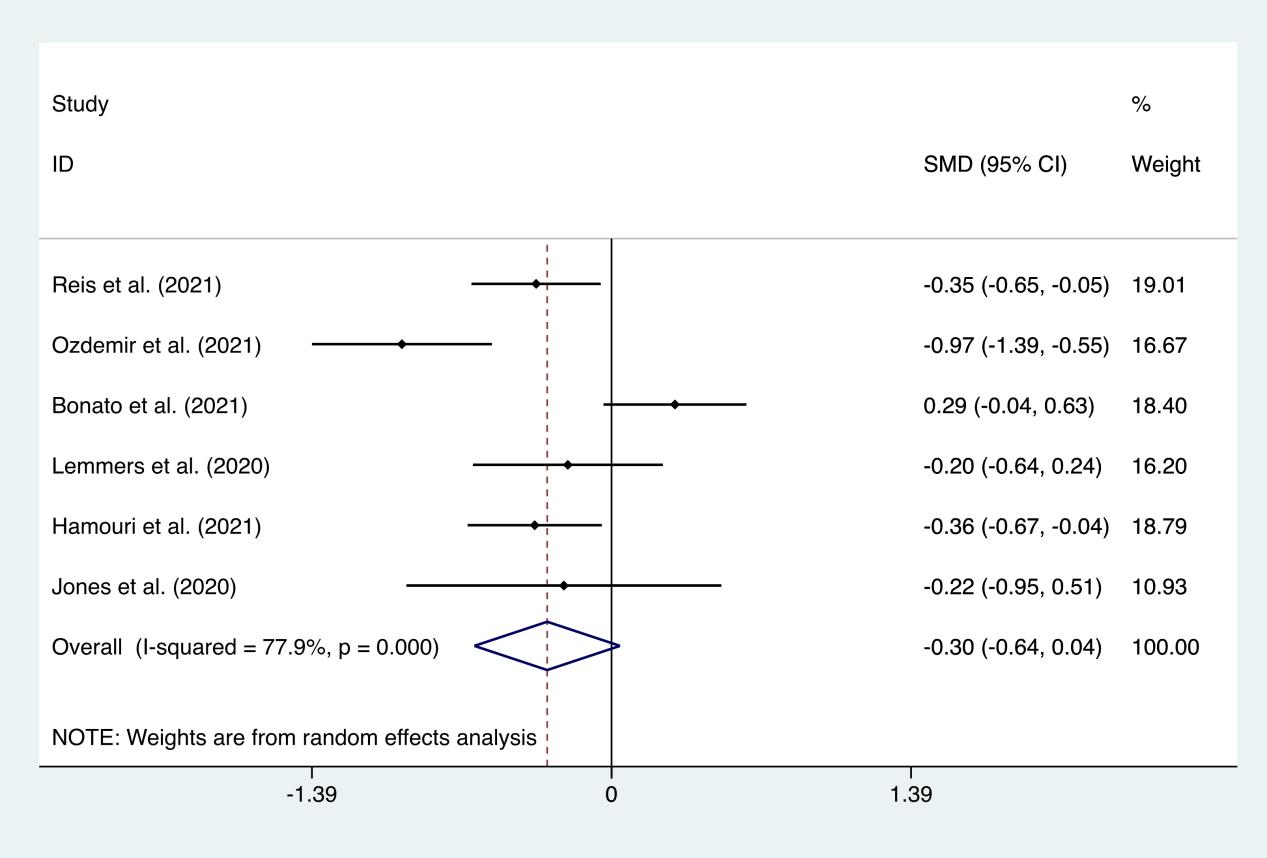


**Figure S26**: Forest plot of differences in PaO_2_/FiO_2_ ratio between COVID-19 patients

with and without pulmonary air leak (number of events: 6).


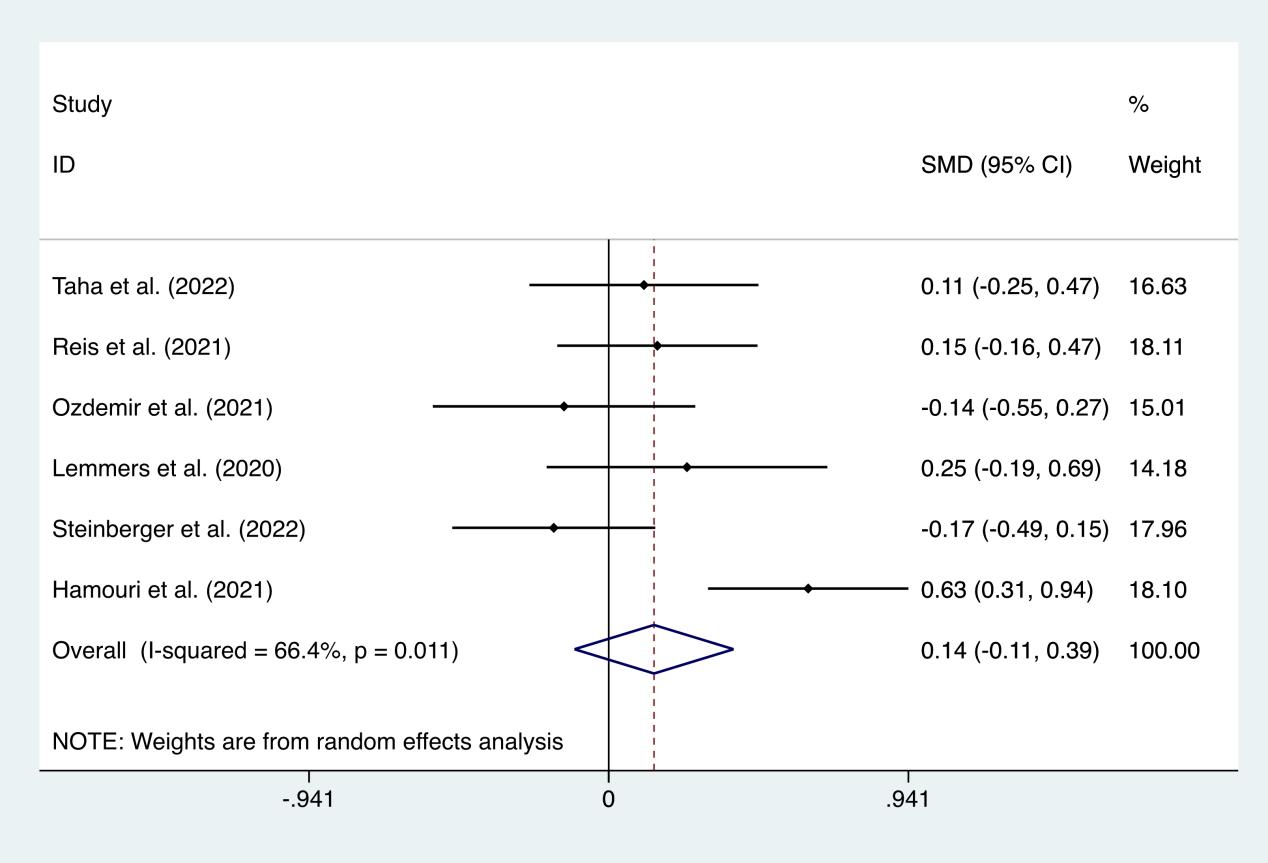


**Figure S27**: Forest plot of differences in tidal volume between COVID-19 patients with

and without pulmonary air leak (number of events: 6).


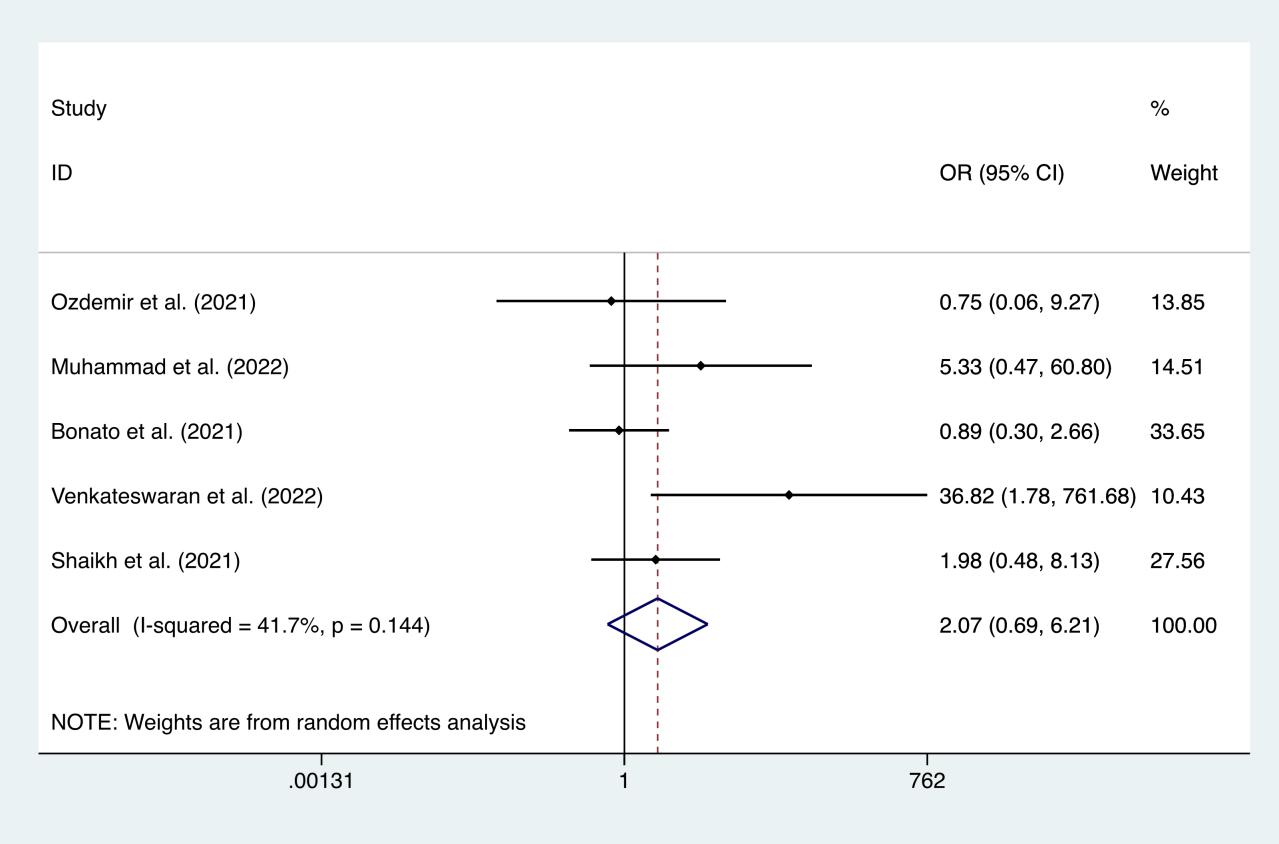


**Figure S28:** Forest plot of differences in the mortality rate between COVID-19 patients with multiple types of pulmonary air leak at the same time and those with only one type (number of events: 5).

**Table S1 :** The Newcastle–Ottawa Quality Assessment Scale.

| Study | Publication year | Selection | Comparability | Outcome | Quality assessment |
| --- | --- | --- | --- | --- | --- |
| Geraci et al. [12] | 2021 | 4 | 1 | 3 | good |
| Malik et al. [15] | 2022 | 4 | 1 | 3 | good |
| Pierre et al. [16] | 2022 | 4 | 1 | 2 | good |
| Akram et al. [17] | 2022 | 4 | 1 | 3 | good |
| Miro et al. [18] | 2020 | 4 | 2 | 3 | good |
| Berg et al. [19] | 2021 | 4 | 1 | 1 | fair |
| Chopra et al. [20] | 2021 | 4 | 1 | 3 | good |
| Ozdemir et al. [21] | 2020 | 4 | 1 | 3 | good |
| Capaccione et al. [22] | 2021 | 4 | 1 | 2 | good |
| Taha et al. [23] | 2022 | 4 | 1 | 3 | good |
| Reis et al. [24] | 2021 | 4 | 2 | 3 | good |
| Arciniega et al. [25] | 2021 | 4 | 1 | 3 | good |
| Ozdemir et al. [26] | 2021 | 4 | 1 | 3 | good |
| Ozsoy et al. [27] | 2021 | 4 | 1 | 3 | good |
| Baslas et al. [28] | 2022 | 4 | 1 | 3 | good |
| Loffi et al. [29] | 2020 | 4 | 1 | 3 | good |
| Righetti et al. [30] | 2022 | 4 | 1 | 1 | fair |
| Muhammad et al. [31] | 2022 | 4 | 1 | 3 | good |
| Tetaj et al. [32] | 2021 | 4 | 1 | 3 | good |
| Udwadia et al. [33] | 2021 | 4 | 2 | 3 | good |
| Gazivoda et al. [34] | 2021 | 4 | 1 | 3 | good |
| Belletti et al. [35] | 2021 | 4 | 1 | 3 | good |
| Ernst et al. [36] | 2021 | 4 | 1 | 1 | fair |
| Bonato et al. [37] | 2021 | 4 | 1 | 3 | good |
| Tonelli et al. [38] | 2022 | 4 | 1 | 3 | good |
| Marza et al. [39] | 2022 | 4 | 1 | 3 | good |
| Shaikh et al. [40] | 2021 | 4 | 1 | 3 | good |
| Lemmers et al. [41] | 2020 | 4 | 2 | 3 | good |
| Steinberger et al. [42] | 2022 | 4 | 1 | 3 | good |
| Nespoli et al. [43] | 2020 | 4 | 1 | 1 | fair |
| Hamouri et al. [44] | 2021 | 4 | 1 | 3 | good |
| Guven et al. [45] | 2021 | 4 | 1 | 3 | good |
| Jones et al. [46] | 2020 | 4 | 1 | 3 | good |
| Venkateswaran et al. [47] | 2022 | 4 | 1 | 3 | good |
| Sami et al. [48] | 2022 | 4 | 1 | 3 | good |

**Table S2:** Age, sex, hospital admission information and mortality of COVID-19 patients with and without pulmonary air leak.

| Author | COVID-19 with PAL | | | | | | | COVID-19 with non-PAL | | | | | | |
| --- | --- | --- | --- | --- | --- | --- | --- | --- | --- | --- | --- | --- | --- | --- |
|  | Number | Age  (years) | Male | ICU stay（days） | ICU patients | Hospital stay （days） | Death | Number | Age | Male | ICU stay（days） | ICU patients | Hospital stay （days） | Death |
| Geraci et al. [12] | 118 | 66.6±11.3 | 90 | NA | 110 | NA | 69 | 1477 | 64.3±13.4 | 931 | NA | 329 | NA | 198 |
| Malik et al. [15] | 1915 | 62.6±15.3 | 1262 | NA | NA | 26±23 | 938 | 101,943 | 61.9 ±17.8 | 51,675 | NA | NA | 8±9 | 10,526 |
| Pierre et al. [16] | 21 | 67.4±16.5 | 18 | NA | 13 | NA | 11 | 1631 | 72.3±17.1 | 897 | NA | 279 | NA | 269 |
| Akram et al. [17] | 75 | NA | NA | 39±33 | NA | NA | 40 | 1713 | NA | NA | 14±21 | NA | NA | 236 |
| Miro et al. [18] | 40 | 62.1±20.8 | 29 | NA | 13 | NA | 13 | 400 | 61.4±23.1 | 205 | NA | 7 | NA | 55 |
| Berg et al. [19] | 16 | NA | NA | NA | NA | NA | 9 | 243 | NA | NA | NA | NA | NA | 68 |
| Chopra et al. [20] | 80 | 58.0±16.0 | 59 | 31±22 | NA | 42±28 | 50 | 160 | 61.0±16.0 | 101 | 15±13 | NA | 21±17 | 78 |
| Ozdemir et al. [21] | 8 | 58.9±9.4 | 8 | NA | NA | NA | 4 | 99 | 60.4±14.3 | 62 | NA | NA | NA | 68 |
| Capaccione et al. [22] | 18 | 55.6±15.1 | 15 | NA | NA | NA | NA | 114 | 63.7±11.4 | 68 | NA | NA | NA | NA |
| Taha et al. [23] | 33 | 59.0 ± 13.0 | 17 | 43±75 | NA | 59±96 | 10 | 301 | 61 ± 14 | 169 | 31±58 | NA | 36±66 | 129 |
| Reis et al. [24] | 87 | 59.0±14.4 | 52 | NA | 69 | NA | 59 | 87 | 63.0±13.6 | 60 | NA | NA | NA | 52 |
| Arciniega et al. [25] | 9 | 57.0 ±15.3 | 7 | NA | NA | 17 ±14 | 3 | 262 | 59.5 ±15.4 | 165 | NA | NA | 12 ±7 | 96 |
| Ozdemir et al. [26] | 24 | 51.2± 16.1 | 19 | NA | NA | NA | 20 | 403 | 60.4 ± 15.9 | 269 | NA | NA | NA | 227 |
| Ozsoy et al. [27] | 20 | 57.7±14.1 | 10 | NA | NA | 20±12 | NA | 50 | 60.6±13.6 | 22 | NA | NA | 10±5 | NA |
| Baslas et al. [28] | 13 | NA | NA | NA | NA | NA | 9 | 281 | NA | NA | NA | NA | NA | 72 |
| Loffi et al. [29] | 6 | 66.3±29.6 | 5 | NA | NA | NA | 1 | 96 | 64.0±16.6 | 68 | NA | NA | NA | 11 |
| Muhammad et al. [31] | 19 | 58.3±13.6 | 11 | NA | 16 | 20±22 | 10 | 1029 | 72.6±19.3 | 570 | NA | 163 | 8±7 | 363 |
| Tetaj et al. [32] | 36 | 63.0±12.2 | 29 | NA | NA | NA | 17 | 461 | 65.4±12.6 | 340 | NA | NA | NA | 172 |
| Udwadia et al. [33] | 42 | NA | NA | NA | NA | NA | 31 | 1282 | NA | NA | NA | NA | NA | 218 |
| Gazivoda et al. [34] | 75 | 61.7±16.6 | 50 | 14±13 | NA | 18±18 | 54 | 206 | 68.3±11.9 | 142 | 12±10 | NA | 16±13 | 154 |
| Belletti et al. [35] | 28 | 63.1±10.2 | 23 | 31±29 | NA | 47±32 | 17 | 88 | 61.6±11.3 | 75 | 14±10 | NA | 29±22 | 34 |
| Ernst et al. [36] | 25 | NA | NA | NA | 24 | NA | 17 | 100 | NA | NA | NA | 86 | NA | 43 |
| Bonato et al. [37] | 53 | NA | NA | NA | NA | 24±13 | 25 | 106 | NA | NA | NA | NA | 18±13 | 33 |
| Tonelli et al. [38] | 28 | NA | 18 | 17±16 | NA | 26±28 | 16 | 28 | NA | 19 | 12±12 | NA | 23±27 | 6 |
| Marza et al. [39] | 30 | 64.4 ± 12.2 | 21 | NA | 14 | 22±25 | 14 | 140 | 59.1± 14.1 | 72 | NA | 18 | 13±7 | 26 |
| Lemmers et al. [41] | 23 | 64.7±7.9 | 15 | 13±12 | NA | 19±13 | 13 | 146 | 65.6±9.0 | 118 | 11±10 | NA | 15±11 | 73 |
| Steinberger et al. [42] | 43 | 62.3±12.3 | 29 | NA | NA | NA | 33 | 320 | 66.6±12.7 | 197 | NA | NA | NA | 224 |
| Nespoli et al. [43] | 16 | 66.2±12.2 | NA | 12±8 | NA | NA | 10 | 65 | 60.1±11.4 | NA | 12±9 | NA | NA | 23 |
| Hamouri et al. [44] | 51 | 60.7 ±15.9 | 29 | NA | NA | 14 ±9 | 46 | 188 | 67.6±12.6 | 117 | NA | NA | 13±9 | 164 |
| Guven et al. [45] | 9 | NA | NA | NA | NA | NA | 4 | 65 | NA | NA | NA | NA | NA | 19 |
| Jones et al. [46] | 8 | 49.3±17.5 | 8 | NA | NA | NA | 5 | 75 | 57.8±11.2 | 53 | NA | NA | NA | 34 |
| Venkateswaran et al. [47] | 30 | NA | NA | 17±17 | NA | NA | 25 | 30 | NA | NA | 12±9 | NA | NA | 13 |
| Sami et al. [48] | 13 | 57.9±16.7 | NA | NA | NA | 27±8.2 | NA | 90 | 69.3±15.2 | NA | NA | NA | 14±8 | NA |

COVID-19: coronavirus disease 2019, ICU: [intensive care unit](javascript:;), PAL: pulmonary air leak, NA: not applicable.

**Table S3 :** Comorbidities of COVID-19 patients with and without pulmonary air leak.

| Author | COVID-19 with PAL | | | | | | | COVID-19 with non-PAL | | | | | | |
| --- | --- | --- | --- | --- | --- | --- | --- | --- | --- | --- | --- | --- | --- | --- |
|  | Number | Diabetes | Hypertension | COPD | Asthma | Cancer | Smoking | Number | Diabetes | Hypertension | COPD | Asthma | Cancer | Smoking |
| Geraci et al. [12] | 118 | 42 | 61 | NA | NA | 6 | 30 | 1477 | 517 | 915 | NA | NA | 118 | 372 |
| Malik et al. [15] | 1915 | 349  (*1399) | 821  (*1757) | 146  (*781) | 137  (*1757) | 45  (*1757) | 725  (*1757) | 101943 | 21925  (*82196) | 49382  (*96781) | 8951  (*45863) | 8537  (*96781) | 1800  (*96781) | 38784  (*96781) |
| Pierre et al. [16] | 21 | 5 | NA | NA | NA | 4 | 2  (*9) | 1631 | 449 | NA | NA | NA | 387 | 192 (*547) |
| Miro et al. [18] | 40 | 7 | 15 | 4 | 8 | 5 | 4 | 400 | 74 | 168 | 23 | 27 | 38 | 26 |
| Chopra et al. [20] | 80 | 24 | 23 | NA | NA | 1 | NA | 160 | 72 | 39 | NA | NA | 7 | NA |
| Taha et al. [23] | 33 | 23 | 27 | 7 | 3 | 3 | 7 | 301 | 177 | 239 | 68 | 31 | 42 | 73 |
| Reis et al. [24] | 87 | 34 | 49 | 2 | 16 | NA | NA | 87 | 44 | 58 | 12 | 11 | NA | NA |
| Arciniega et al. [25] | 9 | 3 | 5 | NA | NA | NA | 1 | 262 | 110 | 154 | NA | NA | NA | 36 |
| Ozsoy et al. [27] | 20 | 7 | 5 | 0 | 9 | NA | NA | 50 | 22 | 32 | 7 | NA | NA | NA |
| Loffi et al. [29] | 6 | 0 | 1 | NA | NA | NA | 2 | 96 | 11 | 37 | NA | NA | NA | 25 |
| Muhammad et al. [31] | 19 | NA | NA | 0 | 6 | NA | NA | 1029 | NA | NA | 64 | 49 | NA | NA |
| Tetaj et al. [32] | 36 | 5 | NA | NA | NA | 3 | NA | 461 | 74 | NA | NA | NA | 29 | NA |
| Gazivoda et al. [34] | 75 | 38 | 40 | 2 | 10 | NA | NA | 206 | 109 | 129 | 15 | 12 | NA | NA |
| Belletti et al. [35] | 28 | 5 | 8 | 1 | 0 | 1 | 3 | 88 | 14 | 44 | 1 | 4 | NA | 9 |
| Bonato et al. [37] | 53 | 8 | 25 | 3 | 3 | NA | 23 | 106 | 24 | 61 | 9 | 3 | NA | 52 |
| Marza et al. [39] | 30 | 4 | 19 | 4 | 4 | 2 | 6 | 140 | 35 | 96 | 3 | 6 | 3 | 49 |
| Lemmers et al. [41] | 23 | 3 | 11 | 0 | NA | NA | NA | 146 | 23 | 79 | 4 | NA | NA | NA |
| Steinberger et al. [42] | 43 | 4 | 3 | 0 | 0 | 1 | 3 | 320 | 93 | 119 | 10 | 13 | 15 | 80 |
| Hamouri et al. [44] | 51 | 17 | 29 | 0 | 0 | 4 | 12 | 188 | 102 | 136 | 5 | 5 | 14 | NA |
| Jones et al. [46] | 8 | NA | NA | 0 | 0 | NA | 2 | 75 | NA | NA | 0 | NA | NA | 21 |
| Venkateswaran et al. [47] | 30 | 13 | 12 | NA | NA | NA | NA | 30 | 8 | 14 | NA | NA | NA | NA |

COVID-19: coronavirus disease 2019, PAL: pulmonary air leak, COPD: chronic obstructive pulmonary disease, NA: not applicable. *: corresponding sample size.

**Table S4a:** Laboratory findings of COVID-19 patients with pulmonary air leak.

| Author | COVID-19 with PAL | | | | | | | | | | |
| --- | --- | --- | --- | --- | --- | --- | --- | --- | --- | --- | --- |
|  | Number | Leucocytes | Neutrophils | Lymphocytes | C-reactive protein | Ferritin | Platelets | D-dimer | Creatinine | Aspartate  aminotransferase | Lactate dehydrogenase |
| Pierre et al. [16] | 21 | 7.7±4.6 (g/L)  (*20) | 6.2±4.8 (g/L) (*20) | 0.7±0.4 (g/L) (*20) | 128.6±169.1 (mg/L)  (*20) | NA | NA | NA | NA | 51.2±27.4 (IU/L)  (*13) | 388.0±163.6 (IU/L)  (*9) |
| Miro et al. [18] | 40 | 10.0±3.4 (cells/mL) | NA | 0.9±0.5 (cells/mL) | 9.5±15.1 (mg/dL ) | NA | NA | 2136.3±2764.3(ng/mL) | 0.9±0.3 (mg/dL) | 42.5±32.3 (IU/L) | 408.8±256.8 (IU/L) |
| Taha et al. [23] | 33 | NA | NA | NA | 269.6±461.1 (mg/L) | 2842.1±5796.7 (ng/mL) | NA | NA | NA | NA | NA |
| Reis et al. [24] | 87 | NA | NA | NA | NA | NA | NA | NA | NA | NA | 845.4±414.2 (IU/L) |
| Arciniega et al. [25] | 9 | 14.7±8.6 (109/L) | NA | 0.9±0.3 (109/L) | 119.8±88.0 (mg/L) | 1440.2±852.7 (ng/mL) | 277.7±107.2  (109/L) | 5441.1±7686.4(ng/mL) | NA | NA | 558.5±272.5 (IU/L) |
| Ozsoy et al. [27] | 20 | NA | NA | NA | NA | 1061.6±507.4 (ug/L) | NA | NA | NA | NA | NA |
| Loffi et al. [29] | 6 | 12111.0±14621.5 (103/mm3) | NA | 22.5±31.5 (%) | 140.7±214.5 (mg/L) | NA | 161.7±69.6  (103/mm3) | 4.3±4.9  (mcg/mL) | 0.9±0.5 (mg/dL) | 61.1±56.3 (U/L) | 236±131.6 (U/L) |
| Muhammad et al. [31] | 19 | NA | NA | NA | 125.7±65.7 (ng/mL) | NA | NA | NA | NA | NA | NA |
| Bonato et al. [37] | 53 | NA | 6419.3±2987.2(cell/mcL) | 664.7±301.0  (cell/mcL) | 9.2±8.2 (mg/dL) | NA | 232.3±138.7 (106 cell/mcL) | 1318.2±922.8 (ng/mL) | NA | NA | NA |
| Tonelli et al. [38] | 28 | 8.5±5.5 (109/L ) | NA | NA | 11.9±9.0 (mg/dL) | NA | NA | 5406.1±7274.1(µg/L) | NA | NA | NA |
| Marza et al. [39] | 30 | 10.9±7.3 (103/µL) | NA | 1.0±0.5  (103/µL) | 99.0±69.3 (no units) | NA | NA | 1848.7±1531.9 (ng/mL) | NA | NA | 383.5±301.2 (units/L)  (*15) |
| Hamouri et al. [44] | 51 | 12.0±7.4  (no units) | 9810.0±5260.0(no units) | 1630.0±6100.0 (no units) | 165.0±101.0  (no units) | 897.0±559.0 (no units) | NA | 5.1±6.2  (no units) | NA | NA | 1250.0±524.0  (no units) |
| Jones et al. [46] | 8 | NA | 7.2±1.1 (109/L) | 0.8±0.5 (109/L) | 202.9±61.7 (mg/L) | NA | NA | NA | 81.6±24.1 (µmol/L) | NA | NA |
| Sami et al. [48] | 13 | NA | NA | NA | NA | NA | NA | NA | NA | NA | 1027±354 (μ/L) |

COVID-19: coronavirus disease 2019, PAL: pulmonary air leak, COPD: chronic obstructive pulmonary disease, NA: not applicable. *: corresponding sample size.

**Table S4b:** Laboratory findings of COVID-19 patients without pulmonary air leak.

| Author | COVID-19 with non-PAL | | | | | | | | | | |
| --- | --- | --- | --- | --- | --- | --- | --- | --- | --- | --- | --- |
|  | Number | Leucocytes | Neutrophils | Lymphocytes | C-reactive protein | Ferritin | Platelets | D-dimer | Creatinine | Aspartate  aminotransferase | Lactate dehydrogenase |
| Pierre et al. [16] | 1631 | 6.8±3.2 (g/L)  (*1628) | 5.1±2.9 (g/L)  (*1603) | 0.9±0.6  (*1593) | 60.2±65.3 (mg/L)  (*1599) | NA | NA | NA | NA | 39.5±23.0 (IU/L)  (*1067) | 282.5±111.4 (IU/L)  (*826) |
| Miro et al. [18] | 400 | 6.9±3.0  (cells/mL ) | NA | 1.2±0.6  (cells/mL ) | 6.2±7.2 (mg/dL) | NA | NA | 737.1±644.3 (ng/mL) | 0.9±0.3 (mg/dL) | 32.1±16.4 (IU/L) | 277.1±109.4 (IU/L) |
| Taha et al. [23] | 301 | NA | NA | NA | 672.3±1337.1 (mg/L) | 2788.1±5578.6 (ng/mL) | NA | NA | NA | NA | NA |
| Reis et al. [24] | 87 | NA | NA | NA | NA | NA | NA | NA | NA | NA | 793.0±294.0 (IU/L) |
| Arciniega et al. [25] | 262 | 11.0±5.7  (10^9^/L) | NA | 0.9±0.5  (10^9^/L) | 156.3±107.3 (mg/L) | 1084.6±852.7 (ng/mL) | 266.0±111.8 (10^9^/L) | 2350.2±6780.9( ng/mL) | NA | NA | 484.3±265.6 (IU/L) |
| Ozsoy et al. [27] | 50 | NA | NA | NA | NA | 377.1±291.0 (ug/L) | NA | NA | NA | NA | NA |
| Loffi et al. [29] | 96 | 6061.3±2565.1  (10^3^/mm^3^) | NA | 1.4±5.3(%) | 61.6±76.0 (mg/L) | NA | 191.8±85.1(10^3^/mm^3^) | 2.7±4.2 (mcg/mL) | 1.0±0.3 (mg/dL) | 40.6±24.8 (U/L) | 362.8±190.0 (U/L) |
| Muhammad et al. [31] | 1029 | NA | NA | NA | 76.3±71.3 (ng/mL) | NA | NA | NA | NA | NA | NA |
| Bonato et al. [37] | 106 | NA | 5943.9±3164.1 (cell/mcL) | 802.2±420.9 (cell/mcL) | 10.7±11.3 (mg/dL) | NA | 250.0±112.0 (10^6^cell/mcL) | 997.1±758.3 ( ng/mL) | NA | NA | NA |
| Tonelli et al. [38] | 28 | 6.8±3.8  (10^9^/L) | NA | NA | 11.0±13.6 (mg/dL) | NA | NA | 1276.0±510.1 ( µg/L) | NA | NA | NA |
| Marza et al. [39] | 140 | 8.7±6.6  (10^3^/µL) | NA | 0.9±0.6  (10^3^/µL) | 86.6±93.7  (no units) | NA | NA | 751.6±535.6 (ng/mL) | NA | NA | 340.3±158.4 (units/L) |
| Hamouri et al. [44] | 188 | 10.2±6.11  (no units) | 8690.0±5210.0  (no units) | 1040.0±1660.0  (no units) | 168.0±95.9  (no units) | 779.0±692.0 (no units) | NA | 3.6±5.2  (no units) | NA | NA | 1070.0±485.0  (no units) |
| Jones et al. [46] | 75 | NA | 8.0±3.9 (10^9^/L) | 0.9±0.4  (10^9^/L) | 183.5±98.3  (mg/L) | NA | NA | NA | 91.9±27.2 (µmol/L) | NA | NA |
| Sami et al. [48] | 90 | NA | NA | NA | NA | NA | NA | NA | NA | NA | 946±733 (μ/L) |

COVID-19: coronavirus disease 2019, PAL: pulmonary air leak, COPD: chronic obstructive pulmonary disease, NA: not applicable. *: corresponding sample size.

**Table S5:**  Mechanical ventilation of COVID-19 patients with and without pulmonary air leak.

| Author | COVID-19 with PAL | | | | | | | COVID-19 with non-PAL | | | | | | |
| --- | --- | --- | --- | --- | --- | --- | --- | --- | --- | --- | --- | --- | --- | --- |
|  | Number | MV | IMV | PEEP (cmH_2_O) | Peak inspiratory pressure (cmH_2_O) | PaO_2_/FiO_2_ (mmHg) | Tidal volume | Number | MV | IMV | PEEP (cmH_2_O) | Peak inspiratory pressure (cmH_2_O) | PaO_2_/FiO_2_ (mmHg) | Tidal volume |
| Geraci et al. [12] | 118 | 95 | NA | NA | NA | NA | NA | 1477 | 329 | NA | NA | NA | NA | NA |
| Berg et al. [19] | 16 | 15 | NA | 14.7±6.8 | NA | NA | NA | 243 | 97 | NA | 12.2±3.6 | NA | NA | NA |
| Ozdemir et al. [21] | 8 | 8 | 8 | 9.8 ± 0.4 | 27.1±4.9 | NA | NA | 99 | 99 | 99 | 9.0±1.8 | 26.6±4.5 | NA | NA |
| Capaccione et al. [22] | 18 | 18 | 18 | NA | NA | NA | NA | 114 | 114 | 114 | NA | NA | NA | NA |
| Taha et al. [23] | 33 | 33 | NA | 9.9 ± 3.2 | 30 ± 5 | NA | 6.2±1.9  (mL/kg) | 301 | 301 | NA | 9.5 ± 3.4 | 28 ± 6 | NA | 6±1.8  ( mL/kg ) |
| Reis et al. [24] | 87 | 78 | 78 | 13.7 ± 4.3 | NA | 137.1 ± 73.4 | 6.43 ± 0.9  (no units) | 87 | 78 | 78 | 11.5 ± 4.6 | NA | 169.6 ± 109.2 | 6.30 ± 0.8  (no units) |
| Ozdemir et al. [26] | 24 | 24 | 22 | 9.8 ± 1.0 | 32.3 ± 6.6 | 111.7 ± 51.5 | 445.6 ± 102.3 (no units) | 403 | 403 | 332 | 8.8 ± 1.4 | 25.9 ± 4.4 | 176.6 ± 67.6 | 457.4 ± 83.0 (no units) |
| Muhammad et al. [31] | 19 | 7 | 5 | NA | NA | NA | NA | 1029 | 93 | 34 | NA | NA | NA | NA |
| Ernst et al. [36] | 25 | 19 | NA | NA | NA | NA | NA | 100 | 45 | NA | NA | NA | NA | NA |
| Bonato et al. [37] | 53 | 36 | 17 | NA | NA | 214.0±121.1 | NA | 106 | 70 | 31 | NA | NA | 180.3±111.2 | NA |
| Tonelli et al. [38] | 28 | 28 | 0 | 9.3±1.6 | NA | NA | NA | 28 | 28 | 0 | 8.7±1.6 | NA | NA | NA |
| Lemmers et al. [41] | 23 | 23 | 23 | 11.6±5.5 | 30.0±5.0 | 107.9±44.3 | 6.1±0.9  (mL/kg) | 146 | 146 | 146 | 12.0±3.0 | 29.0±5.0 | 117.9±50.2 | 5.9±0.8 (mL/kg) |
| Steinberger et al. [42] | 43 | 43 | NA | 14.6±2.3 | NA | NA | 432.8±52.2 (no units) | 320 | 320 | NA | 14.3±6.0 | NA | NA | 443.0±60.0 (no units) |
| Hamouri et al. [44] | 51 | 51 | 17 | 8.8±3.6 | NA | 67.5±45.5 | 6.6 ±0.7 (mL/kg) | 188 | 188 | 95 | 9.4±3.5 | NA | 85.2 ±50.8 | 6.3 ±0.4 |
| Jones et al. [46] | 8 | 8 | 1 | NA | NA | 74.5±12.5 | NA | 75 | 75 | 48 | NA | NA | 79.5±23.4 | NA |
| Venkateswaran et al. [47] | 30 | 30 | 11 | NA | NA | NA | NA | 30 | 30 | 11 | NA | NA | NA | NA |

COVID-19: coronavirus disease 2019, PAL: pulmonary air leak, MV: mechanical ventilation, IMV: invasive mechanical ventilation, PEEP: positive end-expiratory pressure, NA: not applicable.

**Table S6:** Characteristics of COVID-19 patients with multiple types of pulmonary air leak at the same time and those with only one type.

| Author | Multiple types of PAL | | | Only one type of PAL | | |
| --- | --- | --- | --- | --- | --- | --- |
|  | Types of PAL | Number | Death | Types of PAL | Number | Death |
| Ozdemir et al.[26] | pneumothorax/pneumomediastinum | 5 | 4 | pneumomediastinum | 19 | 16 |
| Muhammad et al. [31] | pneumothorax/pneumomediastinum | 5 | 4 | pneumothorax/pneumomediastinum | 14 | 6 |
| Bonato et al. [37] | pneumothorax/pneumomediastinum | 22 | 10 | pneumothorax/pneumomediastinum | 31 | 15 |
| Shaikh et al. [40] | pneumothorax/pneumomediastinum | 11 | 7 | pneumothorax/pneumomediastinum | 32 | 15 |
| Venkateswaran et al. [47] | pneumothorax/pneumomediastinum/  subcutaneous emphysema | 7 | 7 | pneumothorax/subcutaneous emphysema | 18 | 5 |

COVID-19: coronavirus disease 2019, PAL: pulmonary air leak.
